# Developmental priming of adult proteostasis and longevity by NuA4 complex activity in early life

**DOI:** 10.1038/s44318-026-00851-8

**Published:** 2026-07-16

**Authors:** Yihan Wang, Xiong Xiong, Runshuai Zhang, Long Xiao, Xinyu Ruan, Tianyi Ni, Zicheng Liu, Jie Chen, Shenlu Qin, Zhuo Du, Yanxiao Zhang, Lianfeng Wu

**Affiliations:** 1 School of Life Sciences, Westlake University, Hangzhou, Zhejiang China; 2 Westlake Laboratory of Life Sciences and Biomedicine, Hangzhou, Zhejiang China; 3 Institute of Basic Medical Sciences, Westlake Institute for Advanced Study, Hangzhou, Zhejiang China; 4 Institute of Genetics and Developmental Biology, Chinese Academy of Sciences, Beijing, China; 5 University of Chinese Academy of Sciences, Beijing, China

**Keywords:** Chromatin, Transcription & Genomics, Molecular Biology of Disease, Translation & Protein Quality

## Abstract

Proteostasis collapse, a hallmark of aging and neurodegeneration like Alzheimer’s disease (AD), causes irreversible damage in late life. Whether late-life proteostasis capacity is developmentally programmed remains unclear, as mechanistic studies requiring lifelong tracking and molecular manipulation are challenging or impossible in long-lived species. Using *C. elegans* as a lifelong, genetically tractable AD model, we uncover a critical early-life window during which reducing TIP60/NuA4 acetyltransferase complex activity enduringly enhances proteostasis and extends lifespan. Mechanistically, NuA4 reduction depletes H4K16ac, triggering a compensatory, early-life-biased, XBP-1-mediated unfolded protein response (UPR^ER^). This UPR^ER^ activation remodels endoplasmic reticulum (ER) morphology and reprograms lipid metabolism, driving selective oleic acid (OA) accumulation. Crucially, this developmentally-installed OA reservoir confers lasting resilience against proteotoxic stress, an effect mimicked by OA supplementation. Together, these findings establish a chromatin-ER-lipid axis that developmentally primes adult proteostasis and suggest early-life interventions as a strategy to promote healthy aging and resilience to proteotoxic stress.

## Synopsis

Proteostasis capacity declines during aging, but whether it is programmed by events during early development remains poorly understood. This study shows that transient reduction of NuA4 complex activity during development establishes a UPR^ER^–lipid priming axis that durably enhances proteostasis and extends lifespan in *C. elegans*.

- Early-life, but not later-life, knockdown of NuA4 subunits protects against Aβ and α-synuclein-induced proteotoxicity and extends lifespan in *C. elegans*.
- Early-life reduction of NuA4-mediated H4K16ac elicits compensatory activation of the unfolded protein response (UPR) transcription factor XBP-1.
- XBP-1 activation remodels ER morphology and drives SBP-1/MDT-15-dependent FAT-7 upregulation, leading to selective oleic acid (OA) accumulation during development.
- Developmentally accumulated OA confers long-lasting proteostasis resilience in adulthood.

## Abstract (web summary)

Early-life reduction of NuA4 activity establishes a chromatin–ER–lipid program that confers long-lasting resistance to proteotoxic stress.

## Graphical abstract

**Figure:**
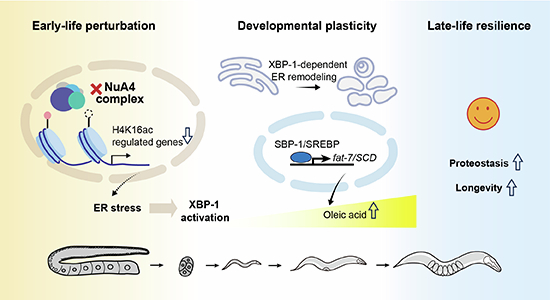

**Figure:**
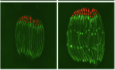

## Introduction

The maintenance of proteostasis, the balance of protein synthesis, folding, and degradation, is essential for cellular function and organismal health. With aging, proteostasis capacity declines, leading to the accumulation of misfolded or damaged proteins, a hallmark of age-related neurodegenerative diseases (Hipp et al, 2019). Alzheimer’s disease (AD) exemplifies this decline, characterized by amyloid-beta (Aβ) plaque accumulation (Chartier-Harlin et al, 1991), which drives progressive neuronal loss, cognitive decline, and functional impairment. Current therapeutic approaches, predominantly targeting late disease stages, show limited efficacy in reversing established proteotoxic damage (Zhang et al, 2024). Intriguingly, growing epidemiological evidence implicates early-life factors in modulating susceptibility to neurodegeneration. Perinatal neurodevelopment may influence vulnerability to late-onset AD, with potential risk factors including malnutrition, xenobiotics, and chemical exposures (Gauvrit et al, 2022). Similarly, older maternal age at birth shows a modest association with increased Parkinson’s disease (PD) risk (Gardener et al, 2010). These observations align with the Developmental Origin of Health and Diseases (DOHaD) paradigm (Barker, 2007; Bateson et al, 2004), which posits that environmental perturbations in early life can program susceptibility to late-life pathologies. Despite this compelling link, the causal mechanisms connecting developmental programming to aging-associated proteostasis collapse remain poorly understood, and the impracticality of longitudinal mammalian studies has hindered the development of early-life interventions designed to enhance late-life health.

*Caenorhabditis elegans* is a premier model for studying developmental plasticity (Qin et al, 2022; Zhang et al, 2023). Its simple anatomy, rapid lifecycle, and genetic tractability enable cost-effective, high-throughput studies of aging and proteostasis. The nematode’s well-defined developmental and aging timeline further facilitates dissection of signaling pathways across the lifespan and even generations (Zhang et al, 2021). Excellent examples include: early-life reactive oxygen species (ROS) suppressing H3K4me3 chromatin modifiers to promote longevity and stress resistance (Bazopoulou et al, 2019; Oleson et al, 2024); mitochondrial stress-induced longevity mediated by H3K27 demethylases and the NuRD histone deacetylase complex (Merkwirth et al, 2016; Zhu et al); and heat stress inducing persistent gene expression changes via H3K9me3 and the histone acetyltransferase CBP-1 (Klosin et al, 2017; Zhou et al, 2019; Jiang et al, 2024). Despite these advances, the potential mechanisms that developmentally prime late-life proteostasis maintenance remain poorly understood.

The highly conserved nucleosome acetyltransferase of H4 (NuA4) complex is a central regulator of chromatin remodeling and transcriptional regulation. Its core catalytic subunit, a MYST-family acetyltransferase, is conserved across species as *Esa1* in yeast, *TIP60* in mammals and *mys-1* in *C. elegans*. The mammalian TIP60 complex is essential for DNA repair, apoptosis, and cell cycle progression, and its dysregulation has been linked to cancer and neurodegeneration (Gorrini et al, 2007; Pollina et al, 2023). In *C. elegans*, NuA4 is found to acetylate histone H4 at lysine 16 (K16) (Lau et al, 2016; Ikeda et al, 2017), a modification known to facilitate gene expression (Liu et al, 2011; Samata et al, 2020). Depletion of NuA4 subunits results in developmental arrest and vulval morphogenesis defects (Ceol and Horvitz, 2004; Cui et al, 2006). Additionally, NuA4 enhances the activation of transcription factors such as DAF-16/FOXO, thereby promoting stress resistance and longevity (Ikeda et al, 2017). The complex also influences metabolic homeostasis, regulating lipid storage and energy utilization through chromatin-based control of metabolic gene expression (Hamsanathan et al, 2022). Collectively, these findings highlight the significant role of the NuA4 complex in development and aging. Its evolutionary conservation across eukaryotes positions NuA4 as a critical regulatory nexus linking chromatin structure to biological function, with *C. elegans* serving as a powerful model system for in vivo mechanistic dissection of these relationships.

In this study, we investigate whether organismal late-life proteostasis can be programmed in early life. Using *C. elegans* models expressing well-characterized pathological proteins associated with neurodegeneration for an unbiased screen, we revealed that inhibiting the NuA4 complex in early life enhances late-life proteostasis and extends lifespan. Notably, such modulation needs a critical early-life time window to enhance proteostasis, as perturbation in later stages results in adverse effects. Our results demonstrated that this beneficial effect depends on the X-box binding protein 1 (XBP-1), a key transcription factor in the unfolded protein response (UPR). The NuA4 complex regulates UPR genes through H4K16 acetylation; loss of this modification triggers compensatory activation of XBP-1. This activation subsequently induces remodeling of the endoplasmic reticulum (ER) and activates the SBP-1-FAT-7-oleic acid synthesis pathway. These signaling events, initiated during development, reprogram oleic acid levels and ultimately confer protection against proteotoxic stress in late life. Our findings suggest potential strategies for early-life interventions in adult-onset diseases linked to proteostasis dysfunction.

## Results

### A high-throughput parental RNAi screen for early-life determinants of proteostasis in late life

**Figure EV1:**
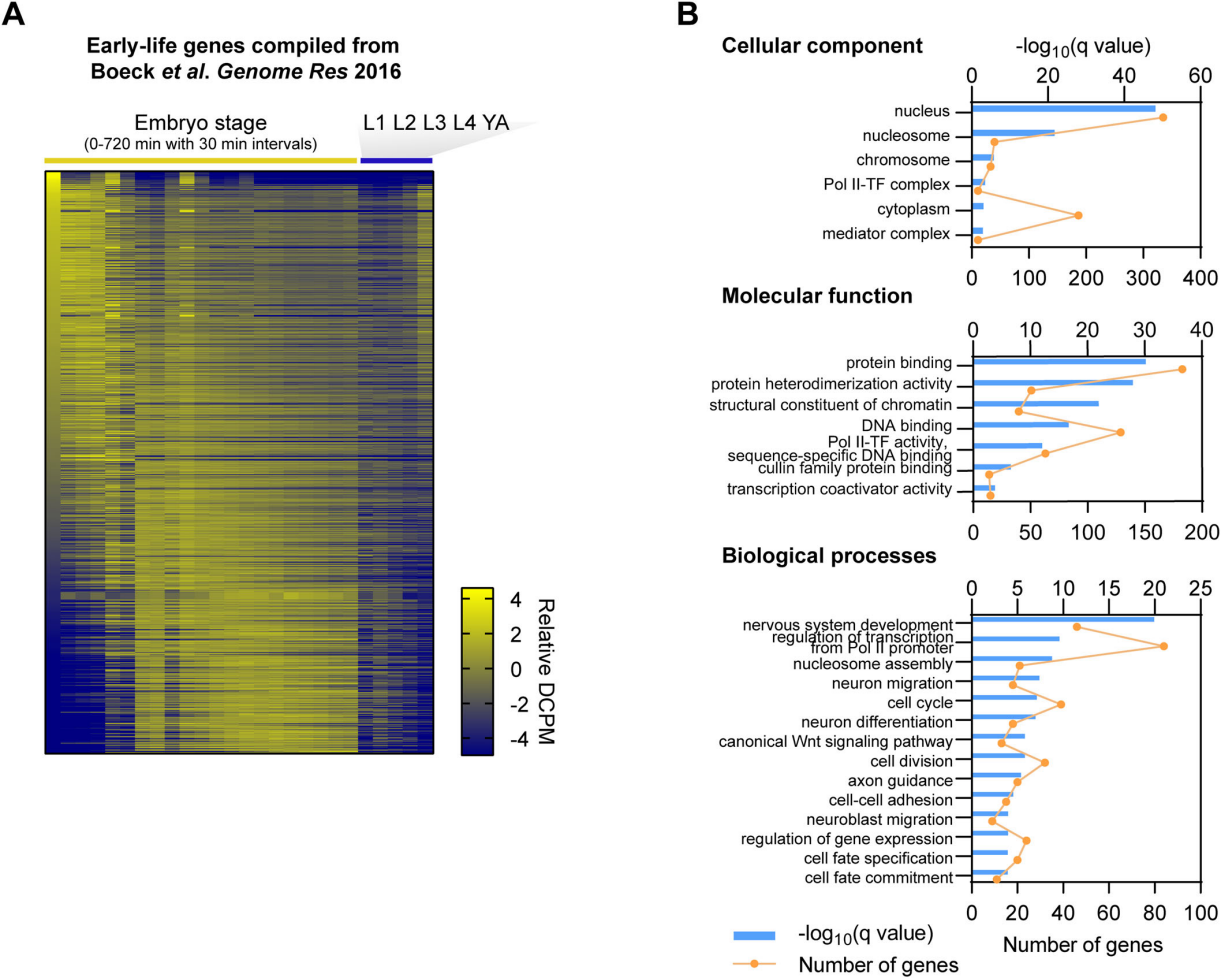
Expression pattern and functional analysis of genes selected for early-life RNAi screen. Related to Fig. 1. (**A**) Heatmap of relative expression levels of the early-life genes. (**B**) GO enrichment analysis of the early-life genes. Source data are available online for this figure.

We hypothesized that genes highly or exclusively expressed during early development are likely to function as master regulators of developmental priming events. Using published RNA-seq datasets (Boeck et al, 2016), we curated a list of 1059 candidate genes that are highly expressed in embryonic stages, hereafter referred to as early-life genes (Fig. EV1A; Dataset EV1). These early-life genes predominantly encode nuclear proteins involved in transcriptional regulation, chromatin remodeling, and cell cycle control (Fig. EV1B), implicating their roles in modulating biological processes through direct gene expression control or lifelong programming of gene activities.

**Figure 1:**
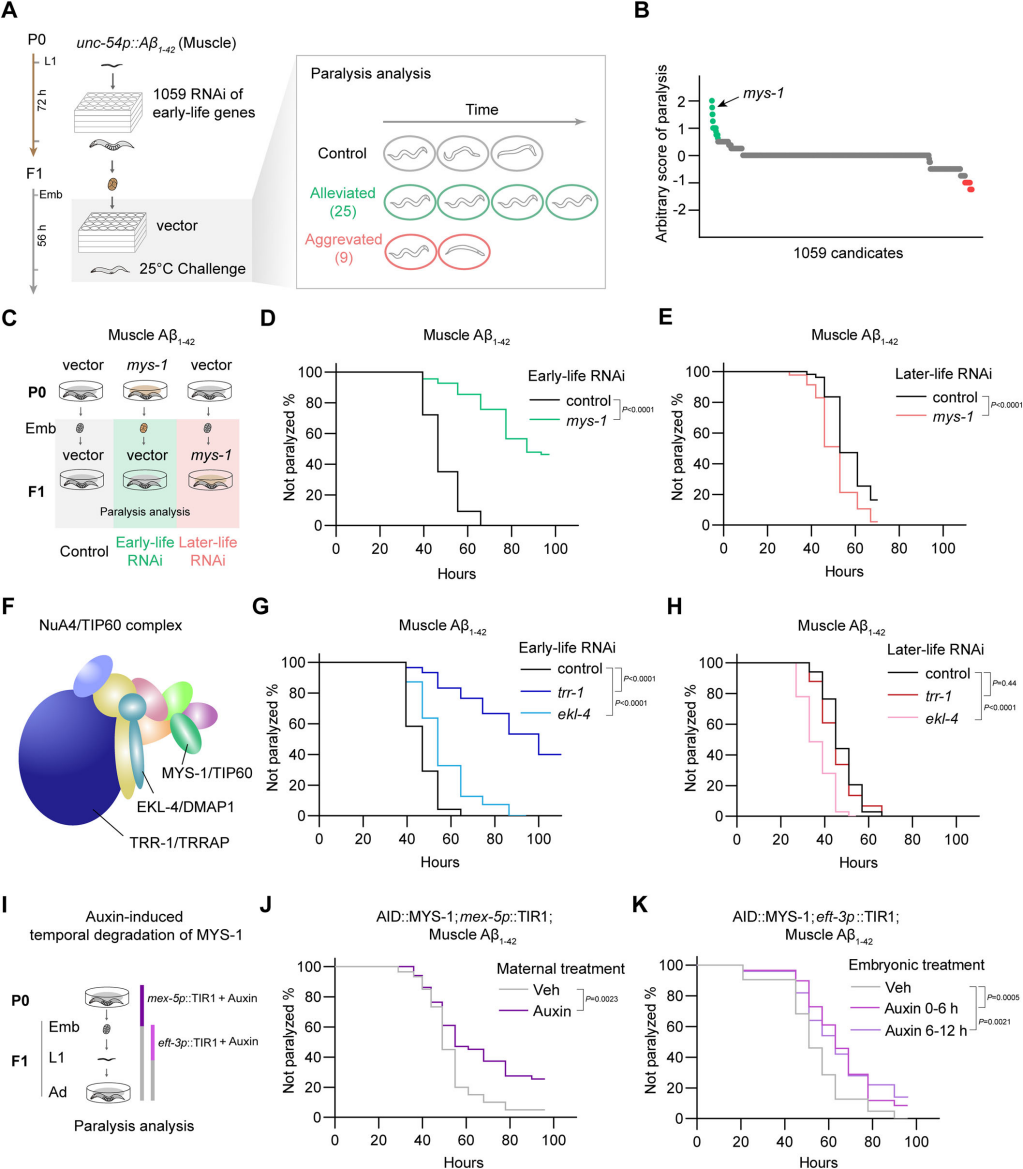
An early-life RNAi screen identifies NuA4 as a developmental regulator of late-life proteostasis. (**A**) Scheme of the parental RNAi screen for early-life genes that modulate adulthood susceptibility to muscle Aβ_1–42_-induced paralysis. (**B**) The arbitrary paralysis scores of early-life RNAi candidates. (**C**) Experimental illustration for early-life or later-life *mys-1* RNAi treatment. (**D**,** E**) Paralysis analysis of animals subjected to early-life (**D**) or later-life (**E**) *mys-1* RNAi. (**F**) Illustration of the NuA4 complex. (**G**,** H**) Paralysis analysis of animals subjected to *trr-1* and *ekl-4* RNAi in early life (**G**) or later life (**H**). (**I**) Illustration of the AID experiments for the temporal removal of MYS-1 in early life. (**J**) Paralysis analysis of the AID AD model animals maternally treated with 4 mM K-NAA. (**K**) Paralysis analysis of the AID AD model animals at the indicated embryonic stages following treatments with 1 mM IAA-AM. Results are representative of three independent experiments, and statistical significance was assessed by the log-rank test (**D**, **E**, **G**, **H**, **J**, **K**). Source data are available online for this figure.

To determine whether early-life genes program proteostasis in late life, we performed an RNAi screen targeting these genes using an established *C. elegans* AD model (McColl et al, 2012). This model constitutively expresses human Aβ 1–42 peptide in muscle and develops progressive paralysis when shifted from 20 to 25 °C. We employed parental RNAi via feeding to deliver dsRNA through oocytes to F1 progeny (hereafter termed early-life RNAi). Adult F1 animals subjected to early-life RNAi were then assayed for susceptibility to Aβ-induced paralysis (Fig. 1A; Dataset EV1). Notably, across two blind, independent replicates, RNAi knockdown of 25 genes conferred resistance to late-life proteotoxicity, while knockdown of nine genes increased susceptibility relative to controls (Fig. 1A,B; Dataset EV1). These results establish an early-life genetic basis for lifelong proteostasis maintenance.

### Temporal suppression of NuA4 activity in early life enhances late-life proteostasis and extends lifespan

**Figure EV2:**
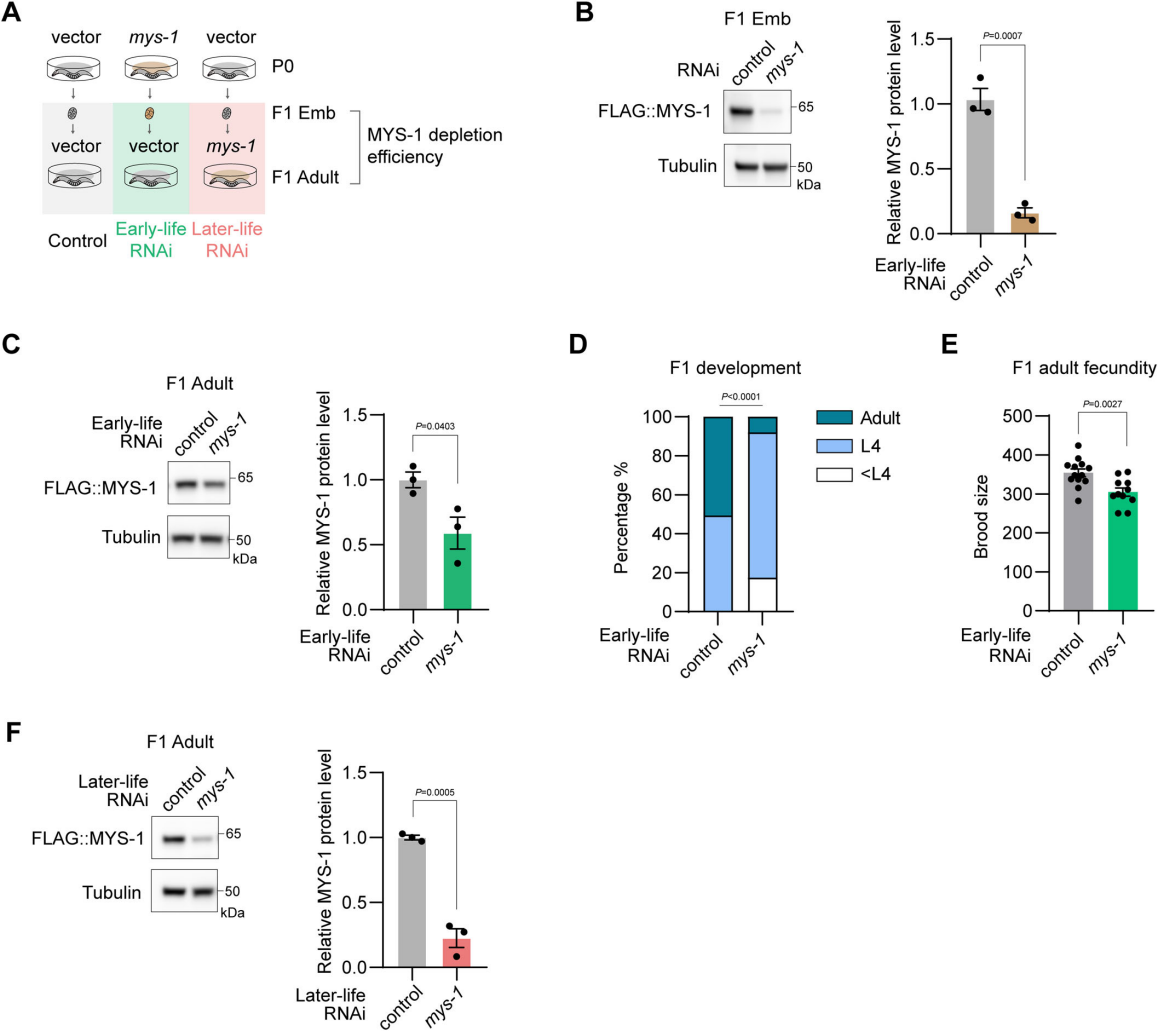
Detection of MYS-1 protein levels following early- and later-life RNAi treatments. Related to Fig. 1. (**A**) Experimental illustration for assessing MYS-1 depletion efficiency following early-life or later-life *mys-1* RNAi. (**B**,** C**) Western blot analysis of MYS-1 protein levels in F1 animals in embryonic (**B**) and adulthood stages (**C**) following early-life *mys-1* RNAi treatment. Results are average  ± SEM of three independent experiments. Statistical significance was assessed by a two-tailed unpaired *t*-test. (**D**) Developmental stage quantification under early-life *mys-1* RNAi. Results are a composite of three independent experiments. Animal number = 217 (control), 126 (*mys-1*). Statistical significance was assessed by the chi-squared test. (**E**) Brood size quantification under early-life *mys-1* RNAi. Results are representative of three independent experiments. Data were average ± SEM. Animal number = 13 (control), 11 (*mys-1*). Statistical significance was assessed by a two-tailed unpaired *t*-test. (**F**) Western blot analysis of MYS-1 protein levels in F1 adults following later-life *mys-1* RNAi treatment. Results are average ± SEM of three independent experiments. Statistical significance was assessed by a two-tailed unpaired *t*-test. Source data are available online for this figure.

Among top resistance candidates, early-life knockdown of the acetyltransferase gene *mys-1* (Fig. EV2A–C) markedly attenuated Aβ-induced paralysis in late life (Fig. 1C,D), despite causing developmental delay and reduced fecundity (Fig. EV2D,E). Conversely, post-embryonic RNAi (hereafter referred to as later-life RNAi), which achieved efficient adult-stage knockdown (Fig. EV2F), exacerbated paralysis relative to vector controls (Fig. 1C,E). To determine whether MYS-1, the *C. elegans* ortholog of human TIP60 histone acetyltransferase (HAT) in the TIP60/NuA4 HAT complex (Ceol and Horvitz, 2004), acts through the complex activity, we targeted two additional NuA4 components: *trr-1/TRRAP* and *ekl-4/DMAP1*. Early-life knockdown of either gene similarly conferred protection against late-life Aβ proteotoxicity, while later-life knockdown did not (Fig. 1F–H), collectively demonstrating that NuA4 complex activity during development mediates lifelong proteostasis regulation.

**Figure EV3:**
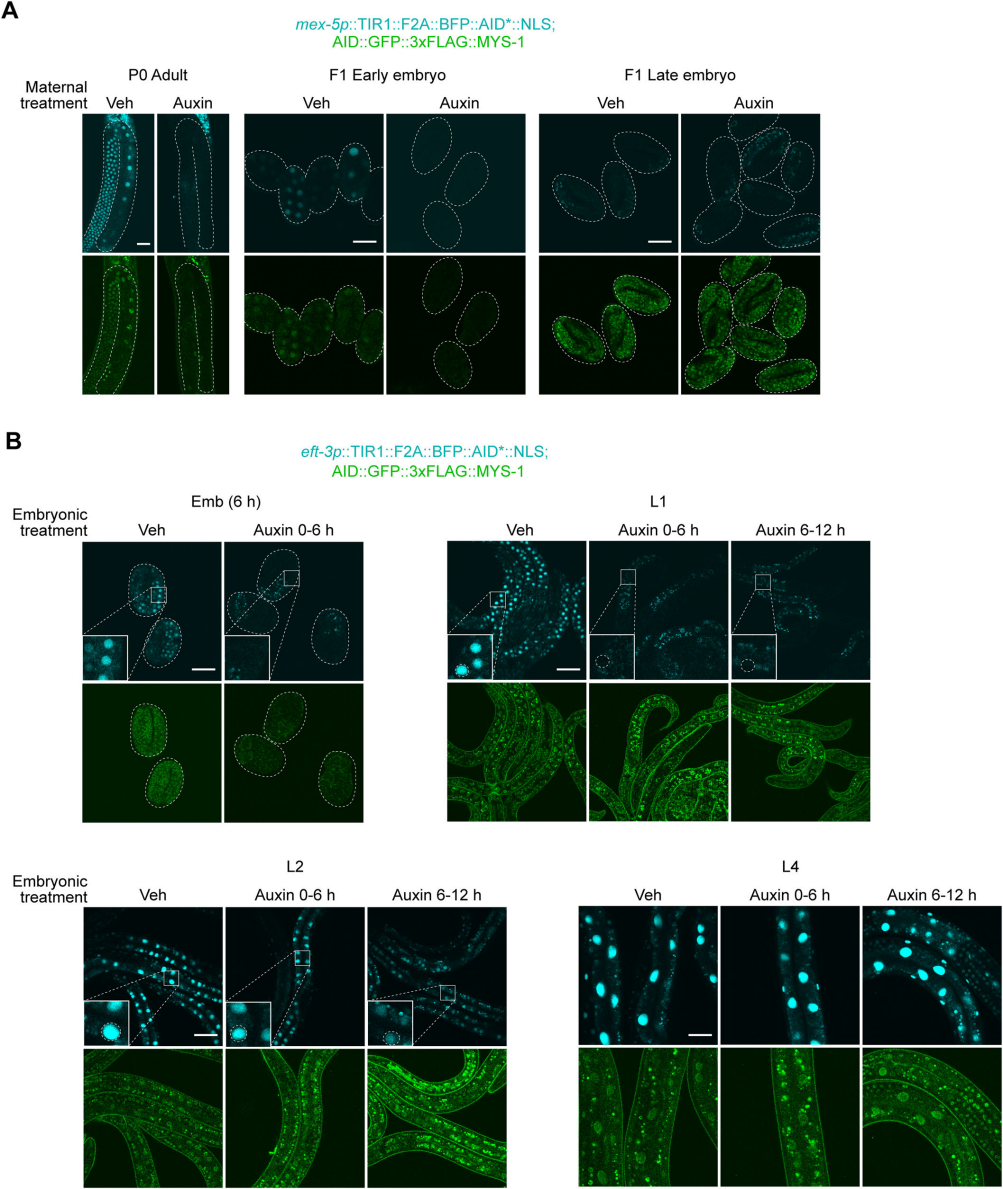
Validation of temporal removal of MYS-1 in AID systems. Related to Fig. 1. (**A**) Representative images of *mex-5p*::BFP and GFP::MYS-1 expression in animals at the indicated stages after maternal auxin treatment (K-NAA 4 mM). Scale bar: 20 μm. (**B**) Representative images of *eft-3p*::BFP and GFP::MYS-1 expression in animals at the indicated stages after embryonic auxin treatment (IAA-AM 1 mM). Scale bar: 20 μm. Results are representative of three independent experiments. Animal number >20 per group per experiment. Source data are available online for this figure.

To validate the RNAi results and determine the critical time window for early-life NuA4 activity in programming lifelong proteostasis, we implemented an auxin-inducible degradation (AID) system for temporal control of MYS-1 levels. This system integrated a fused AID tag to endogenous MYS-1 and substrate recognizer TIR1 under control of a tissue-specific promoter, enabling spatiotemporal protein degradation in the presence of auxin (Ashley et al, 2021). Using the germline-specific *mex-5* promoter (Li et al, 2022), auxin treatment in the parental worms depleted MYS-1 in germlines and early embryos, with recovery in late embryos (Figs. 1I and EV3A). Within the ubiquitous *eft-3p* driver system (Zhang et al, 2015), MYS-1 was efficiently depleted by early-life auxin treatments in embryos and L1 larvae but largely restored by the L2 stage (Figs. 1I and EV3B). Crucially, temporal loss of MYS-1 during early life, before the ~L2 stage, through either system conferred significant protection against late-life proteotoxicity (Fig. 1J,K), establishing the early-life priming role of the NuA4 complex in lifelong proteostasis regulation.

**Figure EV4:**
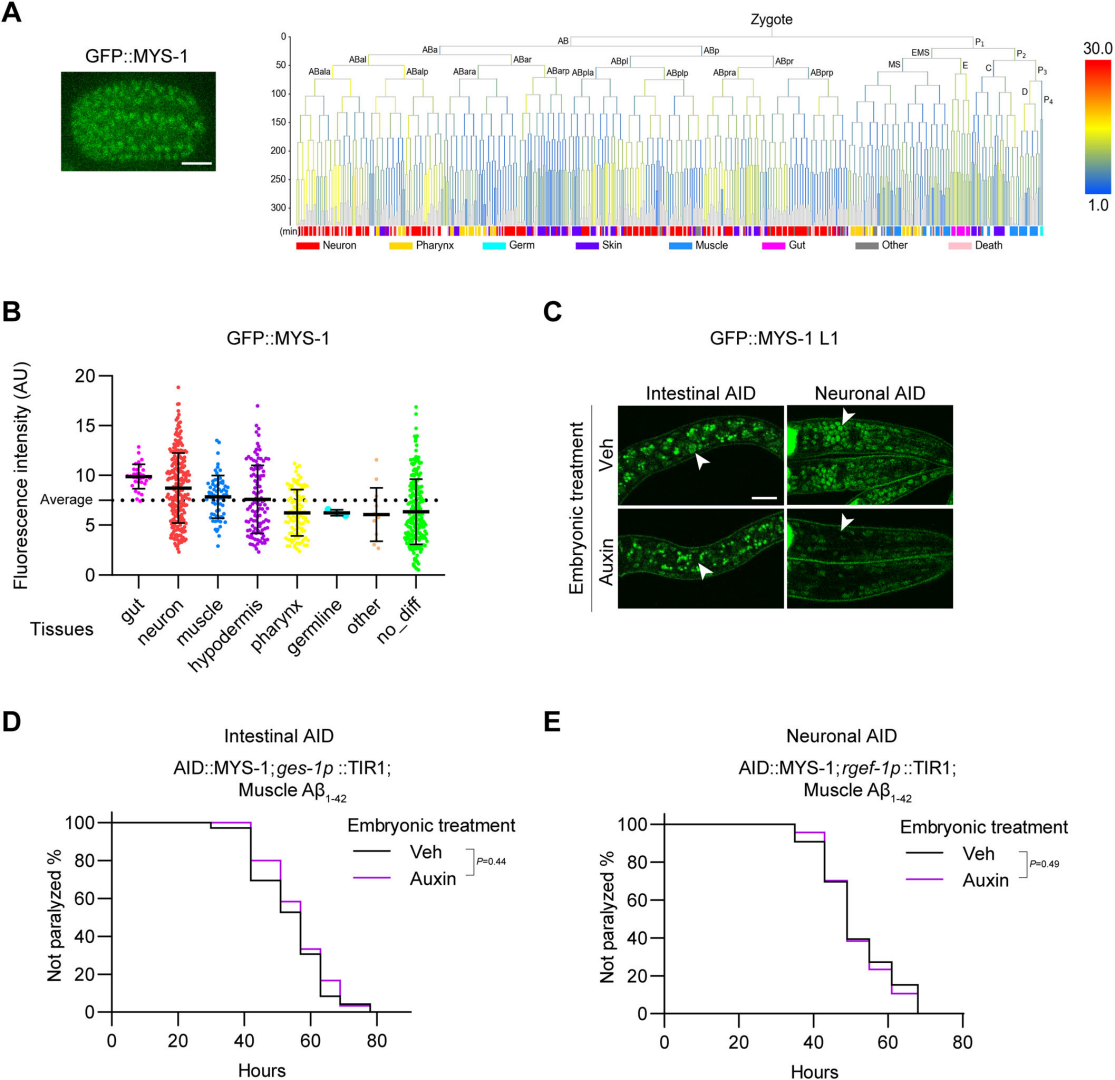
Tissue-specificity analysis of early-life MYS-1 expression and function. Related to Fig. 1. (**A**) Representative image of GFP::MYS-1 in embryos (left) and tree visualization of MYS-1 expression mapped on the embryonic cell lineage (right). Scale bar: 10 μm. (**B**) Quantification of MYS-1 expression in the indicated tissue type. Data were average ± SD. Each dot represents one cell. Results are representative of five embryos traced and analyzed, yielding similar results. (**C**) Representative image of GFP::MYS-1 L1 animals with intestine and neuron-specific degradation in the embryo-to-L1 stage. Arrowheads indicate the GFP::MYS-1 signal in the nucleus of intestine cells and head neurons. Scale bar: 10 μm. Results are representative of three independent experiments. Animal number >20 per group per experiment. (**D**, **E**) Paralysis analysis of the muscle Aβ_1–42_ animals with intestinal (**D**) and neuronal (**E**) specific AID in early life. Results are representative of three independent experiments. Statistical significance was assessed by the log-rank test. Source data are available online for this figure.

To investigate whether the lifelong regulatory function of MYS-1 is mediated by specific tissues, we first examined its expression pattern. Using a knock-in GFP::MYS-1 tool, we observed that MYS-1 protein is broadly expressed during embryogenesis, with quantifiably higher levels in gut and neuronal precursor cells (Fig. EV4A,B). We next asked whether depleting MYS-1 specifically in these tissues during the critical embryonic/L1 time window was sufficient to recapitulate the long-term protective phenotypes. By driving TIR with the intestine-specific *ges-1* promoter and the neuronal-specific *rgef-1* promoter, we induced acute protein degradation exclusively in the intestine or neurons at the L1 stage (Fig. EV4C). However, neither tissue-specific depletion reproduced the improvement in proteostasis (Fig. EV4D,E). These findings suggest that MYS-1 functions as a systemic regulator rather than a tissue-specific determinant in establishing adult health trajectories.

**Figure 2:**
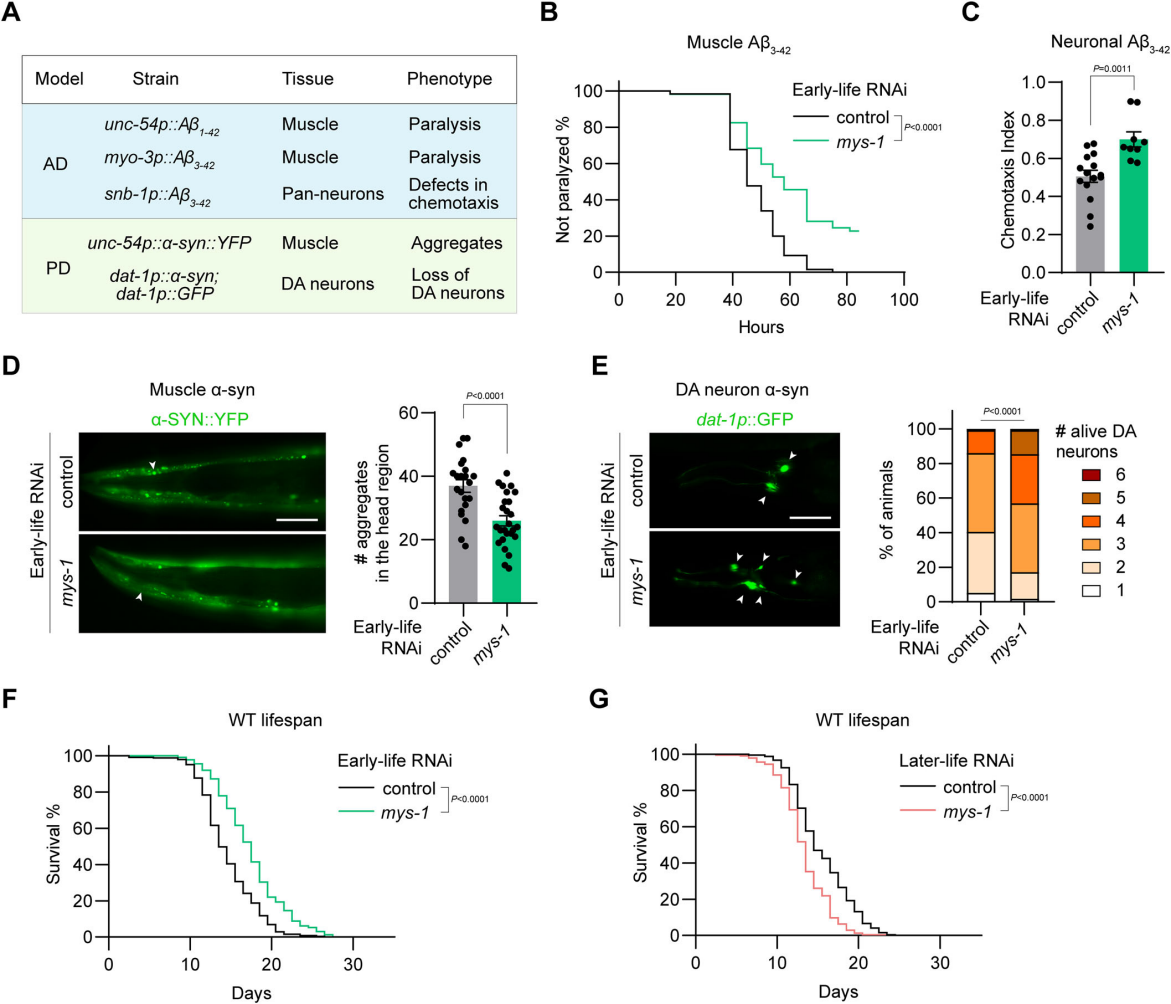
Early-life NuA4 programs lifelong systemic proteostasis and longevity. (**A**) Proteotoxicity models used in this study. (**B**) Paralysis analysis of muscle Aβ_3–42_ animals subjected to early-life *mys-1* RNAi. Results are representative of three independent experiments. Statistical significance was assessed by the log-rank test. (**C**) Chemotaxis assay for neuronal Aβ_3–42_ animals subjected to early-life *mys-1* RNAi. Results are average ± SEM of three independent experiments. Assay plate ≥3 per group per experiment, animal number >50 per assay plate. Statistical significance was assessed by a two-tailed unpaired *t*-test. (**D**) Representative images and quantifications of protein aggregates in Day 5-old adult animals expressing α-SYN::YFP in muscle subjected to early-life *mys-1* RNAi. The signal of α-SYN::YFP in the pharynx muscle was indicated by the white arrows. Scale bar: 50 μm. Data were average ± SEM of three independent experiments. Animal number = 22 (control) and 26 (*mys-1*). Statistical significance was assessed by a two-tailed unpaired *t*-test. (**E**) Representative images and quantifications of the number of alive head DA neurons in Day 5-old adult animals expressing α-SYN n in DA neurons subjected to early-life *mys-1* RNAi. DA neurons were indicated by white arrows. Scale bar: 50 μm. The data were a composite of three independent experiments. Animal number = 136 (control) and 116 (*mys-1*). Statistical significance was assessed by the chi-squared test. (**F**, **G**) The lifespans of WT (N2) animals subjected to early-life (**F**) or later-life (**G**) *mys-1* RNAi. Results are a composite of three independent experiments. Statistical significance was assessed by the log-rank test. Source data are available online for this figure.

**Figure EV5:**
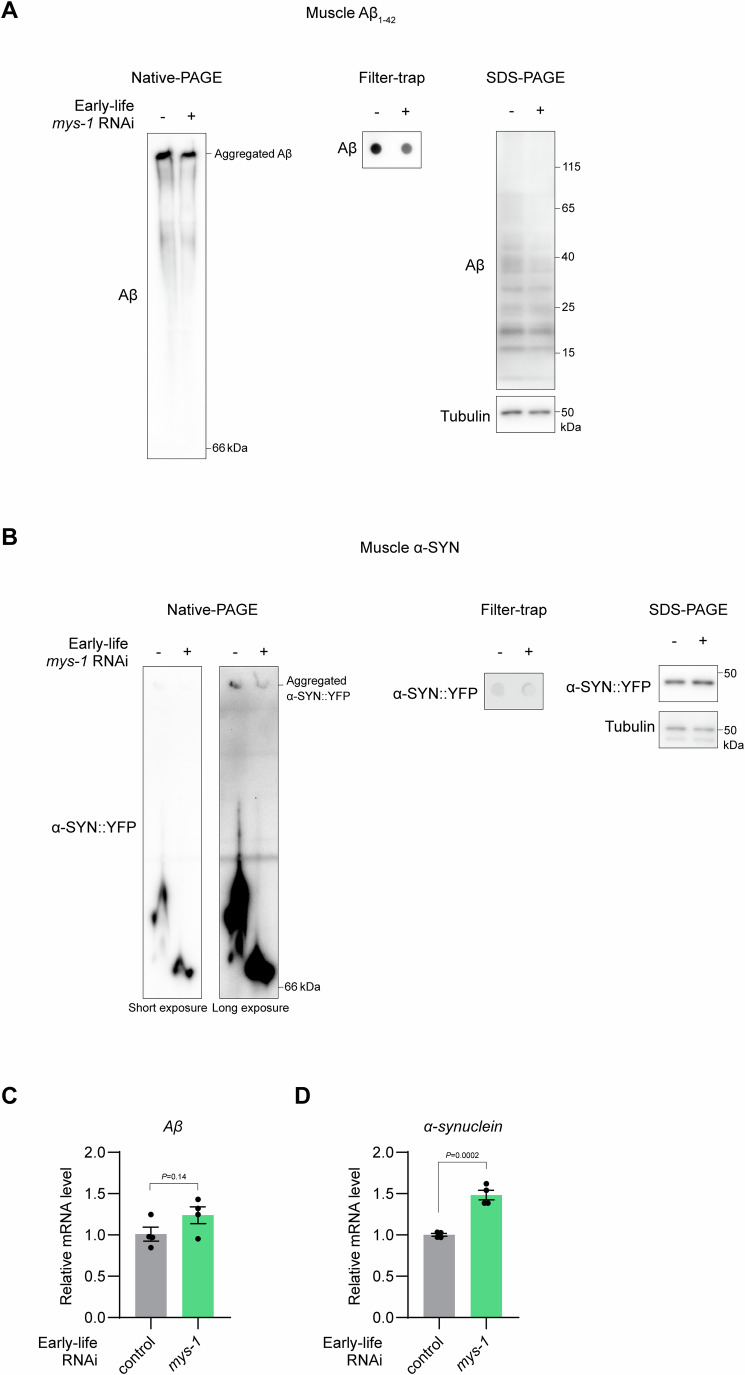
Early-life *mys-1* RNAi reduces protein aggregates in late life. Related to Fig. 2. **A**, **B** Protein aggregates analysis of Aβ and α-synuclein in adult animals under early-life *mys-1* RNAi with native Western blot, filter trap, and denaturing Western blot. Results are representative of three independent experiments. (**C**, **D**) Relative mRNA level of *Aβ* and *α-synuclein* in adult animals under early-life *mys-1* RNAi. Results are average ± SEM of three independent replicates. Statistical significance was assessed by a two-tailed unpaired *t*-test. Source data are available online for this figure.

To determine whether the effects of early-life NuA4 activity represent systemic regulation of proteostasis, rather than effects specific to muscle Aβ_1–42_ toxicity, we analyzed additional *C. elegans* proteotoxicity models (Fig. 2A): the AD models that express the human Aβ_3–42_ peptide in body wall muscle or pan-neuronally (Link, 1995; Luo et al, 2009), and PD models expressing human α-synuclein protein in muscle or dopaminergic (DA) neurons (van Ham et al, 2008; Cooper et al, 2006). Consistent with its effects in the initial screening, early-life *mys-1* RNAi conferred significant resistance against proteotoxicity induced by each pathological protein, whether expressed in muscle or neurons (Fig. 2B–E). To confirm that this protection corresponds to a genuine reduction in toxic protein species, we directly assessed the aggregation state of Aβ and α-SYN. Native gel Western blot and filter-trap assays revealed that early-life *mys-1* RNAi specifically reduced the abundance of high-molecular-weight, SDS-insoluble Aβ and α-SYN aggregates (Fig. EV5A,B). Critically, total protein levels of Aβ and α-SYN, as measured by denaturing Western blot (Fig. EV5A,B), and their corresponding mRNA levels remained unchanged (Fig. EV5C,D), ruling out nonspecific effects on expression. These results demonstrate that early-life NuA4 activity systemically regulates proteostasis by reducing the burden of toxic protein aggregates. Furthermore, early-life depletion of *mys-1* significantly extended lifespan (Fig. 2F), whereas later-life RNAi shortened it (Fig. 2G). Together, these findings demonstrate that inhibiting NuA4 complex activity specifically during early life enhances proteostasis and extends longevity in *C. elegans*.

### Early-life reduction of NuA4-mediated H4K16ac triggers compensatory UPR^ER^ signaling to program lifelong proteostasis resilience

**Figure 3:**
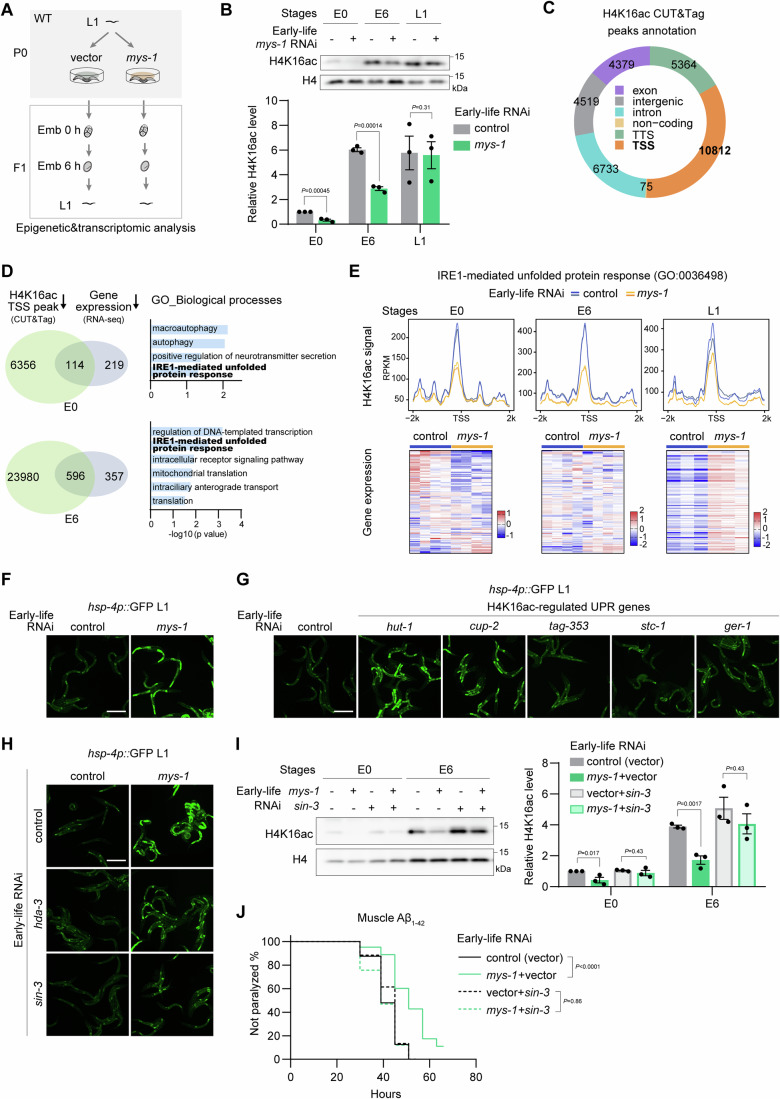
Early-life NuA4 fine-tunes H4K16ac for lifelong proteostasis programming. (**A**) Strategic design of the epigenomic and transcriptomic analysis in embryos immediately after collection (E0), embryos 6 h post-collection (E6) and L1 animals following early-life *mys-1* RNAi treatment. (**B**) Western blot analysis of H4K16ac in animals at the indicated stages following early-life *mys-1* RNAi treatment. Results are average ± SEM of three independent experiments. Statistical significance was assessed by a two-tailed unpaired *t*-test with FDR correction. (**C**) Distribution of H4K16ac CUT&Tag peaks in the genome. (**D**) Venn diagram (left) and GO enrichment analysis (right) of genes that were both downregulated in H4K16ac peaks (at their TSS region) and in transcription levels following early-life *mys-1* RNAi treatment. (**E**) Profiles of H4K16ac signals (Reads Per Kilobase per Million) at the TSS region (upper panel, each group contains two lines, representing two replicates) and heatmap of relative expression levels (lower panel) of the IRE1-UPR^ER^ genes (refer to GO:0036498) in animals at the indicated stages following early-life *mys-1* RNAi treatment. (**F**–**H**) Representative images of the *hsp-4p::*GFP animals in the L1 stage following early-life RNAi treatment targeting *mys-1* (**F**), H4K16ac-regulated UPR genes (**G**) and dually *mys-1* and *hda-3* or *sin-3* RNAi (**H**). Scale bar: 100 μm. Results are representative of three independent experiments. Animal number >20 per group per experiment. (**I**) Western blot analysis of H4K16ac in animals at the indicated stages following early-life RNAi treatment dually targeting *mys-1* and *sin-3*. Results are average ± SEM of three independent experiments. Statistical significance was assessed by a two-tailed unpaired *t*-test with FDR correction. (**J**) Paralysis analysis of muscle Aβ_1–42_ animals subjected to early-life RNAi dually targeting *mys-1* and *sin-3*. Results are representative of three independent experiments. Statistical significance was assessed by the log-rank test. Source data are available online for this figure.

**Figure EV6:**
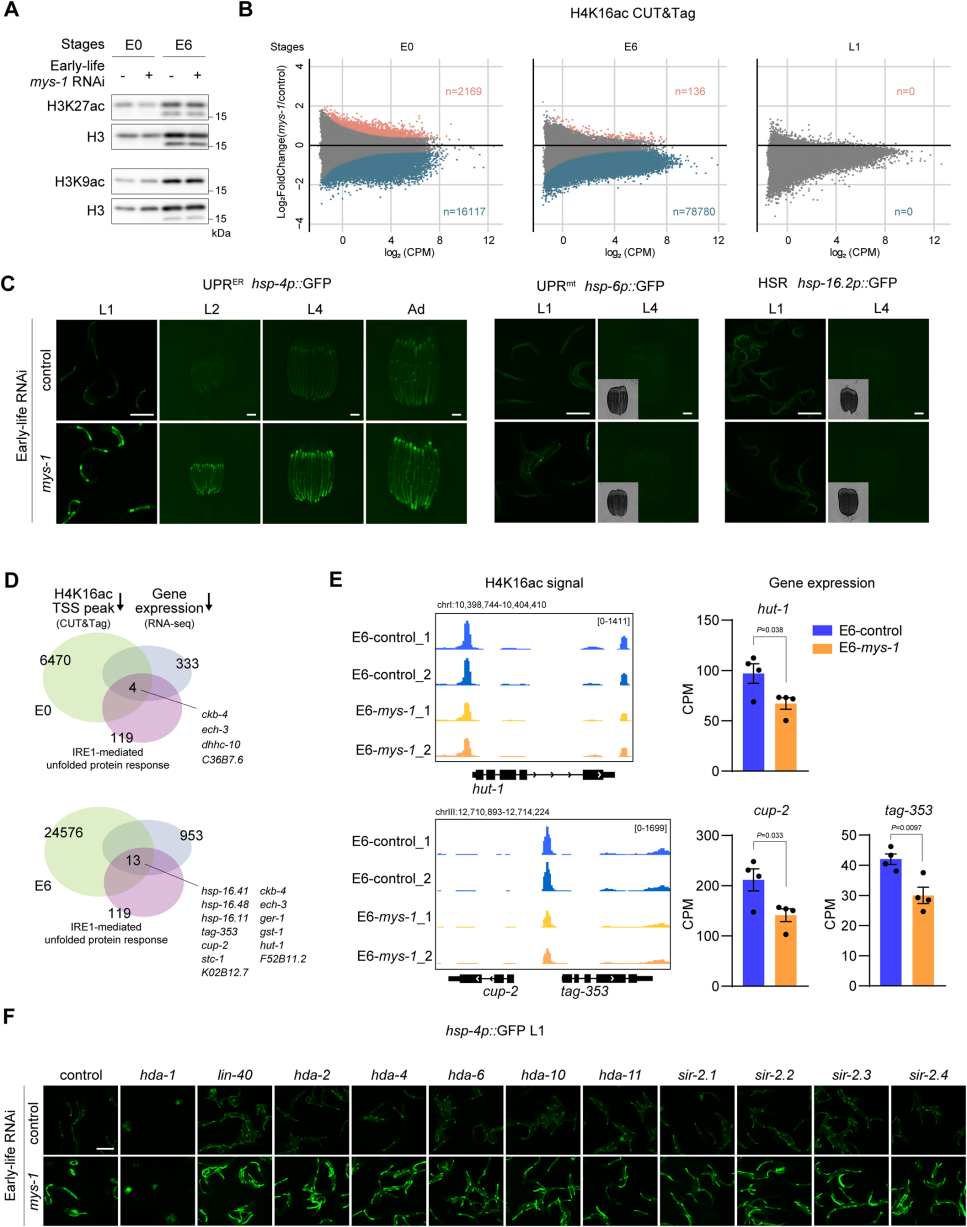
Epigenomic and transcriptomic analysis of genes affected by early-life *mys-1* RNAi. Relate to Fig. 3. (**A**) Western blot analysis of H3K9ac and H3K27ac levels in animals at the indicated stages following early-life *mys-1* RNAi treatment. Results are representative of three independent experiments. (**B**) Volcano plot of differential H4K16ac peaks at indicated stages following early-life *mys-1* RNAi treatment. (**C**) Representative images of the *hsp-4p::*GFP, *hsp-6p::*GFP and *hsp-16.2p::*GFP animals in the indicated stage following early-life RNAi of *mys-1*. Scale bar: 100 μm. Results are representative of three independent experiments. Animal number >20 (for L1) or 10 (for L2, L4, and Ad) per group per experiment. Note: The representative control image (L1) is the same as that shown in Fig. 5C, as both experiments utilized identical control conditions. (**D**) Venn diagram of the IRE1-UPR^ER^ pathway genes that were downregulated on both H4K16ac peaks (at TSS regions) and transcription levels following early-life *mys-1* RNAi treatment. (**E**) The IGV tracks of H4K16ac CUT&Tag analysis (left, two independent replicates) and RNA-seq signals (right, four independent replicates) of the representative genes from (**D**). Data were average ± SEM. Statistical significance was assessed by a two-tailed unpaired *t*-test. (**F**) Representative images of the *hsp-4p::*GFP animals in the L1 stage following early-life RNAi dually targeting *mys-1* and HDAC genes. Scale bar: 100 μm. Results are representative of two independent experiments. Animal number >20 per group per experiment. Source data are available online for this figure.

To investigate the mechanisms of NuA4-mediated lifelong proteostasis programming established early in life, we collected animals at three early-life stages for epigenomic and transcriptomic profiling: (i) embryos immediately after bleach isolation (E0, proliferation stage), (ii) embryos at 6 h post-isolation (E6, morphogenesis stage(Hall et al, 2017)), and (iii) L1-stage larvae (L1) (Fig. 3A). The epigenetic analysis was designed in accordance with previous studies identifying H4K16ac as the key acetylation target of MYS-1 in *C. elegans* (Lau et al, 2016; Ikeda et al, 2017). Consistent with these reports, we observed that early-life *mys-1* RNAi significantly reduced global H4K16ac level during embryonic stages, but it was nearly fully restored by the L1 stage (Fig. 3B). This effect was specific to H4K16ac, as the levels of other active histone marks, H3K9ac and H3K27ac, remained unchanged (Fig. EV6A). Furthermore, Cleavage Under Targets and Tagmentation (CUT&Tag) analysis showed that early-life *mys-1* RNAi globally reduced the H4K16ac signal in the genome, particularly in embryos (Fig. EV6B; Dataset EV2). Intriguingly, H4K16ac exhibited marked enrichment at transcription start site (TSS) regions (Fig. 3C), aligning with its established function in facilitating transcription (Samata et al, 2020; Liu et al, 2011).

Given the significant reduction in H4K16ac signals following early-life *mys-1* RNAi, we focused our analysis on genes showing concomitant reductions in both H4K16ac binding and gene expression in the early embryos, which were considered as direct targets of the NuA4-H4K16ac. Notably, the IRE1-mediated UPR^ER^ signaling was consistently enriched among the downregulated genes in both embryonic stages analyzed (Fig. 3D; Dataset EV3). This pathway is known to play critical roles in maintaining ER homeostasis, including protein-folding, transport, and degradation (Hetz, 2012). We therefore performed a focused analysis of H4K16ac binding and target gene expression specifically within the IRE1-UPR^ER^ pathway (Fig. 3E). Strikingly, UPR^ER^ pathway genes exhibited prominent H4K16ac peaks at their TSS regions. Early-life *mys-1* RNAi induced correlated reductions in H4K16ac signals and expression of UPR^ER^ pathway genes in early embryos (E0), with partial recovery in late embryos (E6). Unprecedently, however, despite unchanged H4K16ac levels relative to controls, the entire IRE1-UPR pathway was robustly activated in the L1 stage (Fig. 3E). Consistent with these findings, early-life *mys-1* RNAi triggered a significant and sustained induction of the UPR^ER^ reporter (*hsp-4p::GFP*) in L1 that persisted into adulthood (Fig. EV6C). In contrast, the mitochondrial UPR (UPR^mt^) reporter *hsp-6p::GFP* exhibited only a mild, transient induction in the L1 stage, and the cytosolic heat shock response (HSR) reporter *hsp-16.2p::GFP* showed no detectable activation (Fig. EV6C). These results indicate that early-life NuA4 perturbation triggers a specific and sustained activation of the UPR^ER^, distinguishing it from a general cellular stress response.

To determine whether the L1-stage UPR^ER^ activation following embryonic suppression represents a compensatory upregulation and to investigate its mechanistic link to the early-life NuA4-H4K16ac axis, we used RNAi to knockdown of key direct NuA4-H4K16ac target genes (Fig. EV6D,E) and found many of them phenocopied the L1-stage UPR^ER^ activation under *mys-1* RNAi (Fig. 3F,G). These genes included *hut-1*, the ortholog of UDP-Gal transporter localized to the ER, and *cup-2*, the ortholog of Derlin, an essential component for ER-associated degradation, inactivation of which is known to induce ER stress (Dejima et al, 2009; Ye et al, 2004). Based on these findings, we propose that reduced NuA4-H4K16ac activity downregulates these UPR-related genes during embryogenesis, inducing ER stress that triggers compensatory upregulation of the UPR^ER^ pathway in L1 larvae.

To further validate the epistatic relationship between H4K16ac loss and UPR^ER^-activation, we asked whether increasing histone acetylation by inhibiting histone deacetylase (HDAC) activity could counteract the H4K16ac depletion caused by NuA4 perturbation. Indeed, among multiple histone deacetylase complex (HDACs) classes screened, RNAi targeting *hda-3* and *sin-3*, which were recently identified in the same HDAC complex (Robert et al, 2023), both markedly diminished the effect of early-life *mys-1* RNAi-induced UPR^ER^ activation in L1 animals (Figs. 3H and EV6F). Furthermore, *sin-3* knockdown significantly restored H4K16ac levels in early-life *mys-1* RNAi-treated embryos (Fig. 3I). Crucially, *sin-3* knockdown abolished the beneficial effects of early-life *mys-1* RNAi on late-life proteostasis regulation (Fig. 3J). Collectively, our results demonstrate that epigenetic priming of H4K16ac by the NuA4 complex during early life fine-tunes UPR^ER^ signaling to mediate lifelong proteostasis maintenance.

### XBP-1–mediated UPR^ER^ links early-life NuA4-H4K16ac signaling to proteostatic regulation in late life

**Figure EV7:**
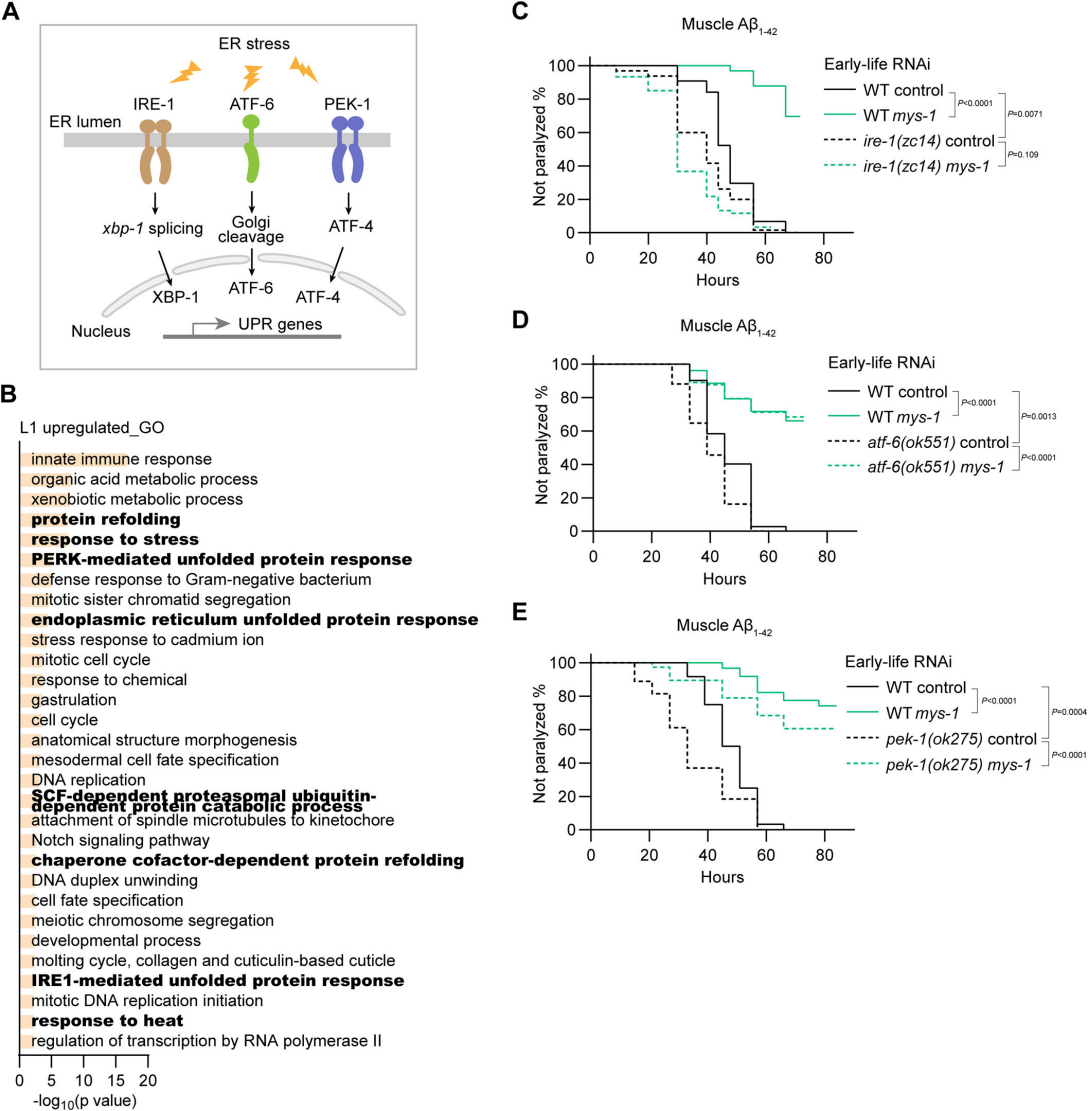
Early-life NuA4 programs lifelong proteostasis exclusively through the IRE-1–XBP-1 pathway. Related to Fig. 4. (**A**) Schematic diagram of main ER stress pathways. (**B**) GO enrichment analysis of upregulated genes in L1 animals subjected to early-life *mys-1* RNAi. Terms related to UPR^ER^ are shown in bold. (**C**–**E**) Paralysis analysis of muscle Aβ_1–42_ animals in WT and *ire-1(zc14)* (**C**), *atf-6(ok551)* (**D**), and *pek-1(ok275)* (**E**) genetic background, following early-life *mys-1* RNAi treatment. Results are representative of three independent experiments. Statistical significance was assessed by the log-rank test. Source data are available online for this figure.

**Figure 4:**
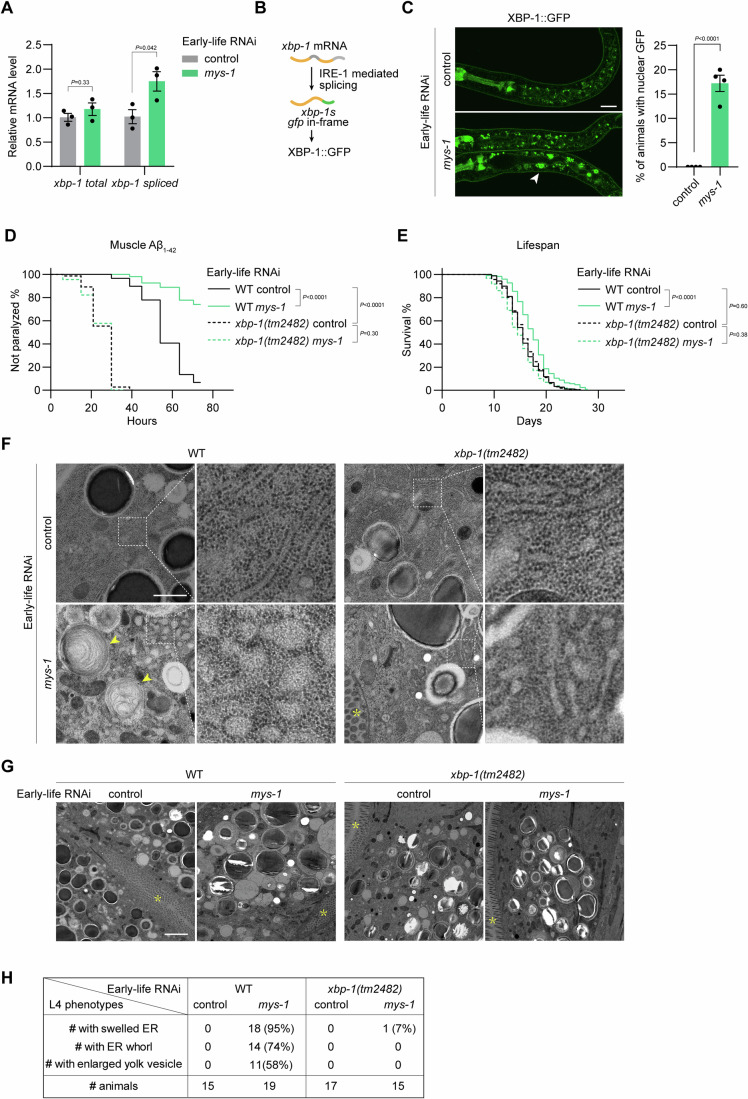
XBP-1 is essential for early-life NuA4 activity to program late-life outcomes. (**A**) Relative mRNA levels of the spliced isoform of *xbp-1* and total *xbp-1* in L1 animals. Results are average ± SEM of three independent replicates. Statistical significance was assessed by a two-tailed unpaired *t*-test. (**B**) Schematic illustration of the *xbp-1* splicing reporter. (**C**) Representative images and quantifications of the active XBP-1::GFP in L1 animals under early-life treatment of *mys-1*. White arrows indicate the XBP-1::GFP signal in the nucleus. Scale bar: 10 μm. Results are average ± SEM of three independent experiments. Animal number >20 per group per replicate. Statistical significance was assessed by a two-tailed unpaired *t*-test. (**D**, **E**) Paralysis analysis of muscle Aβ_1–42_ animals (**D**) and lifespan (**E**) of WT and *xbp-1(tm2482)* following early-life *mys-1* RNAi treatment. Results are representative of three independent experiments. Statistical significance was assessed by the log-rank test. (**F**–**H**) Representative TEM images (**F**, **G**) and phenotypic quantification (**H**) of the intestinal regions of WT and *xbp-1(tm2482)* animals in the L4 stage following early-life *mys-1* RNAi treatment. The intestine lumen was indicated by an asterisk (**F**, **G**); The enlarged ER structure was highlighted with a dotted frame (**F**); the ER whorls were indicated by yellow arrows (**F**). Scale bar: 1 μm (**F**) or 2 μm (**G**). Source data are available online for this figure.

Canonically, the UPR^ER^ signaling comprises three conserved branches: IRE-1, PEK-1 and ATF-6, each maintaining distinct aspects of ER proteostasis (Fig. EV7A). Activation of any of UPR^ER^ branches can protect against age-dependent proteotoxicity (Taylor and Dillin, 2013; Waldherr et al, 2019; Grandjean et al, 2020; Blackwood et al, 2019; Shin et al, 2021; Wang et al, 2022). Our transcriptomic analysis revealed that early-life *mys-1* RNAi activated multiple UPR^ER^ branches, including IRE-1 signaling, in L1 animals (Figs. 3E and EV7B). To determine which branch(es) mediate the late-life proteostasis benefits, we systematically disrupted each pathway. Strikingly, only loss-of-function mutation of *ire-1* (Hou et al, 2014) abolished the protective effects of early-life *mys-1* RNAi, while loss-of-function mutations in *atf-6* and *pek-1* (Burkewitz et al, 2020) had no effect (Fig. EV7C–E). IRE-1 primarily signals through splicing of *xbp-1* mRNA to generate the active transcription factor XBP-1 (Calfon et al, 2002). We therefore asked if *mys-1* depletion enhances this key splicing event. Quantitative PCR analysis confirmed a significant increase in the spliced *xbp-1s* isoform following *mys-1* RNAi, while total *xbp-1* mRNA levels remained unchanged (Fig. 4A). Consistently, using a translational *xbp-1* splicing reporter, we observed a marked increase in nuclear XBP-1::GFP signal in a subset of L1 animals (Fig. 4B,C), providing direct evidence for elevated levels of the active transcription factor. We next investigated the role of XBP-1 in the developmental programming of late-life proteostasis. Notably, the effects of early-life *mys-1* RNAi on both proteostasis enhancement in late life and lifespan extension were completely abolished in *xbp-1* loss-of-function mutants(Richardson et al, 2010) (Fig. 4D,E), demonstrating XBP-1 as an essential downstream effector of early-life NuA4 activity.

Intriguingly, our transmission electron microscope (TEM) analysis in the L4 animals exhibited that early-life *mys-1* RNAi induced significant abnormalities in ER morphology and yolk-associated vesicle size (Fig. 4F–H), characterized by swollen, circular ER structures with multilayered concentric ER whorls (Fig. 4F) and enlarged yolk-associated vesicles (Fig. 4G). These alterations are established hallmarks of ER stress, ER-phagy conditions (Schäfer et al, 2020; Schuck et al, 2014; Schuck et al, 2009; Xu et al, 2021), and ectopic expression of the spliced, active form of XBP-1 (Daniele et al, 2020; Metcalf et al, 2024). Remarkably, *xbp-1* mutation almost completely reversed these morphological changes induced by early-life *mys-1* RNAi (Fig. 4F–H). Collectively, these findings establish the IRE-1-XBP-1-UPR^ER^ axis as the exclusive conduit linking early-life NuA4-H4K16ac activity to lifelong proteostasis regulation.

### Developmental XBP-1 activity is required for late-life proteostasis programming

**Figure 5:**
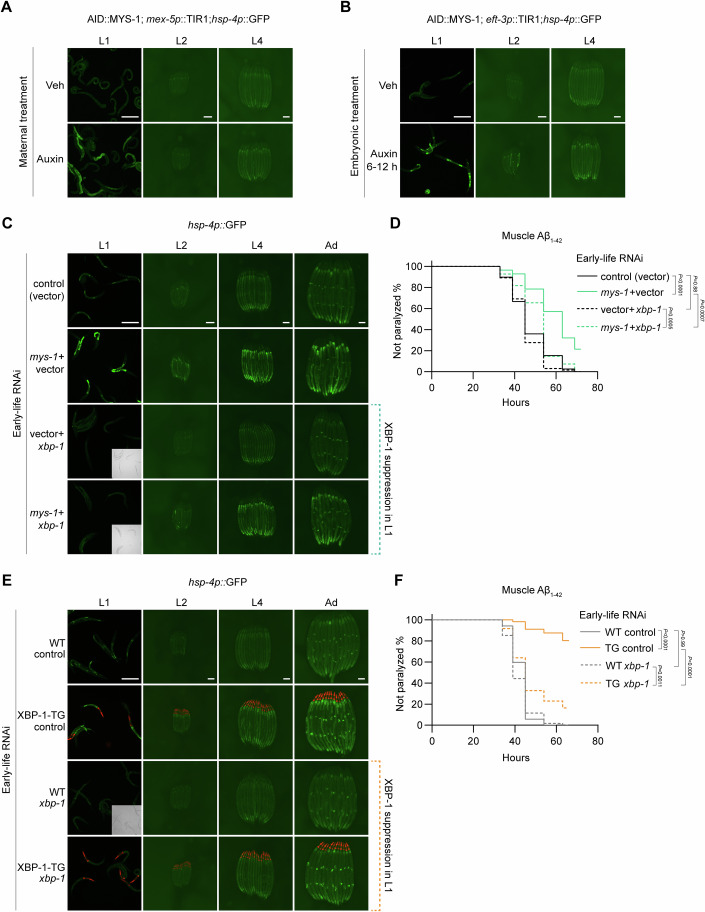
Developmental XBP-1 activity is required for early-life NuA4-mediated lifelong proteostasis programming. (**A**, **B**) Representative images of the *hsp-4p::*GFP animals at the indicated stages following maternal (**A**) or embryonic (**B**) removal of MYS-1 in the indicated AID animals. Scale bar: 100 μm. Results are representative of three independent experiments. Animal number >20 (for L1) or 15 (for L2 and L4) per group per experiment. (**C**) Representative images of the *hsp-4p::*GFP animals at the indicated stages following early-life RNAi treatment dually targeting *mys-1* and *xbp-1*. Scale bar: 100 μm. Results are representative of three independent experiments. Animal number >20 (for L1) or 10 (for L2, L4, and Ad) per group per experiment. Note: The representative control image (L1) is the same as that shown in Fig. EV6C, as both experiments utilized identical control conditions. (**D**) Paralysis analysis of muscle Aβ_1–42_ animals following early-life RNAi treatment dually targeting *mys-1* and *xbp-1*. Results are representative of four independent experiments. Statistical significance was assessed by the log-rank test. (**E**) Representative images of the *hsp-4p::*GFP animals in the indicated stages and genetic backgrounds following early-life *xbp-1* RNAi treatment. Scale bar: 100 μm. Results are representative of three independent experiments. Animal number >20 (for L1) or 10 (for L2, L4, and Ad) per group per experiment. (**F**) Paralysis analysis of muscle Aβ_1–42_ animals in indicated backgrounds following early-life *xbp-1* RNAi treatment. Results are representative of three independent experiments. Statistical significance was assessed by the log-rank test. Source data are available online for this figure.

**Figure EV8:**
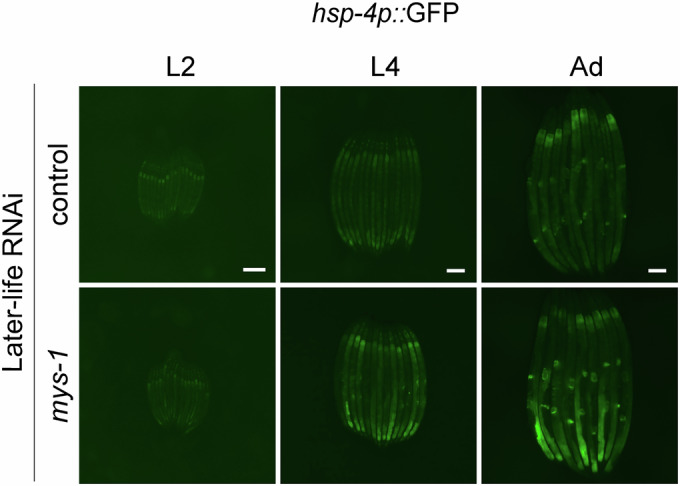
Later-life *mys-1* RNAi induces an unchanged early larval UPR^ER^ despite an increase in later stages. Related to Fig. 5. Representative images of *hsp-4p::*GFP animals at the indicated stages following later-life *mys-1* RNAi treatment. Scale bar: 100 μm. Results are representative of three independent experiments. Animal number >10 per group per experiment. Source data are available online for this figure.

Given the critical time window for the NuA4-H4K16ac axis to program lifelong proteostasis, we hypothesized that the timing of XBP-1-mediated UPR^ER^ signaling might also be essential for transducing these effects. Intriguingly, early-life depletion of MYS-1 using the AID system induced marked UPR^ER^ activation in the L1 stage, which gradually diminished in later stages, as indicated by the *hsp-4p::*GFP reporter (Fig. 5A,B). Moreover, early-life *mys-1* RNAi elicited prolonged UPR^ER^ activation throughout development (Fig. 5C), whereas later-life *mys-1* RNAi significantly activated UPR^ER^ solely after the L2 stage (Fig. EV8). Considering the contrasting late-life outcomes resulting from early-life versus later-life suppression of NuA4 activity (Fig. 1C–K), we speculated that early-life activation of XBP-1-mediated UPR^ER^ signaling is crucial for programming late-life health benefits. Indeed, suppressing *xbp-1* via RNAi during early life abolished both the L1-stage UPR^ER^ activation (Fig. 5C) and late-life proteostasis enhancement (Fig. 5D) induced by early-life *mys-1* RNAi, indicating that XBP-1 activity in early life is necessary to transduce NuA4 effects. To confirm this temporal XBP-1 requirement independently of NuA4, we utilized a transgenic (TG) strain with constitutive overexpression of XBP-1. This strain exhibited sustained UPR^ER^ activation (Fig. 5E) and improved late-life proteostasis (Fig. 5F). Strikingly, early-life *xbp-1* RNAi, confirmed by reduced *hsp-4p*::GFP fluorescence in L1/L2 (Fig. 5E), significantly diminished proteostasis enhancement observed in adult XBP-1 TG animals (Fig. 5F). Together, these findings demonstrate that XBP-1 activation specifically during development is vital for lifelong proteostasis maintenance.

### The XBP-1–SBP-1/MDT-15–FAT-7 axis is responsible for early-life NuA4-mediated proteostasis enhancement

**Figure 6:**
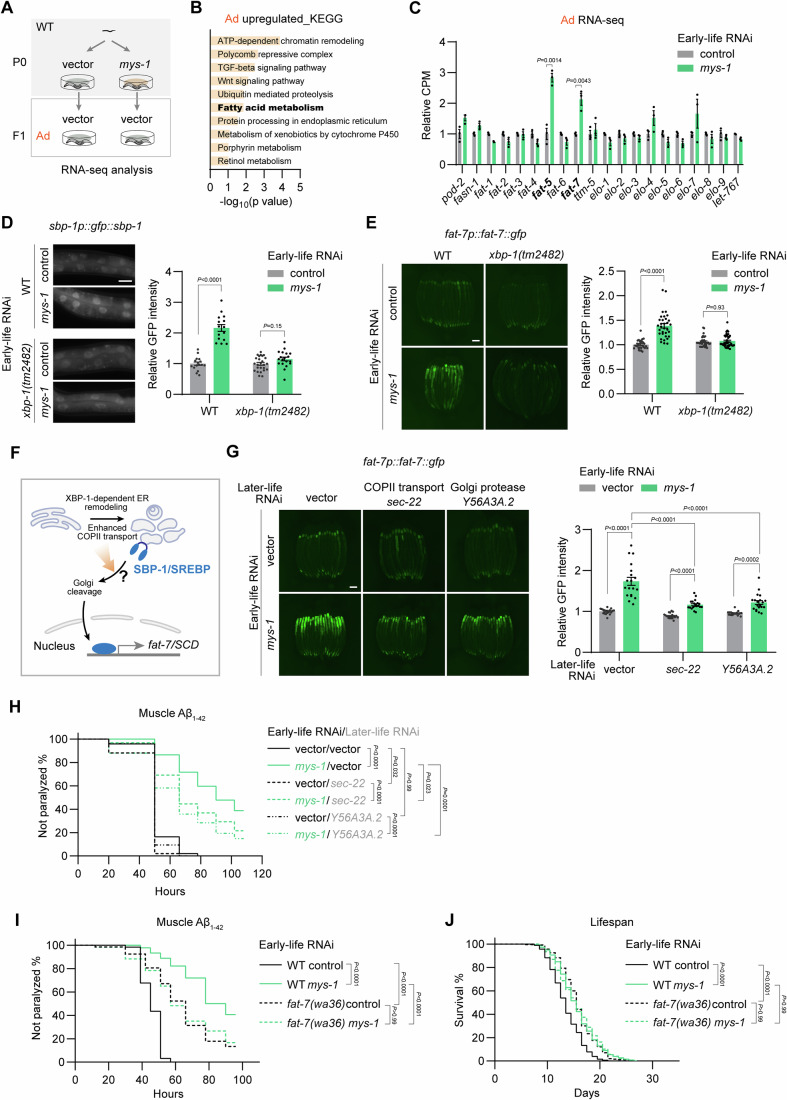
Early-life NuA4 mediates lifelong proteostasis and longevity through the XBP-1-ER-SBP-1/MDT-15-FAT-7 signaling axis. (**A**) Strategic design of the transcriptomic analysis in adult animals subjected to early-life *mys-1* RNAi. (**B**) KEGG enrichment analysis of upregulated genes in adult animals subjected to early-life *mys-1* RNAi. (**C**) Relative expression of fatty acid synthesis genes in the RNA-seq. Results are average ± SEM of three independent RNA-seq replicates. Statistical significance was assessed by a two-tailed unpaired *t*-test. (**D**) Representative images (left) and quantification (right) of nuclear SBP-1 levels in animals of the indicated genotypes following early-life *mys-1* RNAi treatment. Scale bar: 20 μm. Data were average ± SEM of three independent experiments. Animal number = 16 (WT control), 15 (WT *mys-1*), 25 (*xbp-1(tm2482)* control), 19 (*xbp-1(tm2482) mys-1*). Statistical significance was assessed by two-way ANOVA with multiple comparisons. (**E**) Representative images (left) and quantification (right) of FAT-7::GFP expression in animals of the indicated genotypes following early-life *mys-1* RNAi treatment. Scale bar: 100 μm. Data were average ± SEM of three independent experiments. Animal number = 30 (WT control), 33 (WT *mys-1*), 33 (*xbp-1(tm2482)* control), 41 (*xbp-1(tm2482) mys-1*). Statistical significance was assessed by two-way ANOVA with multiple comparisons. (**F**) Hypothetical diagram of XBP-1-ER remodeling in promoting SBP-1/SREBP precursor activation. (**G**) Representative images (left) and quantification (right) of FAT-7::GFP fluorescence levels in animals with early-life RNAi of *mys-1* and later-life RNAi with *sec-22* and *Y56A3A.2*. Scale bar:100 μm. Data were average ± SEM of three independent experiments. Animal number = 20 (vector/vector), 19 (*mys-1*/vector), 20 (vector/*sec-22*), 20 (*mys-1*/*sec-22*), 19 (vector/*Y56A3A.2*), 20 (*mys-1*/*Y56A3A.2*). Statistical significance was assessed by two-way ANOVA with multiple comparisons. (**H**) Paralysis of muscle Aβ_1–42_ animals with indicated RNAi treatment. Results are representative of three independent experiments. Statistical significance was assessed by the log-rank test. (**I**, **J**) Paralysis analysis of muscle Aβ_1–42_ animals (**I**) and lifespan assay (**J**) of WT and *fat-7(wa36)* animals following early-life *mys-1* RNAi treatment. Results are representative (**I**) or composite (**J**) of three independent experiments. Statistical significance was assessed by the log-rank test. Source data are available online for this figure.

To identify effectors downstream of the NuA4-H4K16ac-XBP-1-UPR^ER^ axis mediating late-life benefits, we performed RNA-seq analysis on adult animals subjected to early-life *mys-1* RNAi (Fig. 6A). Enrichment analysis of upregulated genes revealed significant enrichment of the lipid metabolism pathway, including a marked increase in expression of the delta-9 fatty acid desaturase genes *fat-5* and *fat-7* (Fig. 6B,C; Dataset EV3).

**Figure EV9:**
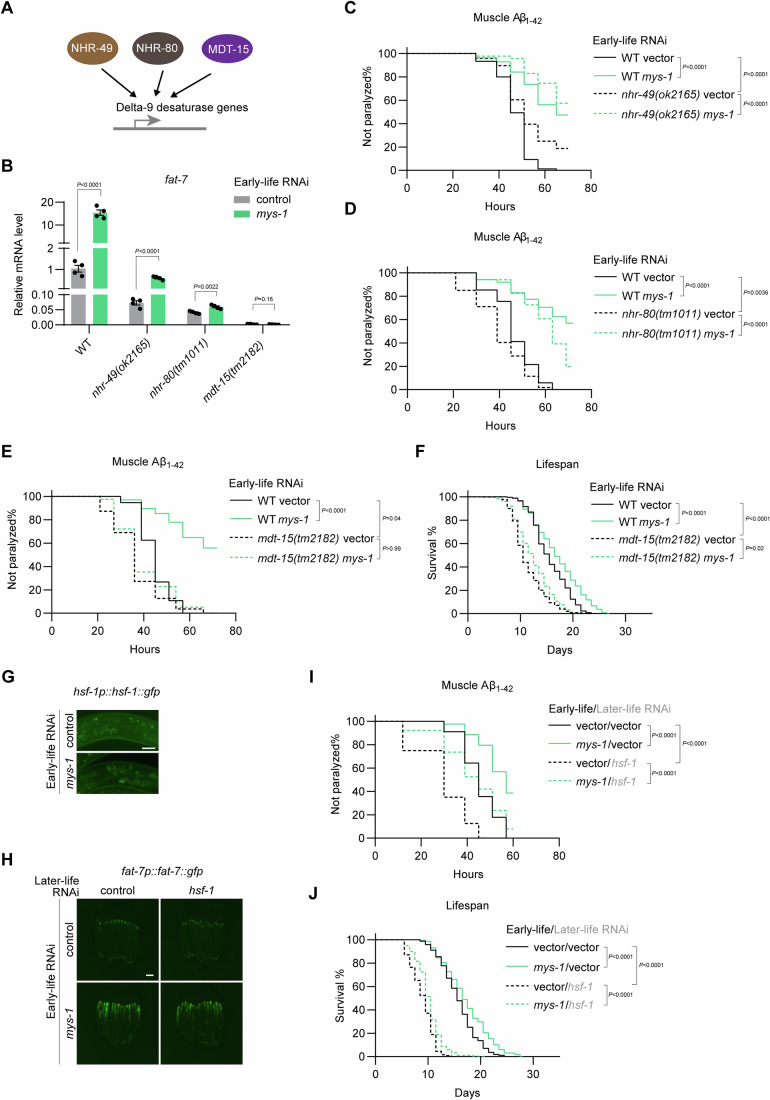
Early-life NuA4 governs lifelong proteostasis and longevity predominantly through the XBP-1-SBP-1/MDT-15-FAT-7 signaling axis. Related to Fig. 6. (**A**) Schematic diagram of transcriptional regulators of delta-9 desaturase genes. (**B**) Relative mRNA levels of *fat-7* in adult animals with the indicated genotype under early-life *mys-1* RNAi. Results are average ± SEM of three independent replicates. Statistical significance was assessed by a two-tailed unpaired *t*-test with FDR correction. (**C**–**E**) Paralysis analysis of muscle Aβ_1–42_ animals in WT and *nhr-49(ok2165)* (**C**)*, nhr-80(tm1011)* (**D**), and *mdt-15(tm2182)* (**E**) background, following early-life *mys-1* RNAi treatment. Results are representative of three independent experiments. Statistical significance was assessed by the log-rank test. (**F**) Lifespan analysis of WT and *mdt-15(tm2182)* animals under early-life *mys-1* RNAi. Results are a composite of three independent experiments. Statistical significance was assessed by the log-rank test. (**G**) Representative images of *hsf-1p::hsf-1::gfp* animals under early-life *mys-1* RNAi treatment. Scale bar: 20 μm. Results are representative of three independent experiments. Animal number >20 per group per experiment. (**H**) FAT-7 expression of the animals under early-life *mys-1* RNAi treatment and later-life inhibition of *hsf-1*. Scale bar: 100 μm. Results are representative of three independent experiments. Animal number >10 per group per experiment. (**I**,** J**) Paralysis analysis and Lifespan analysis of the animals under early-life *mys-1* RNAi treatment and later-life inhibition of *hsf-1*. Results are representative (**I**) or composite (**J**) of three independent experiments. Statistical significance was assessed by the log-rank test. Source data are available online for this figure.

Given that *fat-5* and *fat-7* are regulated by multiple transcription factors, we tested candidate upstream pathways (Fig. EV9A). Mutations in the nuclear receptors *nhr-49* and *nhr-80* did not prevent the upregulation of *fat-7* under early-life *mys-1* RNAi. In contrast, mutation in *mdt-15*, a known co-activator of the SBP-1 (Yang et al, 2006), completely abolished it (Fig. EV9B), pointing to a specific role for the SBP-1/MDT-15 axis. Indeed, early-life *mys-1* RNAi induced significant nuclear accumulation of SBP-1 and increased FAT-7 expression, both of which were entirely dependent on XBP-1 (Fig. 6D,E).

We next investigated how XBP-1 activation leads to SBP-1 nuclear accumulation. In mammalian systems, SREBP1 processing requires COPII-dependent vesicular trafficking from the ER to the Golgi, followed by proteolytic cleavage for activation (Shimano and Sato, 2017). We reasoned that the XBP-1-dependent ER expansion and whorl formation we observed (Fig. 4F) might reflect enhanced secretory capacity that facilitates SBP-1 activation (Fig. 6F). To test this, we genetically disrupted key components of the SREBP maturation pathway. Because knockdown of core COPII components (*sec-23, sec-24,* and *sar-1*) caused severe developmental arrest, we targeted *sec-22*, a SNARE protein that associates with COPII vesicles (Mancias and Goldberg, 2007) and is required for ER whorl formation under ER stress (Xu et al, 2021). Notably, *sec-22* RNAi significantly attenuated *fat-7* upregulation induced by early-life *mys-1* depletion (Fig. 6G). Similarly, knockdown of *Y56A3A.2*, the *C. elegans* homolog of the Golgi-resident SREBP protease, suppressed *fat-7* induction (Fig. 6G). Crucially, these perturbations not only reduced SBP-1 activity but also substantially diminished the proteostasis improvement conferred by *mys-1* RNAi (Fig. 6H), demonstrating that the XBP-1-dependent enhancement of SBP-1 maturation is functionally required for establishing long-term resilience.

To further confirm the functional requirement of the SBP-1/MDT-15 axis, we examined physiological outputs conferred by *mys-1* RNAi. While *nhr-49* and *nhr-80* mutations did not block the proteostasis improvement, the *mdt-15* mutation completely suppressed it (Fig. EV9C–E), and also attenuated the lifespan extension (Fig. EV9F). We then assessed *fat-7* mutants, as early-life *sbp-1* depletion itself is impractical due to the availability of only a hypomorphic allele and severe reproductive defects from RNAi. Strikingly, *fat-7* mutants completely abrogated both proteostasis enhancement and lifespan extension induced by early-life NuA4 suppression (Fig. 6I,J), establishing FAT-7 as the critical downstream effector.

Finally, given the recently reported crosstalk between the proteostasis regulator HSF-1 and lipid metabolism (Oleson et al, 2024), we assessed its potential role. Early-life RNAi of *mys-1* did not activate HSF-1 reporter (Fig. EV9G) or its target gene *hsp-16.2* (Fig. EV6C). Moreover, *hsf-1* RNAi failed to suppress FAT-7 induction, proteostasis improvement, or lifespan extension (Fig. EV9H–J), ruling out the involvement of HSF-1 in the pathway. Together, these findings delineate a specific signaling cascade essential for the lifelong benefits of developmental NuA4 perturbation: NuA4 → XBP-1 → ER remodeling → SBP-1/MDT-15 → FAT-7.

### Developmental oleic acid accumulation resulting from early-life NuA4 perturbation establishes lifelong proteostatic resilience

**Figure EV10:**
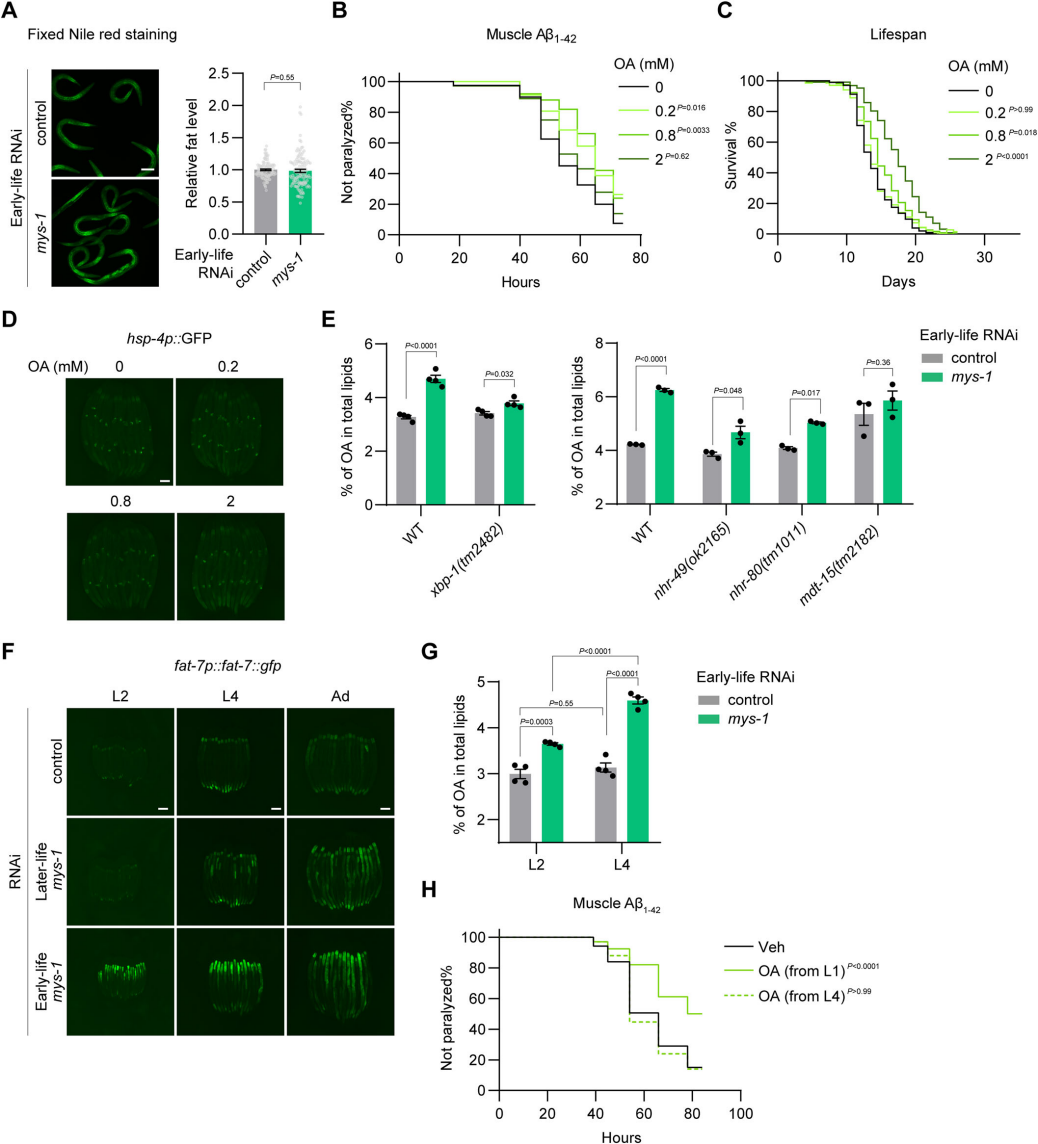
Developmentally accumulated oleic acid establishes adult proteostatic resilience. Related to Fig. 7. (**A**) Relative fat levels of animals subjected to early-life *mys-1* RNAi analyzed by Nile Red staining. Scale bar: 100 μm. Results are average ± SEM of three independent experiments. Animal number = 113 (control), 120 (*mys-1*). Statistical significance was assessed by a two-tailed unpaired *t*-test. (**B**,** C**) Paralysis analysis of muscle Aβ_1–42_ animals (**B**) and lifespan analysis of WT animals (**C**) with treatment of the indicated dose of OA. Results are representative (**B**) or composite (**C**) of three independent experiments. Statistical significance was assessed by the log-rank test. (**D**) Representative images of *hsp-4p::*GFP animals with treatment of the indicated dose of OA. Scale bar: 100 μm. Results are representative of three independent experiments. Animal number >10 per group per experiment. (**E**) Relative OA levels to the total fatty acids of animals with the indicated genetic background following early-life *mys-1* RNAi treatment. Results are average ± SEM of three or four independent replicates. Statistical significance was assessed by two-way ANOVA with multiple comparisons. (**F**) Representative images of FAT-7::GFP expression at the indicated stages following later-life and early-life *mys-1* RNAi. Scale bar: 100 μm. Results are representative of three independent experiments. Animal number >10 per group per experiment. (**G**) Relative OA levels to the total fatty acids of animals at the indicated stages following early-life *mys-1* RNAi treatment. Results are average ± SEM of four independent replicates. Statistical significance was assessed by two-way ANOVA with multiple comparisons. (**H**) Paralysis analysis of muscle Aβ_1–42_ animals treated with oleic acid (0.8 mM) from the L1 or L4 stage. Results are representative of three independent experiments. Statistical significance was assessed by the log-rank test. Source data are available online for this figure.

**Figure 7:**
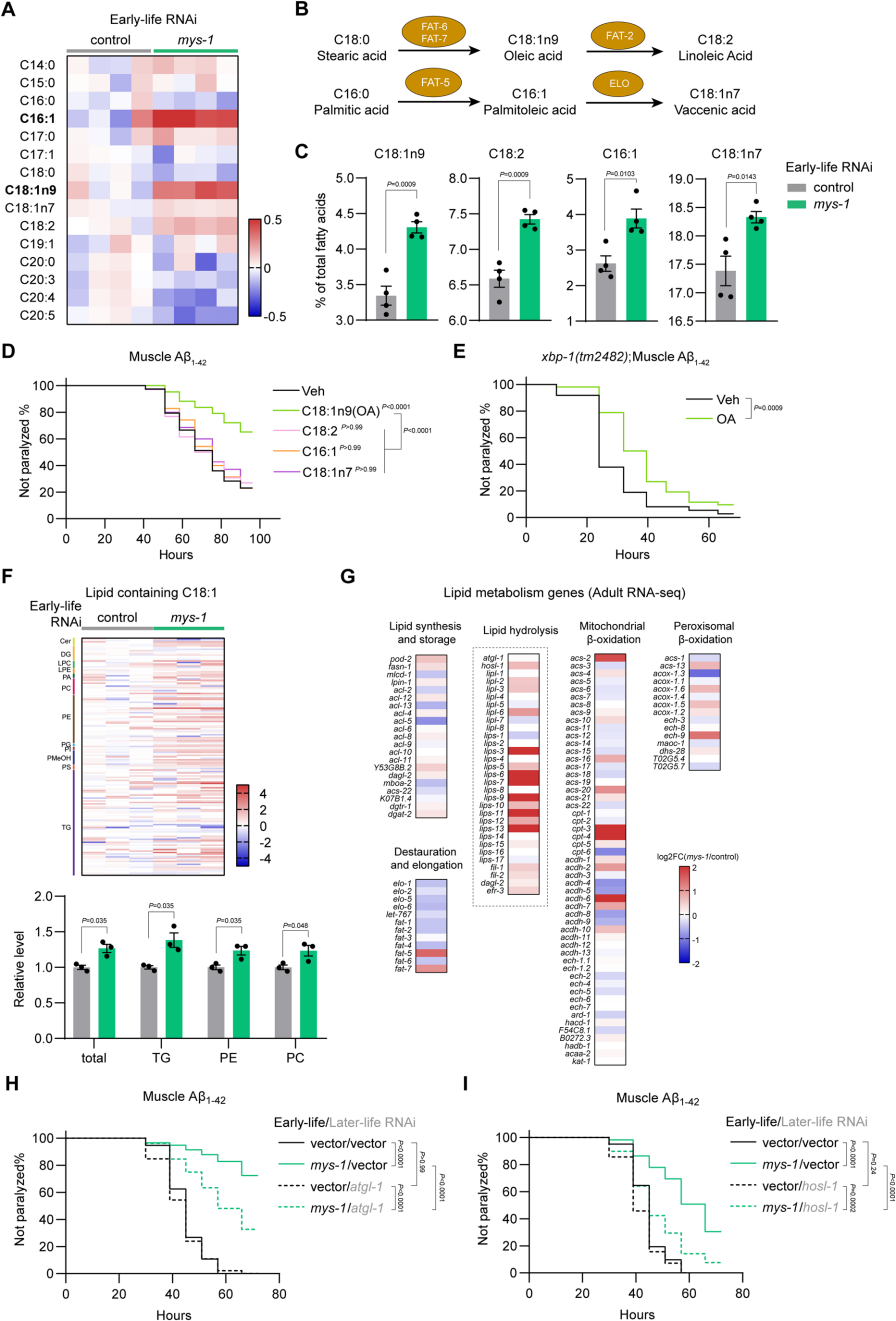
Early-life NuA4 drives developmental programming of lifelong proteostasis via oleic acid metabolism. (**A**) Heatmap of the relative changes of fatty acid composition in adult animals subjected to early-life *mys-1* RNAi. (**B**) Schematic diagram of fatty acid desaturation pathways. (**C**) Relative levels of the indicated fatty acids to the total fat level of adult animals subjected to early-life *mys-1* RNAi. Data were average ± SEM of four independent replicates. Statistical significance was assessed by a two-tailed unpaired *t*-test. (**D**) Paralysis analysis of muscle Aβ_1–42_ animals administered with the indicated fatty acids (0.8 mM). Results are representative of three independent experiments. Statistical significance was assessed by the log-rank test. (**E**) Paralysis analysis of muscle Aβ_1–42_ animals in the *xbp-1(tm2482)* background treated with oleic acid (0.8 mM). Results are representative of three independent experiments. Statistical significance was assessed by the log-rank test. (**F**) Heatmap and quantification of the relative changes of lipid species containing C18:1 in adult lipidomic analysis. Data were average ± SEM of three independent replicates. Statistical significance was assessed by a two-tailed unpaired *t*-test with FDR correction. (**G**) Heatmap showing the expression changes of lipid metabolism genes in adult RNA-seq (average of three independent replicates). (**H**, **I**) Paralysis analysis of muscle Aβ_1–42_ animals under early-life *mys-1* RNAi treatment, with later-life inhibition of the lipolysis genes *atgl-1* (**H**) and *hosl-1* (**I**). Results are representative of three independent experiments. Statistical significance was assessed by the log-rank test. Source data are available online for this figure.

While SBP-1/SREBP activation typically promotes lipogenesis and fat storage in *C. elegans* (Qin et al, 2022; Walker et al, 2011), early-life *mys-1* RNAi elicited no significant change in total fat levels in adults (Fig. EV10A) despite significantly upregulating specific genes (Fig. 6C). This suggests that the mechanism may depend on specific lipid species rather than total fat content. We therefore performed gas chromatography–mass spectrometry (GC-MS) analysis to profile fatty acid composition in adults subjected to early-life *mys-1* RNAi. This analysis revealed dramatic shifts in individual lipid species, most notably marked increases in palmitoleic acid (C16:1) and oleic acid (OA, C18:1n-9) (Fig. 7A; Dataset EV4). These increases correlated with and were mechanistically explained by elevated *fat-5* and *fat-7* expression (Fig. 6C), as both lipids are direct enzymatic products of FAT-5 and FAT-7 (Watts and Ristow, 2017) (Fig. 7B). Consistently, all downstream fatty acids produced by FAT-5/FAT-7 were significantly increased (Fig. 7C).

We focused on OA given its established links to longevity and proteostasis (Han et al, 2017; Imanikia et al, 2019; Xiao et al, 2023). Exogenous OA supplementation significantly protected against Aβ-induced paralysis in a dose-dependent manner, with 0.8 mM providing optimal benefit (Fig. EV10B,C). At this concentration, other elevated fatty acids (C16:1, C18:1n7, and C18:2) did not improve proteostasis (Fig. 7D), indicating specificity for OA. Furthermore, OA did not activate the UPR^ER^ (Fig. EV10D), while its supplementation conferred protection against Aβ-induced paralysis in *xbp-1(tm2482)* mutants (Fig. 7E), suggesting that it acts downstream of the UPR^ER^ signaling pathway.

Given that XBP-1 signaling during the L1 window is critical for programming, we hypothesized that the timing of the resultant OA increase might be equally important. Consistent with this, early-life *mys-1* RNAi induced sustained FAT-7 expression and led to a progressive accumulation of OA specifically throughout development (Fig. EV10F,G). Mirroring the temporal requirement for XBP-1, only OA supplementation initiated from L1, but not from the L4 stage onward, recapitulated the long-term proteostasis benefit (Fig. EV10H). This temporal constraint was further supported by the observation that later-life *mys-1* RNAi (initiated at L4) also induced FAT-7 expression but failed to improve adult proteostasis, paralleling the ineffectiveness of late OA supplementation (Fig. EV10F,H). Together, these data demonstrate that the developmental timing of both the upstream signal, XBP-1 activation, and the downstream effector (OA accumulation) is a crucial determinant of functional efficacy.

To elucidate how OA enhances proteostasis, we investigated its metabolic fate and functional utilization. Lipidomic analysis of adults revealed that OA was incorporated into multiple lipid species, including abundant triglycerides (TGs) and phospholipids, indicating engagement in both energy storage and membrane structure (Fig. 7F; Dataset EV5). Transcriptomic analysis of these adults showed significant upregulation of genes involved in lipolysis, the hydrolysis of stored TGs (Fig. 7G). This coordinated increase in OA-containing lipid species and lipolytic capacity, in the absence of net fat accumulation, suggested a model of enhanced lipid turnover. To test the functional importance of this lipolytic flux, we performed RNAi against two key lipases: adipose triglyceride lipase (*atgl-1*) and hormone-sensitive lipase (*hosl-1*). Knockdown of either lipase significantly attenuated the proteostasis improvement conferred by early-life *mys-1* RNAi (Fig. 7H,I), indicating that the protective effect requires not just OA accumulation, but its active mobilization from cellular stores

In summary, temporal NuA4 suppression during early life triggers a specific lipid remodeling program via XBP-1-SBP-1/MDT-15 axis, leading to developmentally timed OA accumulation. This OA pool, through mechanisms involving its regulated mobilization, establishes a metabolic state that confers lifelong proteostasis and longevity.

## Discussion

“A stitch in time saves nine”, this adage captures a timeless truth in medicine: early action prevents irreversible harm. Neurodegenerative diseases such as AD epitomize this concept, as late-stage therapeutic efforts often fail to reverse entrenched proteotoxic damage. Our findings shift the paradigm, showing that early-life plasticity can be actively harnessed to program lifelong resilience. In *C. elegans*, temporal suppression of a single gene, *mys-1*, encoding the NuA4 complex acetyltransferase mediating H4K16ac during early life is sufficient to enact durable proteostasis protection and lifespan extension. Mechanistically, we resolve that this early-life perturbation triggers sustained activation of the XBP-1-mediated UPR^ER^ stress response and developmental accumulation of the protective lipid OA. These changes establish a resilient cellular and metabolic state that persists into adulthood, effectively mitigating the toxicity of amyloid aggregation. Our findings resonate with the DOHaD concept, wherein early-life events shape late-life disease susceptibility. Crucially, however, we extend DOHaD beyond passive environmental exposures (e.g., malnutrition) (Barker and Osmond, 1986; Barker et al, 1993) to an active prevention strategy: targeted pathway manipulation during development proactively programs late-life resilience. Our work provides a mechanistic blueprint for future exploration of “stitch in time” developmental reprogramming approaches against neurodegeneration, intervening early to avert the profound costs of late-stage therapeutic futility.

Epigenetic regulation integrates early-life experiences with long-term gene expression programs, offering a mechanistic basis for how temporal developmental exposures can program lifelong health. Our study establishes a critical link between temporal reduction of NuA4 complex-mediated H4K16ac specifically during early life and enhanced organismal proteostasis and longevity. While we identified this intrinsic pathway, the specific environmental cues modulating H4K16ac levels via NuA4 in early development remain unknown. Potential candidates include metabolic factors influencing acetyl-CoA availability (Zhu et al; Ma et al), the key substrate for histone acetylation, as well as stressors like DNA damage (Hamsanathan et al, 2022), heat shock (Zhou et al, 2019), and pathogenic infections (Hong et al, 2021), all known to alter histone acetylation dynamics. Future studies should therefore investigate whether such environmental cues can modulate the NuA4-H4K16ac axis during early development, and crucially, whether this modulation confers similar long-term protective benefits as direct genetic perturbation.

A complete understanding also requires addressing the mechanistic nuance of the initiating event. The specificity of the H4K16ac reduction is clear, as levels of other marks like H3K9ac and H3K27ac remain unchanged. However, in *C. elegans* embryos, H4 acetylation at multiple lysine sites exhibits highly correlated genomic distributions (Liu et al, 2011). Thus, while H4K16ac serves as a robust and specific reporter, it likely functions as a representative and functionally critical component of a broader, coordinated H4 acetylation landscape established by the NuA4 complex. This presents a key question: how does the reduction of this mark trigger a precise response? Our CUT&Tag data showing *mys-1* depletion reduces H4K16ac at promoters of UPR^ER^-related genes leads us to propose a model of locus-specific depletion. This targeted chromatin alteration at critical regulatory sites, rather than a global reduction, may create the permissive state that gates the downstream cascade. Although our genetic interaction with the opposing SIN-3/HDA-3 histone deacetylase complex strongly suggests the phenotypic outcomes depend on the acetylation level set by MYS-1, we cannot formally exclude that NuA4 exerts additional scaffolding functions. Future studies employing locus-specific histone editing and catalytically dead MYS-1 will be essential to precisely delineate these contributions.

Intriguingly, H4K16ac has been linked to aging and AD pathogenesis (Nativio et al, 2018; Li et al, 2024). Our study now establishes a mechanistic link between this epigenetic mark and UPR^ER^ signaling, a key regulator of proteostasis. This connection is supported by an unbiased screen identifying NuA4 complex depletion alongside UPR^ER^ (Horowitz et al, 2023). Furthermore, we identify reduced expression of specific UPR^ER^ genes in embryos upon loss of NuA4-mediated H4K16ac and propose that this H4K16ac-dependent dysregulation induces a state of ER stress during embryogenesis. Notably, the UPR^ER^ possesses intrinsic feedback mechanisms to dynamically adjust protein-folding capacity (Walter and Ron, 2011; Kapulkin et al, 2005). Thus, NuA4 perturbation may trigger compensatory UPR^ER^ activation that not only restores homeostasis but also establishes enhanced resilience for future challenges. Supporting this model, inhibition of the opposing SIN-3/HDA-3 histone deacetylase complex restores ER homeostasis under stress (Taylor et al, 2024; Tillman et al, 2018). Consistent with these findings, we observe that *sin-3/hda-3* knockdown both resolves ER stress and abolishes proteostasis improvements induced by NuA4 perturbation, concomitant with H4K16ac level restoration. Collectively, our results reveal a previously unrecognized role for H4K16ac in regulating ER proteostasis and align with the hormesis hypothesis (Shindyapina et al, 2022; Sun et al, 2009; Jiang et al, 2024), wherein early-life subtoxic stress confers late-life benefits. Crucially, the timing and intensity of stress are critical determinants of outcome (Dillin et al, 2002b). While our work defines the benefits of early-life perturbation, the detrimental effects of late-life NuA4 suppression that may stem from excessive ER stress exceeding compensatory capacity or disruption of other NuA4’s essential functions remain to be explored.

Our findings underscore the early-life “window of opportunity” to reinforce proteostasis capacity and promote lifespan before irreversible collapse occurs. Aging is increasingly recognized as stage-specific: insulin/IGF-1 signaling modulates longevity by tuning adult stress responses and metabolism (Dillin et al, 2002a), while HSF-1 plays a critical role in proteostasis regulation during aging through modulation of developmental chaperone networks (Volovik et al, 2012). We here identify XBP-1 as a key developmental regulator of proteostasis, functioning predominantly in early larval stages. This suggests a mechanism of developmental plasticity influencing late-life disease susceptibility: XBP-1-mediated metabolic priming. Through persistent activation of the SBP-1–FAT-7–OA synthesis axis during development, this priming establishes a resilient cellular state that mitigates late-life proteotoxicity. An important future direction will be to determine how the amplitude, duration, and spatial pattern of this developmental XBP-1 signal are calibrated to achieve long-term benefit, potentially through the use of precisely controlled induction systems. Such research could help define the parameters for the therapeutic interventions aimed at recapitulating this adaptive programming.

Strikingly, elevated OA levels are a shared feature across diverse longevity models, including those with ectopic *xbp-1s* overexpression (Imanikia et al, 2019), COMPASS complex depletion (Han et al, 2017), and germline stem cell ablation (Castillo-Quan et al, 2023; Goudeau et al, 2011), supporting the physiological relevance of our findings. In the context of early-life NuA4 perturbation, we find that OA’s benefit requires its active mobilization via lipolysis, suggesting that developmentally accumulated OA pools are not merely static reservoirs but are functionally accessed through lipolytic flux. It is plausible that distinct upstream signals that elevate OA may engage different downstream effector mechanisms, such as lipid droplet (Papsdorf et al, 2023), membrane remodeling (Peng et al, 2011; Zeng et al, 2020) or ERAD enhancement (Castillo-Quan et al, 2023), highlighting context-dependent roles for this key lipid.

Future work should investigate whether OA-associated longevity arises universally from developmental programming. Notably, the OA-rich Mediterranean diet, which contains a complex mixture of beneficial MUFAs and PUFAs, has been epidemiologically linked to reduced risk of cardiovascular and neurodegenerative diseases (Tapsell, 2014; Lee et al, 2016). While our study identifies OA as a specific critical mediator within a defined genetic pathway, the broader health benefits of such diets likely arise from the synergistic effects of multiple lipid species. Future studies are warranted to determine if early OA supplementation, or targeted modulation of its associated pathways, can prophylactically establish resilient cellular states to combat human diseases resulting from proteostasis decline.

## Methods

**Table:** Reagents and tools table

Reagent/resource	Reference or source	Identifier or catalog number
**Experimental models**		
*C. elegans:* N2	CGC	Cat# N2
*C. elegans: dvIs100[unc-54p::A-beta-1–42::unc-54 3’-UTR + mtl-2p::GFP]*.	Dr. Suhong Xu, Zhejiang University	Cat# GMC101
*C. elegans: smg-1(cc546) I; dvIs27[myo-3p::A-Beta 1–42::let-851 3’UTR) + rol-6(su1006)] X*.	Dr. Suhong Xu, Zhejiang University	Cat# CL4176
*C. elegans: smg-1(cc546) dvIs50[pCL45 (snb-1::Abeta 1–42::3’ UTR(long) + mtl-2::GFP] I*.	CGC	Cat# CL2355
*C. elegans: pkIs2386 [unc-54p:: a-syn::YFP + unc-119(+)]*.	CGC	Cat# NL5901
*C. elegans: baIn11[Pdat-1::a-syn; Pdat-1::GFP]*	Dr. Xiajing Tong, Shanghai Tech University	Cat# UA44
C. elegans: wrdSi50[mex-5p::TIR1::F2A::BFP::AID*::NLS::tbb-2 3’UTR] I.	Dr. Zhuo Du, CAS	Cat# JDW221
C. elegans: wrdSi50[eft-3p::TIR1::F2A::BFP::AID*::NLS::tbb-2 3’UTR] I.	Dr. Zhuo Du, CAS	Cat# JDW225
*C. elegans: zcls4[hsp-4p::GFP] V*	CGC	Cat# SJ4005
*C. elegans: xbp-1(tm2482) III*	Dr. Bin Qi, Yunnan University	Cat# TM2482
*C. elegans: ire-1(zc14) II*	Dr. Ben Zhou, CAS	Cat# SJ30
*C. elegans: atf-6(ok551) X*	CGC	Cat# RB772
*C. elegans: pek-1(ok275) X*	CGC	Cat# RB545
*C. elegans: ftISf[epEx307[unc-119(+);sbp-1p::gfp::sbp-1]*	Dr. Bin Liang, Yunnan University	Cat# KQ377
*C. elegans: lin-15(n765)X; waEx15[fat-7WG::GFP;lin-15(+)]*	Dr. Bin Liang, Yunnan University	Cat# BX113
*C. elegans: fat-7(wa36) V*	CGC	Cat# BX153
*C. elegans: nhr-49(ok2165) I*	CGC	Cat# RB1716
*C. elegans: nhr-80(tm1011) III*	CGC	Cat# BX165
*C. elegans: mdt-15(tm2182) III*.	Dr. Di Chen, Zhejiang University	Cat# XA7702
*C. elegans: drSi13 [hsf-1p::hsf-1::GFP::unc-54 3’UTR + Cbr-unc-119(* + *)] II; unc-119(ed3) III*	Dr. Di Chen, Zhejiang University	Cat# OG497
*C. elegans: zcls13[hsp6::GFP] V*	CGC	Cat# SJ4100
*C. elegans: gpIs1 [hsp-16.2p::GFP]*.	CGC	Cat# TJ375
*C. elegans: mys-1(aly33[3xflag::mys-1]) V*	This study	Cat# WLU173
C. elegans: mys-1(aly34[aid::gfp::3xflag::mys-1]) V; wrdSi50[mex-5p::TIR1::F2A::BFP::AID*::NLS::tbb-2 3’UTR] I.	This study	Cat# WLU215
C. elegans: dvIs100[unc-54p::A-beta-1–42::unc-54 3’-UTR + mtl-2p::GFP]; mys-1(aly34[aid::gfp::3xflag::mys-1]) V; [mex-5p::TIR1::F2A::BFP::AID*::NLS::tbb-2 3’UTR] I.	This study	Cat# WLU327
C. elegans: dvIs100[unc-54p::A-beta-1–42::unc-54 3’-UTR + mtl-2p::GFP]; mys-1(aly34[aid::gfp::3xflag::mys-1]) V; [eft-3p::TIR1::F2A::BFP::AID*::NLS::tbb-2 3’UTR] I.	This study	Cat# WLU479
C. elegans: zcls4[hsp-4p::GFP]; mys-1(aly34[aid::gfp::3xflag::mys-1]) V; [mex-5p::TIR1::F2A::BFP::AID*::NLS::tbb-2 3’UTR] I.	This study	Cat# WLU442
C. elegans: zcls4[hsp-4p::GFP]; mys-1(aly34[aid::gfp::3xflag::mys-1]) V; [eft-3p::TIR1::F2A::BFP::AID*::NLS::tbb-2 3’UTR] I.	This study	Cat# WLU585
*C. elegans: alyEx111[xbp-1p::xbp-1::gfp::aid+myo-2::mCherry]*	This study	Cat# WLU570
*C. elegans: alyEx111[xbp-1p::xbp-1::gfp::aid+myo-2::mCherry];dvIs100[unc-54p::A-beta-1–42::unc-54 3’-UTR + mtl-2p::GFP]*	This study	Cat# WLU580
*C. elegans: alyEx111[xbp-1p::xbp-1::gfp::aid+myo-2::mCherry];zcls4[hsp-4p::GFP]*	This study	Cat# WLU601
*C. elegans: dvIs100[unc-54p::A-beta-1–42::unc-54 3’-UTR + mtl-2p::GFP]; xbp-1(tm2482) III*	This study	Cat# WLU328
*C. elegans: dvIs100[unc-54p::A-beta-1–42::unc-54 3’-UTR + mtl-2p::GFP]; ire-1(zc14) II*	This study	Cat# WLU405
*C. elegans: dvIs100[unc-54p::A-beta-1–42::unc-54 3’-UTR + mtl-2p::GFP]; atf-6(ok551) X*	This study	Cat# WLU434
*C. elegans: dvIs100[unc-54p::A-beta-1–42::unc-54 3’-UTR + mtl-2p::GFP]; pek-1(ok275) X*	This study	Cat# WLU432
*C. elegans: dvIs100[unc-54p::A-beta-1–42::unc-54 3’-UTR + mtl-2p::GFP]; fat-7(wa36) V*	This study	Cat# WLU336
*C. elegans: xbp-1(tm2482); waEx15[fat-7WG::GFP;lin-15(+)]*	This study	Cat# WLU289
*C. elegans: xbp-1(tm2482); ftISf[epEx307[unc-119(+); sbp-1p::gfp::sbp-1]*	This study	Cat# WLU291
*C. elegans: dvIs100[unc-54p::A-beta-1–42::unc-54 3’-UTR + mtl-2p::GFP];nhr-49(ok2165) I*	This study	Cat# WLU712
*C. elegans: dvIs100[unc-54p::A-beta-1–42::unc-54 3’-UTR + mtl-2p::GFP];nhr-80(tm1011) III*	This study	Cat# WLU713
*C. elegans: dvIs100[unc-54p::A-beta-1–42::unc-54 3’-UTR + mtl-2p::GFP];mdt-15(tm2182) III*.	This study	Cat# WLU714
*C. elegans: mys-1(aly34[aid::gfp::3xflag::mys-1]);dvls100[unc-54p::A-beta-1–42::unc-54 3’-UTR + mtl-2p::GFP]; reSi7 [rgef-1p::TIR1::F2A::mTagBFP2::NLS::AID::tbb-2 3’UTR]*	This study	Cat# WLU717
*C. elegans: mys-1(aly34[aid::gfp::3xflag::mys-1]);dvls100[unc-54p::A-beta-1–42::unc-54 3’-UTR + mtl-2p::GFP];reSi5 I [ges-1p::TIR1::F2A::mTagBFP2::NLS::AID::tbb-2 3’UTR]*	This study	Cat# WLU587
*E.coli*: OP50	CGC	Cat# OP50; RRID:WB-STRAIN:OP50
*E.coli*: HT115	CGC	Cat# HT115(DE3); RRID:WB-STRAIN:HT115(DE3)
**Recombinant DNA**		
pPD49.26-xbp-1p-xbp-1(gdna)-gfp-aid	This study	N/A
**Antibodies**		
Rabbit recombinant monoclonal histone H4 acetyl K16 antibody	Abcam	Cat# ab109463; RRID:AB_10858987
Rabbit polyclonal histone H4 antibody	CST	Cat# 2592; RRID:AB_2118614
Mouse anti-DYKDDDDK tag (FLAG) antibody	HUABIO	Cat# M1403-2; RRID:AB_3073075
Rat anti-tubulin antibody [YL1/2]	Abcam	Cat# ab6160; RRID:AB_305328
Anti-β-amyloid, 1–16 antibody	BioLegend	Cat#803014; RRID:AB_2728527
GFP (D5.1) Rabbit monoclonal antibody	CST	Cat#2956; RRID:AB_3713179
Anti-histone H3 (acetyl K27) antibody	Abcam	Cat#ab177178; RRID:AB_2828007
Acetyl-histone H3 (Lys9) (C5B11) rabbit monoclonal antibody	CST	Cat#9649; RRID:AB_823528
Anti-histone H3 antibody	Abcam	Cat#ab1791; RRID:AB_302613
**Oligonucleotides and other sequence-based reagents**		
*xbp-1* total pPCR F:CCGATCCACCTCCATCAAC	This study	N/A
*xbp-1* total pPCR R:ACCGTCTGCTCCTTCCTCAATG	This study	N/A
*xbp-1* spliced pPCR F:TGCCTTTGAATCAGCAGTGG	This study	N/A
*xbp-1* spliced pPCR R:ACCGTCTGCTCCTTCCTCAATG	This study	N/A
*Aβ* qPCR F: GACGCGGATGCAGAATTCC	This study	N/A
*Aβ* qPCR R:CAACACCGCCCACCATGAGT	This study	N/A
*α-synuclein* qPCR F:TGGGCAAGAATGAAGAAGGAG	This study	N/A
*α-synuclein* qPCR R:GTTCGTAGTCTTGATACCCTTCC	This study	N/A
**Chemicals, enzymes and other reagents**		
K-NAA	Sangon Biotech	Cat# A600730
IAA-AM	Synthesized by Shanghai Nafu Biotech	Negish et al^88^
Oleic acid sodium	Sigma	Cat# O7501
Linoleic acid	Sigma	Cat# L8134
Palmitoleic acid	Sigma	Cat# P9417
Vaccenic acid	Sigma	Cat# V0384
Chemiluminescent detection reagents	Thermo Fisher	Cat# 34580
Nitrocellulose membrane	Millipore	Cat# HATF00010
Nile Red	BBI Life Science	Cat# 7385-67-3
Benzaldehyde	Sigma	Cat# 418099
Alt-R™ S.p. Cas9 Nuclease V3	IDT	Cat# 1081058
TRIzol Reagent	Cwbio	Cat# CW0580S
Protein A-Tn5	MacroLab	Cat# p2409
cOmplete EDTA-free Protease Inhibitor Cocktail	Roche	Cat# 05056489001
SPRIselect Beads	Beckman coulter	Cat# B23319
**Software**		
ImageJ	https://imagej.nih.gov/ij/	N/A
GraphPad Prism 8.0	https://www.graphpad.com/	N/A
Database for annotation, visualization and integrated discovery (DAVID)	https://davidbioinformatics.nih.gov/	N/A
Trim Galore!	https://www.bioinformatics.babraham.ac.uk/projects/trim_galore/	N/A
Cutadapt	https://cutadapt.readthedocs.io/en/stable/	N/A
Bowtie2	https://sourceforge.net/projects/bowtie-bio/files/bowtie2/2.4.1/	N/A
Sambamba	https://github.com/biod/sambamba	N/A
deepTools	https://deeptools.readthedocs.io/en/develop/index.html	N/A
IGV	https://igv.org/doc/desktop/	N/A
MACS3	https://macs3-project.github.io/MACS/	N/A
bedtools	https://bedtools.readthedocs.io/en/latest/index.html	N/A
HOMER	http://homer.ucsd.edu/homer/	N/A
featureCounts	https://subread.sourceforge.net/	N/A
edgeR	https://bioconductor.org/packages/release/bioc/html/edgeR.html	N/A
R	https://www.r-project.org/	N/A

### *C. elegans* strains and culture

The wild-type *C. elegans* strain was Bristol N2. Unless otherwise specified, all strains were maintained on nematode growth media (NGM) seeded with *Escherichia coli* OP50 as a food source under standard culture conditions at 20 °C (Brenner, 1974). All experiments were conducted using hermaphrodites.

### Bacterial strains

The *E. coli* strains OP50 and HT115(DE3) were obtained from CGC. RNAi constructs were obtained from either the Ahringer-RNAi library or the ORF-RNAi library, or were constructed in this study.

### Annotation of early-life genes in *C. elegans*

A list of genes with specific early-life expression patterns was compiled based on analysis of a published RNA-seq dataset encompassing the entire *C. elegans* lifecycle (embryonic stages from four-cell collected at 30-min intervals, L1-L4 larvae, young adult, and adult) (Boeck et al, 2016). Gene expression in this dataset was quantified using density-corrected counts per million (DCPM), which adjusts for sequencing depth and gene length. Analysis of the DCPM distribution across all genes revealed that the 80th percentile value was 1.06, indicating that ~20% of genes had a DCPM value above 1 and were therefore considered highly expressed genes. Based on this distribution, active early-life genes were defined as those meeting the following criteria:

- High expression in early stages: Mean DCPM (embryonic stages through L1) >1.
- Low expression in later stages: Mean DCPM (L2 through young adult) <1.
- Early-life specific expression: Mean DCPM (embryonic through L1)/mean DCPM (L2 through young adult) >2.

Application of these criteria yielded a list of 1059 candidate early-life genes.

### RNAi feeding in *C. elegans*

RNAi feeding experiments were performed as previously described (Wu et al, 2016). Briefly, HT115(DE3) bacteria expressing the targeted double-stranded RNA (dsRNA) were sequence-verified and seeded onto NGM plates supplemented with 1 mg/mL IPTG and 25 μg/mL carbenicillin. HT115(DE3) containing the empty vector L4440 was used as the control. Seeded plates were dried in a hood and incubated at room temperature for 24 h before use.

For early-life RNAi, parental generation (P0) hermaphrodites were fed with HT115 bacteria expressing the targeted dsRNA from the L1 larval stage until Day 1 of adulthood. P0 adults were then bleached to isolate embryos (F1). These F1 embryos were transferred onto control RNAi plates (HT115 containing empty vector L4440) and grown to the indicated stages for assessment.

For later-life RNAi experiments, P0 hermaphrodites were fed empty vector RNAi bacteria prior to bleach treatment to isolate F1 embryos. The F1 embryos were then transferred to plates seeded with the specified RNAi bacteria and cultured until the desired stages for assessment.

### Construction of the KI strains

Strains harboring MYS-1 knock-in alleles were generated using CRISPR/Cas9-mediated genome editing as previously described (Dokshin et al, 2018; Ghanta and Mello, 2020). Briefly, a single guide RNA (sgRNA) was designed using the online CRISPR Design Tool (http://crispr.mit.edu) and synthesized by Genscript. The double-stranded DNA (dsDNA) donor template for homology-directed repair (HDR) was generated by PCR using primers containing 35-nucleotide homology arms flanking the target site, followed by purification and melting. The melted dsDNA donor and co-injection marker plasmid *rol-6(su1006)* were added to sgRNA and Cas9 Nuclease (IDT) ribonucleoprotein complexes before injection. Successful knock-in events were selected by PCR genotyping from the F1 that were produced after the roller cohort of progeny.

### Auxin-induced degradation assay

For maternal protein degradation, a synthetic, water-soluble auxin, 1-naphthaleneacetic acid potassium salt (K-NAA) was used. A 500 mM K-NAA stock solution in ddH_2_O was added to the melted NGM agar at 55 °C before pouring plates to a final concentration of 4 mM (Ashley et al, 2021). The plates were kept at 4 °C for no longer than 2 weeks, with HT115 (L4440) seeded 1–2 days before use. Auxin treatment was performed by transferring L4 worms to plates with or without 4 mM K-NAA. The next day, the P0 adults were harvested to isolate embryos (F1) by bleach. F1 embryos were transferred to control plates (seeded with HT115(L4440) but without auxin) and grown to the indicated stage for phenotypic assessment.

For embryonic protein degradation, an eggshell-permeable auxin indole-3-acetic acid aminoethyl ester (IAA-AM) (Negishi et al, 2019) (synthesized by Shanghai Nafu Biotechnology) was used. A 500 mM stock solution in DMSO was diluted in M9 buffer to a final concentration of 1 mM. Embryos isolated by bleach treatment were soaked in M9 buffer containing 1 mM IAA-AM or control buffer (with equivalent DMSO) for the specified duration. Following treatment, the hatched L1 animals were transferred to NGM plates seeded with HT115 (L4440) and grown to the indicated stage for phenotype assessment.

### Imaging and fluorescence quantification

Expression of *hsp-4* and *fat-7* was evaluated using *hsp-4p::gfp* and *fat-7p::fat-7::gfp* transgenic strains, respectively. Worms were paralyzed on a 2% agar plate containing 50 mM sodium azide. The images were captured under 5× magnification by Leica DM500 Microscope with fixed exposure parameter (*n* > 15 animals/group). For imaging of *hsp-4p::gfp* at the L1 stage, the synchronized embryos were incubated in M9 buffer at 20 °C for 20 h. The hatched L1 animals were anesthetized with 1 mg/mL levamisole and mounted on 2% agarose pads. The images were captured under 20× magnification by a Leica DM500 Microscope with fixed exposure parameters (*n* > 20 animals/group).

To obtain images of intestinal GFP::SBP-1 expression, *sbp-1p::gfp::sbp-1* worms in the late L4 stage were used. The worms were anesthetized in M9 buffer containing 1 mg/mL levamisole and mounted on 2% agarose pads before imaging. The images were captured under 40× magnification by a Leica DM500 Microscope with fixed exposure parameters (*n* > 15 animals/group). Nuclear GFP fluorescence intensity (arbitrary units, a.u.) was quantified using ImageJ after subtracting background signal from adjacent areas. Data were presented as relative fluorescence units normalized to the average intensity of control animals.

Protein aggregation and neurodegeneration were evaluated in day 5 adult NL5901 (expressing muscle α-SYN::YFP) and UA44 (expressing α-SYN in dopaminergic neurons with GFP marker) strains. Worms were anesthetized and mounted as described above. YFP aggregates in body wall muscle and GFP-positive dopaminergic neurons in the head were counted and imaged under 20× magnification using fixed exposure parameters (*n* > 30 animals/group).

### TEM sample preparation and imaging

Late L4-stage wild-type (N2) and *xbp-1(tm2482)* hermaphrodites were processed for transmission electron microscopy (TEM) using an established protocol (Zhang et al, 2023). Briefly, worms were transferred into 200 μL of M9 buffer containing 20% BSA and then frozen using a high-pressure freezing machine (Leica EM ICE). Frozen samples underwent freeze-substitution in a Leica EM AFS2 freeze-substitution unit with a medium containing 1% osmium tetroxide, 0.1% uranyl acetate, and 10% methanol in acetone (Manning and Richmond, 2015). Following freeze-substitution, samples were warmed to 20 °C and washed 3× with pure acetone. Gradual resin infiltration was performed using Epon resin diluted in acetone at increasing concentrations: 25% Epon resin for 30 min, 33% for 210 min, 50% for 12 h, 75% for 240 min, and 100% resin for four times during a 24-h period. Finally, samples were embedded in a mold and cured at 60 °C for 48 h.

For TEM imaging, the embedded samples were sliced into 70 nm sections using a Leica EM UC7 ultramicrotome, and then deposited onto copper grids. The sections were subsequently counterstained with 2% uranyl acetate followed by Sato’s triple lead stain. The imaging was performed using a Thermo Fisher Scientific Talos L120C G2 TEM operating at 80 kilovolts with a 2k × 2k Ceta CCD camera.

### Aβ-induced paralysis assay

L4-stage GMC101 (muscle Aβ_1–42_) animals (McColl et al, 2012) with designated RNAi were transferred to assay plates (*n* > 50 animals/group), upshifted from 20 to 25 °C, and monitored for paralysis. Animals were determined as paralyzed if they failed to complete full body movement in response to the gentle touch of the worm pick. Animals were moved to fresh plates as necessary to avoid contamination with their progeny. The number of paralyzed and non-paralyzed animals was counted every 6 h. GMC101 crossed with *xbp-1(tm2482)* and *ire-1(zc14)* mutants were found to be sick and sterile at 20 °C. Therefore, those genotypes, together with the GMC101 WT control animals, were maintained at 15 °C before experiments (including early-life RNAi treatment), and the experimental population (F1) was kept at 20 °C and upshifted to 25 °C at the L4 stage for paralysis assessment.

CL4176 (muscle Aβ_3–42_) animals were maintained at 15 °C as previously described (Link, 2006). Synchronized populations reaching the L3 stage were shifted to 25 °C and monitored for paralysis using the scoring criteria above. Data from all paralysis assays presented in the Figures are representative of at least three trials. Details and statistics can be found in Dataset EV6.

We conducted the early-life RNAi screen, using GMC101 animals, with two blind, independent replicates, after which we focused on the function of the NuA4 complex. The screening outcomes were assessed at 36 and 60 h post-temperature upshift. At 36 h, the control animals are mostly non-paralyzed (score = 0, ~10% paralyzed), and the candidate with aggravated symptoms were scored based on the severity compared to the control RNAi: score = −1, ~50% paralyzed; score = −2, ~90% paralyzed. At 60 h, the control animals are mostly paralyzed (score = 0, ~10% non-paralyzed), and the candidates with alleviated symptoms were scored based on the severity compared to the control RNAi: score = 1, ~50% non-paralyzed; score = 2, ~90% non-paralyzed. The representative images of each score at the indicated time were shown in Dataset EV1.

### Chemotaxis assay

CL2355 animals expressing neuronal Aβ_3–42_ (Luo et al, 2009) were maintained at 15 °C. Synchronized L3 larvae were upshifted to 25 °C until day 1 adulthood. Adult worms were washed 3× in M9 buffer and once in ddH_2_O, with gravity sedimentation between washes. After 1-h starvation on unseeded NGM plates, animals (>50 per assay) were transferred to assay plates. The 10 cm chemotaxis assay plates were prepared with a 1 µL spot of ethanol (vehicle) and a 1 µL spot of 0.1% benzaldehyde in ethanol (attractant odorant) on opposite sides. 1 µL of sodium azide (50 mM) was added to the vehicle and odorant spots. Worms were positioned at the center of the plate and kept in the dark for 1 h at room temperature before counting. Chemotaxis index (CI) is calculated as CI = (number of worms at the attractant location - number of worms at the vehicle location)/total number of worms on the plate.

### Lifespan assay

Lifespan analyses were conducted on NGM plates seeded with HT115 bacteria expressing designated RNAi constructs. Animals were synchronized by bleach and grown at 20 °C. Adult animals were manually moved to fresh plates every day or every other day during reproduction as necessary and scored every day for mortality until the last worm died. Animals with bagging and vulval explosions were censored. All lifespan experiments were performed by two to three independent researchers. Data presented in Figures are composites of at least two independent biological replicates, with the total number >200. Details and statistics can be found in Dataset EV6.

### RNA-seq and analysis

RNA samples were collected as follows: parental generation (P0) hermaphrodites were fed with HT115(DE3) bacteria, with empty vector L4440 or *mys-1* RNAi from the L1 larval stage until Day 1 of adulthood. P0 adults were then bleached to isolate embryos (F1) in M9 buffer. F1 samples were collected at three time points: embryos immediately after bleach treatment (E0), embryos 6 h post-bleach treatment (E6), and L1 larvae 24 h after bleach treatment (L1). The F1 embryos were also transferred onto L4440 plates and cultured for 70 h prior to the collection of adult animals. All samples were frozen in Trizol prior to RNA extraction.

The RNA extraction and sequencing were performed by Novogene Corporation Inc. Total RNA of worms of the indicated stages was extracted with Trizol. The quality and quantity of the RNA were assessed using the NanoPhotometer® spectrophotometer (IMPLEN) and Qubit® RNA Assay Kit in Qubit®2.0 Flurometer (Life Technologies). The integrity of RNA was evaluated with the RNA Nano 6000 Assay Kit of the Bioanalyzer 2100 system (Agilent Technologies). Sequencing libraries were prepared using the NEBNext® UltraTM RNA Library Prep Kit for Illumina® (NEB, USA), following the manufacturer’s instructions. Index codes were added to assign sequences to each sample. The index-coded samples were clustered using the TruSeq PE Cluster Kit v3-cBot-HS (Illumina) on a cBot Cluster Generation System, according to the manufacturer’s guidelines. Libraries were then sequenced on an Illumina platform, generating 125/150 bp paired-end reads.

The sequencing reads were de-multiplexed using bcl2fastq and quantified using kallisto 0.46.0, with default parameters to the WormBase reference genome (WS274). For differential gene expression analysis, genes with low expression levels were filtered out, retaining only those genes with read values greater than ten in all replicates. Differential gene expression was analyzed using DESeq2 1.26.0, and statistical significance was determined based on false discovery rate (FDR)-adjusted *p* values less than 0.05. Functional enrichment analysis of gene clusters was performed using GO term and KEGG pathway analyses with the Database for Annotation, Visualization and Integrated Discovery (DAVID) (Sherman et al, 2022).

### CUT&Tag

CUT&Tag samples (E0, E6, and L1) were collected as described in the RNA-seq method section. Approximately 100,000 embryos or L1 larvae were pelleted in ddH_2_O, snap-frozen in liquid nitrogen Frozen worm pellets were crushed to isolate single nuclei using gentleMACS™ Dissociator (400×*g*, 1 min, 600×*g*, 1 min, 4 °C) and gentleMACS™ M Tubes in MACS Buffer (10 mM Tris-HCl pH 7.4, 3 mM MgAc_2_, 5 mM CaCl_2_, 2 mM EDTA, 0.6 mM DTT, and 1×Protease Inhibitor Cocktail) and were filtered through 30 µm Celltrics^TM^ filters. After centrifugation (500×*g*, 10 min, 4 °C), pellets were permeabilized in OMNI Buffer (10 mM Tris-HCl, pH 7.4, 10 mM NaCl, 3 mM MgCl_2_, 0.1% Tween-20, 0.1% NP-40, and 0.01% digitonin) for 15 min on ice, then washed twice with PBSI (PBS with 1× protease inhibitor). Nuclei were counted under a microscope with Hoechst staining. For each sample, 5 × 10^5^ nuclei were combined with 5  × 10^2 ^*Drosophila* S2 cells (as spike-in control). Nuclei-spike-in mixtures were resuspended in 50 µL MED Buffer #1 (20 mM HEPES, 271 mM NaCl, 2% BSA, 0.5 mM Spermidine, 0.01% Digitonin, 2 mM EDTA, and 1×Protease Inhibitor Cocktail) containing 1 µL Bridged Protein A-Tn5 and 2 µg H4K16ac antibody (Abcam ab109463), and incubated overnight at 4 °C. Nuclei were washed in MED Buffer #2 (MED Buffer #1 without EDTA), incubated in 10 mM MgCl_2_, at 550 rpm for 1 h at 37 °C for tagmentation, and then terminated by adding 10 mM EDTA. The nuclei were lysed in 500 mM NaCl, 1% SDS and protease K at 55 °C, 850 rpm for at least 2 h, followed by column purification of DNA. Libraries were prepared using NEBNext^®^ High-Fidelity 2X PCR Master Mix and Nextera N5/N7 primers for PCR amplification according to the recommended cycle number, followed by post-PCR clean-up with SPRIselect Beads and fragments distribution analysis. Biological duplicates per group were prepared for CUT&Tag and sent for sequencing on Illumina NovaSeq 6000.

### CUT&Tag data processing

The Illumina adapters and low-complexity adapters were removed from the reads by Trim Galore (version 0.6.6) (https://www.bioinformatics.babraham.ac.uk/projects/trim_galore/), cutadapt (version 4.2) (Kechin et al, 2017). The reads were mapped to the *C. elegan*s genome ce11 by bowtie2 (version 2.4.1) (Langmead and Salzberg, 2012) with option “-X 2000”. The unmapped reads were further mapped to the *Drosophila* genome dm6 by bowtie2 (version 2.4.1) with option “-X 2000”. PCR duplicates were removed by sambamba (version 0.8.2)(Tarasov et al, 2015) with option “--overflow-list-size 600000”. Alignments were further filtered by sambamba (version 0.8.2) with the option “-F 1804”. NormalizeFactor was calculated as: 10000/(number of alignments mapped to spike-in genome). The average NormalizeFactor of two biological replicates was used for all the following analyses. RPKM normalization was finished by bamCoverage command implemented in deepTools (version 3.5.3) (Ramírez et al, 2014) with option “--scaleFactor NormalizeFactor -bs 50”. Peak intensity lineplot and heatmap were generated using the computeMatrix command implemented in deepTools (v3.5.3). Genomic tracks were visualized using the Integrative Genomics Viewer (IGV) (Robinson et al, 2011). Peaks against background were identified by MACS3 (version 3.0.1)(Zhang et al, 2008) for each sample. Peaks from all samples were merged into one union peak with bedtools merge (version 2.30.0) (Quinlan and Hall, 2010) command if needed. Union peaks were annotated by annotatedPeaks.pl script implemented in HOMER (Heinz et al, 2010). Sample by union peak count table were generated by featureCounts (version 2.0.1) (Liao et al, 2014). Differential peaks were identified by edgeR (version 3.40.2) (Robinson et al, 2010) package implemented in R (version 4.2.1) (https://www.r-project.org/) with 1/NormalizeFactor used for TMM normalization. glmQLFit and glmQLFTest were used for significance testing. Peaks with FDR <0.01 were considered as differential peaks.

### RNA extraction and quantitative RT-PCR

Total RNA was extracted with RNAzol (GeneCopoeia) following the manufacturer’s instructions. gDNA was removed with DNase before reverse transcription with the 5 x Hiscript ® III QRT SuperMix (Vazyme). Quantitative PCR was performed with SYBR Green PCR reagent (Vazyme) on a quantitative PCR system (Jena Qtower 3G). Relative expression levels of the interested genes were normalized to *act-1*.

### Western blot analysis

Worms at indicated stages were pelleted and snap-frozen in liquid nitrogen. Pellets were resuspended in 100 µL 1% SDS, sonicated (3 × 10 s pulses, 30% amplitude), and boiled at 95 °C for 5 min. Lysates were centrifuged (16,000×*g*, 10 min, 4 °C) for supernatant collection. Protein concentration was determined using the BCA assay (Pierce 23225) against BSA standards. Equivalent protein amounts (20 µg/lane) were separated on SDS-PAGE gels and transferred to a nitrocellulose membrane. Membranes were blocked in 5% (w/v) non-fat milk prepared with Tris buffered saline with Tween-20 (TBST) for 1 h at room temperature, then incubated overnight at 4 °C with primary antibodies: FLAG (HUABIO M1403-2, 1:2000), H4K16ac (Abcam ab109463, 1:1000), H4 (CST 2592, 1:1000), H3K9ac (CST 9649), H3K27ac (Abcam ab177178), H3 (Abcam ab1791) and Tubulin (Abcam ab6160, 1:1000). After washes with TBST, membranes were incubated with HRP-conjugated secondary antibodies (1:5000) for 1 h at room temperature. Proteins were detected using ECL reagent (Pierce) and imaged on an AI680 gel documentation system (GE Healthcare). Band intensities were analyzed using ImageJ. Target protein levels were normalized first to loading controls, then to relevant experimental controls.

### Native-PAGE and filter-trap assay for protein aggregation analysis

To analyze protein aggregation states, worm pellets were lysed in native lysis buffer (50 mM Tris, pH 7.4, NaCl 100 mM, 5 mM MgCl_2_, 0.5% Triton X-100, Complete protease inhibitor (Roche)), mechanically disrupted, and centrifuged at 300×*g* for 1 min to remove large debris. Protein concentration of the supernatant was determined by the BCA assay. For Native-PAGE, equal amounts of protein (20 µg) were loaded onto BeyoGel™ Blue Native-PAGE gels (Beyotime P0545S) and electrophoresed according to the manufacturer’s instructions. Proteins were then transferred to PVDF membranes for immunoblotting as described above, using antibodies against Aβ (BioLegend 803014) or GFP (CST 2956). For the filter-trap assay, 100 µg of native lysate protein was supplemented with SDS to a final concentration of 0.5%, loaded onto a 0.22 µm cellulose acetate membrane assembled in a Bio-Dot Apparatus (Bio-Rad, 1706545), and filtered under gentle vacuum. The membrane was then washed with 0.2% SDS, and the retained SDS-insoluble aggregates were detected by immunoblotting for Aβ or GFP.

### Fatty acid composition analysis

The frozen worm pellet with ~50 µL was resuspended with 3 mL chloroform: methanol (2:1) in a glass tube for 1 h at room temperature, then mixed with 0.4 mL of 0.9% NaCl, vortexed, and centrifuged (2000 rpm for 5 min). The bottom organic phase (2 mL) was collected and dried under nitrogen flow. The extracted total lipids were derivatized in 700 µL 14% BF_3_ methanol at 75 °C for 45 min to generate fatty acid methyl esters (FAMEs). FAMEs were extracted in hexane and analyzed by GC-MS (Agilent 5977B) using an HP-88 capillary column (60 m × 0.25 mm × 0.2 µm; Agilent 112-8867) with helium carrier gas (1 mL/min). Relative amounts of fatty acid methyl esters were reported in Dataset EV4.

### Fatty acid supplementation

The fatty acid-supplemented NGM was prepared as previously described (Deline et al, 2013). To ensure fatty acid solubility, 0.1% Tergitol NP-40 (final concentration) was added to both control and supplemented media before autoclaving. A final concentration of 0.8 mM of fatty acid was added to the melted NGM agar at 55 °C before pouring plates. The plates were kept at 4 °C in the dark for no longer than 1 week. HT115 (L4440) bacteria were used for all supplementation and seeded 24–48 h prior to transferring animals.

### Lipidomics analysis

For lipidomic analysis, adult worm pellets were snap-frozen. For total lipid extraction, 1.5 mL of methanol was added to the sample aliquot in a glass tube with a Teflon-lined cap, and the mixture was vortexed. Then, 5 mL of methyl tert-butyl ether (MTBE) was added, and the sample was incubated for 1 h at room temperature with shaking. Phase separation was induced by adding 1.25 mL of MS-grade water. After 10 min of incubation, the sample was centrifuged at 1000×*g* for 10 min. The upper (organic) phase was collected, and the lower phase was re-extracted with 2 mL of a solvent mixture equivalent in composition to the expected upper phase, prepared by mixing MTBE/methanol/water (10:3:2.5, v/v/v) and collecting the upper layer. The combined organic phases were dried under nitrogen flow.

Extracted lipids were dissolved in 200 µL of dichloromethane/methanol (3:1, v/v). Lipidomic profiling was performed on an LC-MS system consisting of an Agilent 1290 Infinity II UHPLC coupled to a Bruker Tims TOF Pro2 mass spectrometer. Chromatographic separation was achieved on an ACQUITY UPLC BEH C18 column (100 mm × 2.1 mm, 1.7 µm). The mobile phase consisted of (A) 10 mM ammonium acetate and 0.2 mM ammonium fluoride in 9:1 water/methanol, and (B) 10 mM ammonium acetate and 0.2 mM ammonium fluoride in 2:3:5 acetonitrile/methanol/isopropanol, delivered at a flow rate of 0.30 mL/min. The elution gradient was as follows: 70% B for 1 min, linear increase to 86% B over 2.5 min, hold at 86% B for 6.5 min, linear increase to 100% B over 1 min, and hold at 100% B for 6 min. The injection volume was 2 µL. MS data were acquired using electrospray ionization in both positive and negative ion modes over a mass range of 100–1350 m/z. TIMS settings included: 1/k0 range 0.1–1.5 V·s/cm², ramp time 100 ms, and accumulation time 100 ms. Additional MS parameters were: capillary voltage 4500 V, nebulizer 2 bar, dry gas flow 8 L/min, dry gas temperature 230 °C, sheath gas temperature 400 °C, and sheath gas flow 4 L/min.

### Fixed Nile Red staining

Lipid storage was analyzed using Nile Red staining following the previously described protocol (Pino et al, 2013). Adult worms were collected and fixed with 40% isopropanol for 5 min and stained with Nile Red (3 µg/mL) dissolved in 40% isopropanol for 2 h at room temperature in the dark. Worms were rinsed with M9 buffer and then placed on 2% agarose pads for observation. Images were acquired at 5× magnification on a Leica DM500 Microscope with fixed exposure parameters (*n* > 50 animals/group). The relative fat level was analyzed by ImageJ and normalized to the body area of the individual animals.

### Quantification and statistical analysis

GraphPad Prism 8.0 (GraphPad Software, Inc.) was used for statistical analyses in this study. Statistical information, including animal number, error bars, *p* values, statistical test used, and biological replicates, can be found in the Figures, Figure legends and Dataset EV6. Animals used for experiments were randomly chosen from age-synchronized populations. No data in this study were excluded. Blinding of investigators was not performed in most experiments due to the need to visually identify different genotypes and treatment conditions. Data from paralysis assays and lifespan assays were analyzed using OASIS 2 (Han et al, 2024) with the log-rank (Mantel–Cox) test. Differences between the two conditions were analyzed by a two-tailed unpaired *t*-test. Differences between multiple groups with two variations were analyzed by two-way ANOVA.

## Supplementary information

Peer Review File

Dataset EV1

Dataset EV2

Dataset EV3

Dataset EV4

Dataset EV5

Dataset EV6

Source data Fig. 1

Source data Fig. 2

Source data Fig. 3

Source data Fig. 4

Source data Fig. 5

Source data Fig. 6

Source data Fig. 7

EV Figure Source Data

Expanded View Figures

## Data Availability

RNA-seq and CUT&Tag data were deposited in the Gene Expression Omnibus (GSE300492 and GSE300494). Any additional information required to reanalyze the data reported in this paper is available from the lead contact upon reasonable request. Further information, resources and reagents are available from the Lead Contact, Lianfeng Wu (wulianfeng@westlake.edu.cn), upon reasonable request. The source data of this paper are collected in the following database record: biostudies:S-SCDT-10_1038-S44318-026-00851-8.
