# Supplementary material for: Developmental priming of adult proteostasis and longevity by NuA4 complex activity in early life
Source: EMBO J. 2026 Jul 16;45(16):5689–726. doi: 10.1038/s44318-026-00851-8 (PMC13482575; doi:10.1038/s44318-026-00851-8)
Supplement: Supplementary file 1 [file 44318_2026_851_MOESM16_ESM.pdf]

Expanded View Figures

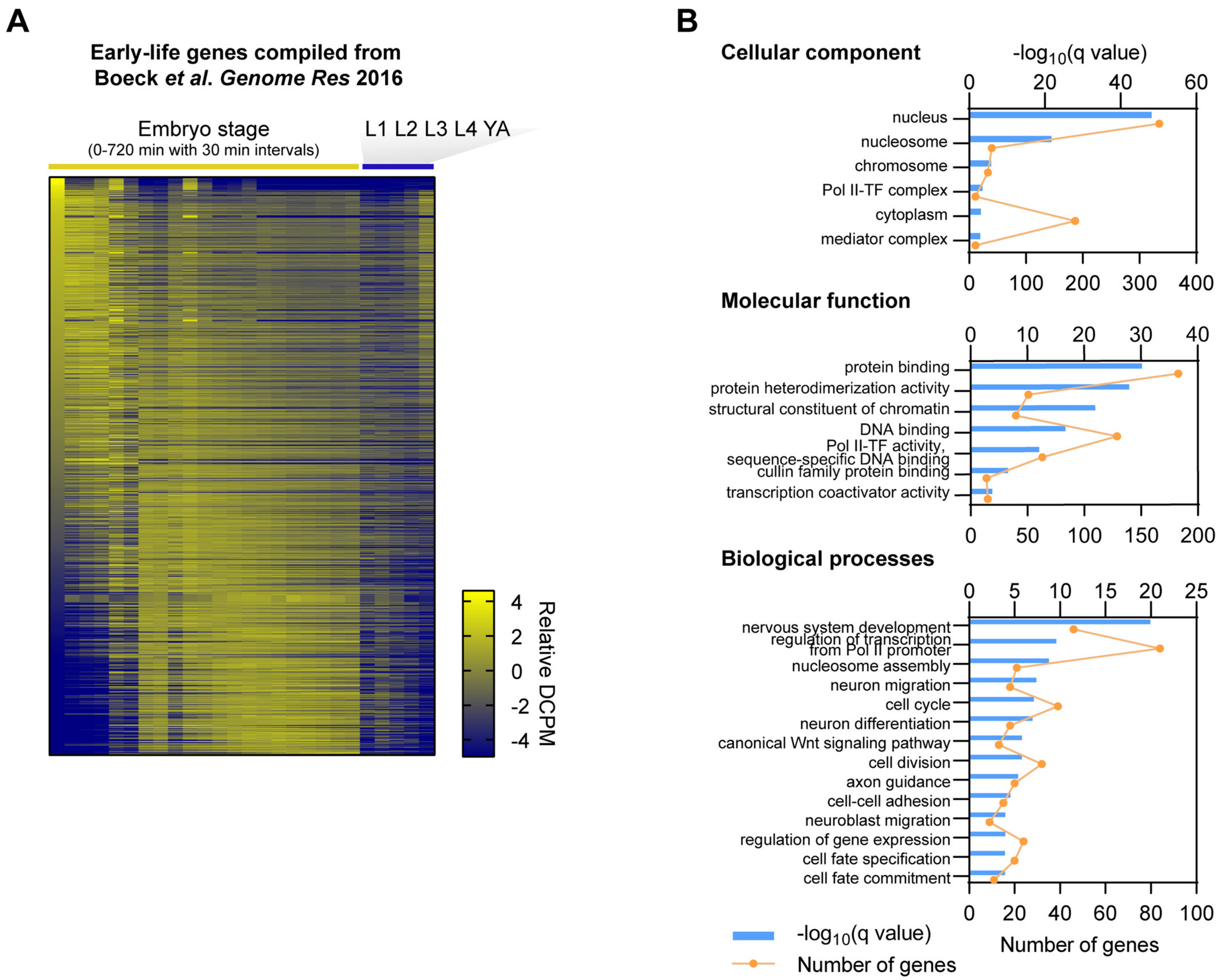

Figure EV1. Expression pattern and functional analysis of genes selected for early-life RNAi screen.

Related to Fig. 1. (**A**) Heatmap of relative expression levels of the early-life genes. (**B**) GO enrichment analysis of the early-life genes. Source data are available online for this figure.

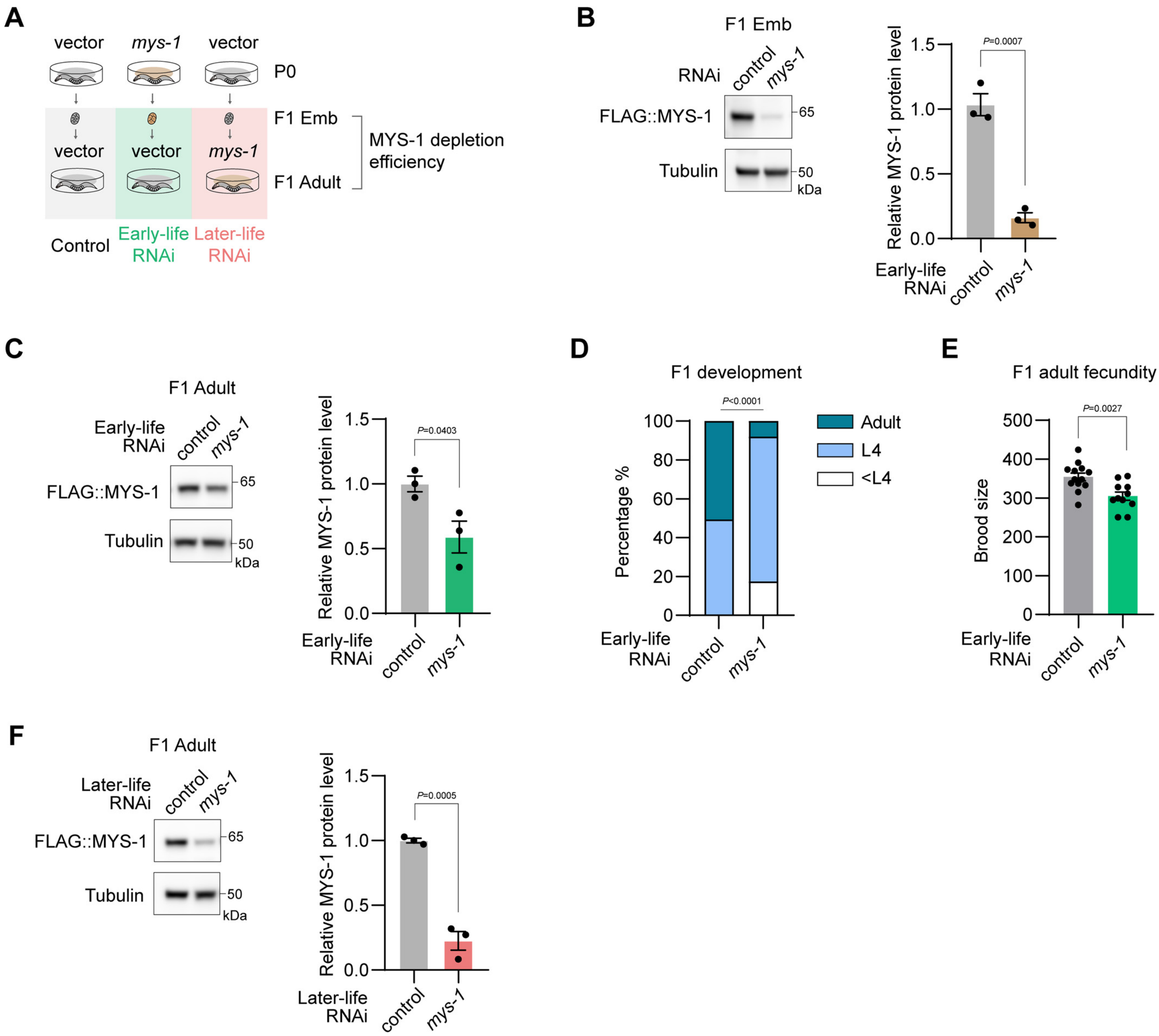

Figure EV2. Detection of MYS-1 protein levels following early- and later-life RNAi treatments.

Related to Fig. 1. (**A**) Experimental illustration for assessing MYS-1 depletion efficiency following early-life or later-life *mys-1* RNAi. (**B**, **C**) Western blot analysis of MYS-1 protein levels in F1 animals in embryonic (**B**) and adulthood stages (**C**) following early-life *mys-1* RNAi treatment. Results are average ± SEM of three independent experiments. Statistical significance was assessed by a two-tailed unpaired *t*-test. (**D**) Developmental stage quantification under early-life *mys-1* RNAi. Results are a composite of three independent experiments. Animal number = 217 (control), 126 (*mys-1*). Statistical significance was assessed by the chi-squared test. (**E**) Brood size quantification under early-life *mys-1* RNAi. Results are representative of three independent experiments. Data were average ± SEM. Animal number = 13 (control), 11 (*mys-1*). Statistical significance was assessed by a two-tailed unpaired *t*-test. (**F**) Western blot analysis of MYS-1 protein levels in F1 adults following later-life *mys-1* RNAi treatment. Results are average ± SEM of three independent experiments. Statistical significance was assessed by a two-tailed unpaired *t*-test. Source data are available online for this figure.

© The Author(s)

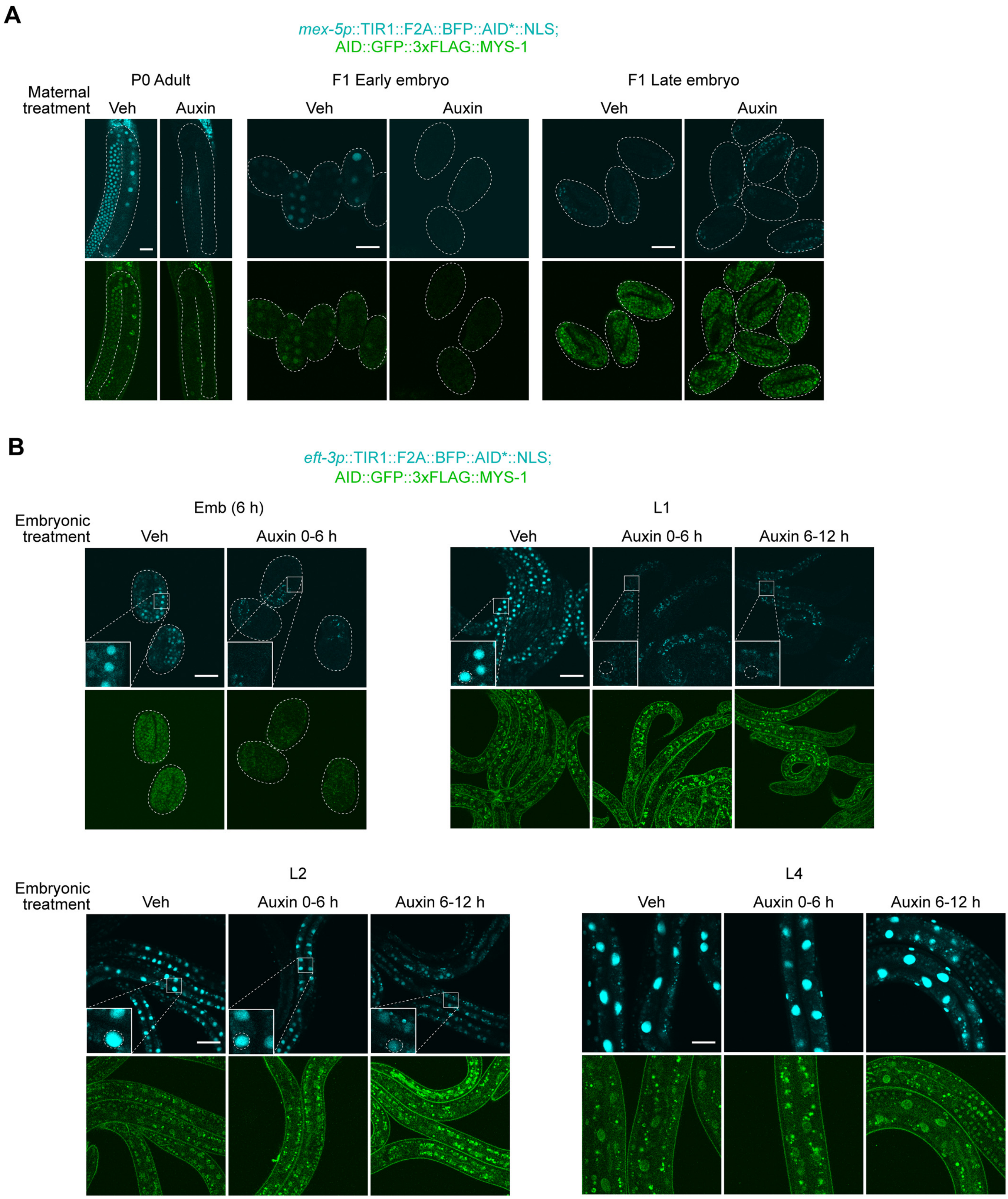

Figure EV3. Validation of temporal removal of MYS-1 in AID systems.

Related to Fig. 1. (**A**) Representative images of *mex-5p*::BFP and GFP::MYS-1 expression in animals at the indicated stages after maternal auxin treatment (K-NAA 4 mM). Scale bar: 20 µm. (**B**) Representative images of *eft-3p*::BFP and GFP::MYS-1 expression in animals at the indicated stages after embryonic auxin treatment (IAA-AM 1 mM). Scale bar: 20 µm. Results are representative of three independent experiments. Animal number >20 per group per experiment. Source data are available online for this figure.

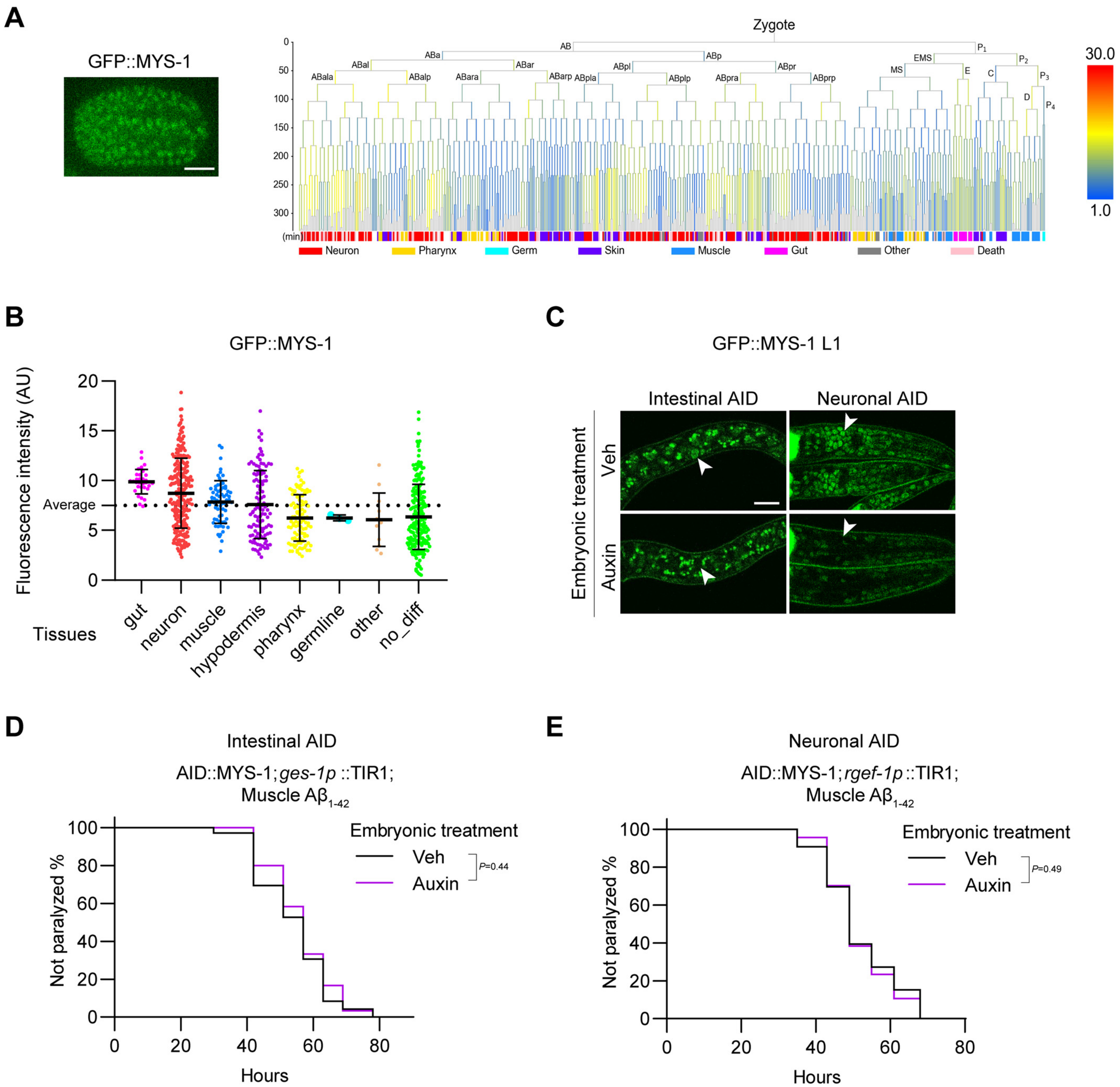

Figure EV4. Tissue-specificity analysis of early-life MYS-1 expression and function.

Related to Fig. 1. (**A**) Representative image of GFP::MYS-1 in embryos (left) and tree visualization of MYS-1 expression mapped on the embryonic cell lineage (right). Scale bar: 10 μm. (**B**) Quantification of MYS-1 expression in the indicated tissue type. Data were average ± SD. Each dot represents one cell. Results are representative of five embryos traced and analyzed, yielding similar results. (**C**) Representative image of GFP::MYS-1 L1 animals with intestine and neuron-specific degradation in the embryo-to-L1 stage. Arrowheads indicate the GFP::MYS-1 signal in the nucleus of intestine cells and head neurons. Scale bar: 10 μm. Results are representative of three independent experiments. Animal number >20 per group per experiment. (**D**, **E**) Paralysis analysis of the muscle $A\beta_{1-42}$ animals with intestinal (**D**) and neuronal (**E**) specific AID in early life. Results are representative of three independent experiments. Statistical significance was assessed by the log-rank test. Source data are available online for this figure.

 © The Author(s)

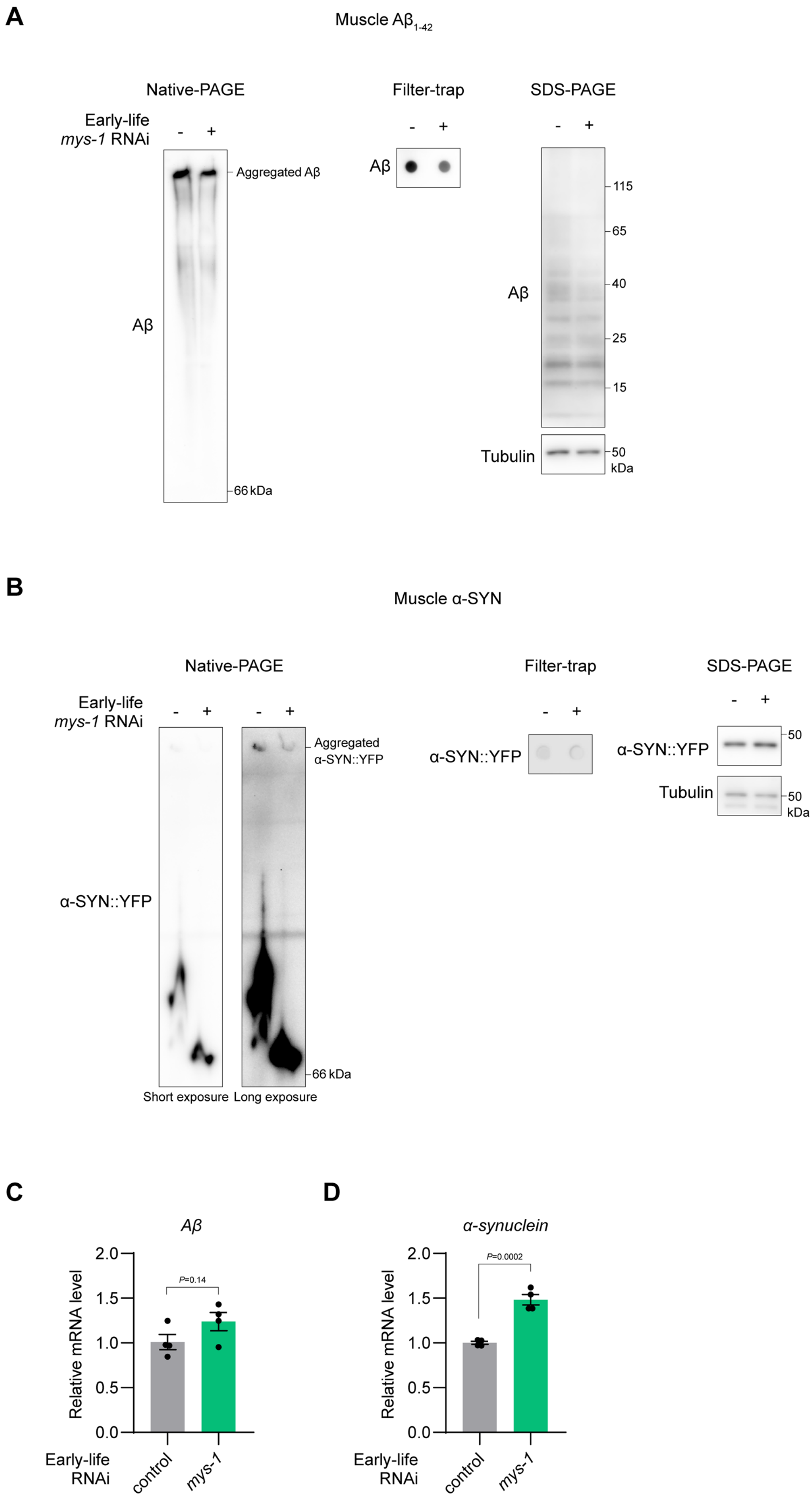
A
Muscle Aβ1-42
Native-PAGE
Filter-trap
SDS-PAGE
Early-life
mys-1 RNAi
- +
Aggregated Aβ
Aβ
66 kDa
115
65
40
25
15
Tubulin
50
kDa
B
Muscle α-SYN
Native-PAGE
Filter-trap
SDS-PAGE
Aggregated
α-SYN::YFP
α-SYN::YFP
Tubulin
66 kDa
Short exposure
Long exposure
C
Aβ
Relative mRNA level
2.0
1.5
1.0
0.5
0.0
P=0.14
Early-life
RNAi
control
mys-1
D
α-synuclein
P=0.0002

Figure EV5. Early-life *mys-1* RNAi reduces protein aggregates in late life.

Related to Fig. 2. **A**, **B** Protein aggregates analysis of Aβ and α-synuclein in adult animals under early-life *mys-1* RNAi with native Western blot, filter trap, and denaturing Western blot. Results are representative of three independent experiments. (**C**, **D**) Relative mRNA level of *Aβ* and *α-synuclein* in adult animals under early-life *mys-1* RNAi. Results are average ± SEM of three independent replicates. Statistical significance was assessed by a two-tailed unpaired *t*-test. Source data are available online for this figure.

 © The Author(s)

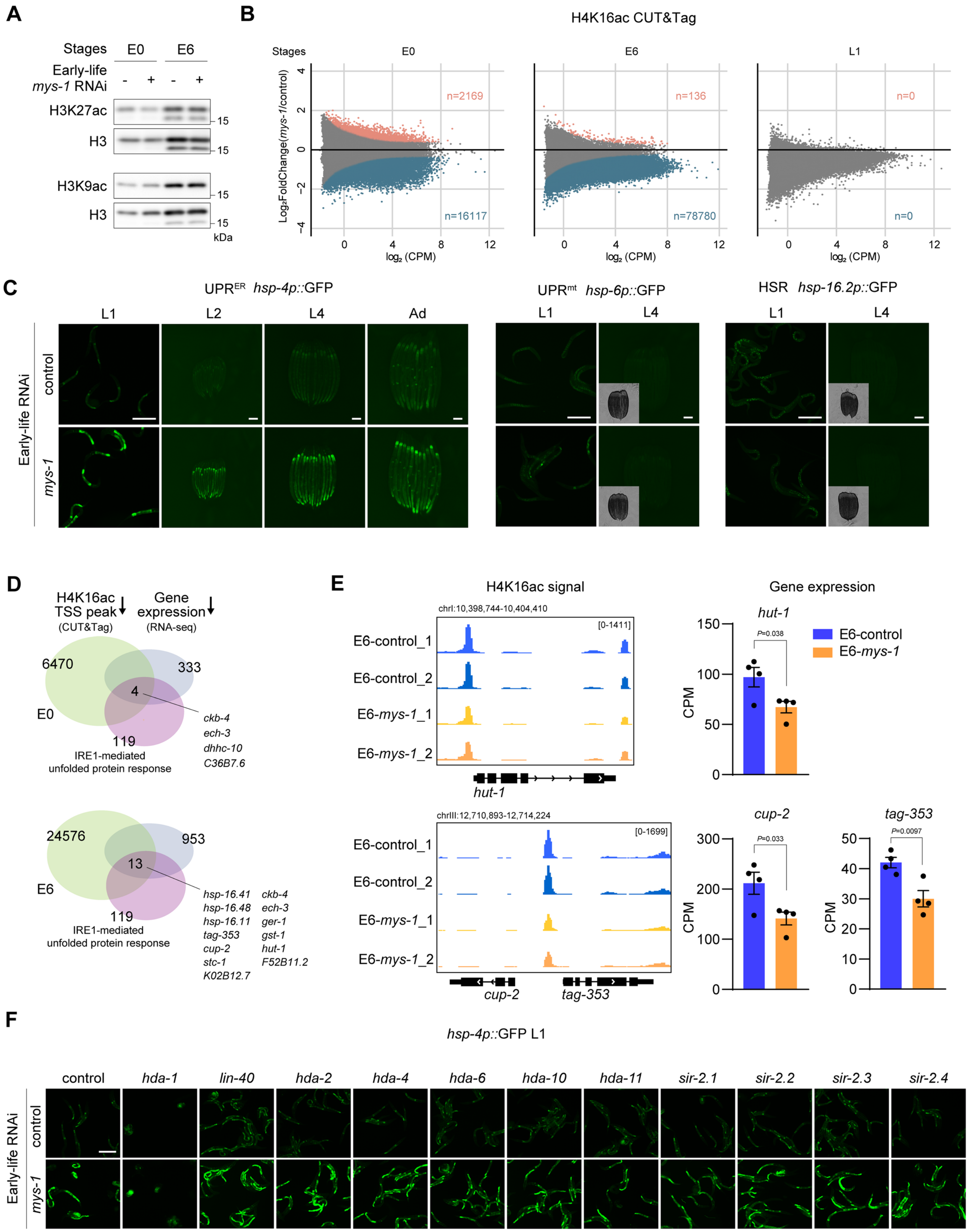
A
Stages
E0
E6
Early-life
mys-1 RNAi
H3K27ac
H3
H3K9ac
H3
15
kDa
B
H4K16ac CUT&Tag
Stages
E0
E6
L1
Log2FoldChange(mys-1/control)
n=2169
n=16117
n=136
n=78780
n=0
n=0
log2 (CPM)
C
UPRER hsp-4p::GFP
UPRmt hsp-6p::GFP
HSR hsp-16.2p::GFP
L1
L2
L4
Ad
Early-life RNAi
control
mys-1
D
H4K16ac TSS peak
(CUT&Tag)
Gene expression
(RNA-seq)
6470
333
4
E0
119
IRE1-mediated unfolded protein response
ckb-4
ech-3
dhhc-10
C36B7.6
24576
953
13
E6
hsp-16.41
hsp-16.48
hsp-16.11
tag-353
cup-2
stc-1
K02B12.7
ckb-4
ech-3
ger-1
gst-1
hut-1
F52B11.2
E
H4K16ac signal
Gene expression
chrI:10,398,744-10,404,410
[0-1411]
E6-control_1
E6-control_2
E6-mys-1_1
E6-mys-1_2
hut-1
P=0.038
E6-control
E6-mys-1
CPM
chrIII:12,710,893-12,714,224
[0-1699]
cup-2
tag-353
P=0.033
P=0.0097
F
hsp-4p::GFP L1
control
hda-1
lin-40
hda-2
hda-4
hda-6
hda-10
hda-11
sir-2.1
sir-2.2
sir-2.3
sir-2.4
Early-life RNAi
control
mys-1

Figure EV6. Epigenomic and transcriptomic analysis of genes affected by early-life *mys-1* RNAi.

Relate to Fig. 3. (**A**) Western blot analysis of H3K9ac and H3K27ac levels in animals at the indicated stages following early-life *mys-1* RNAi treatment. Results are representative of three independent experiments. (**B**) Volcano plot of differential H4K16ac peaks at indicated stages following early-life *mys-1* RNAi treatment. (**C**) Representative images of the *hsp-4p::*GFP, *hsp-6p::*GFP and *hsp-16.2p::*GFP animals in the indicated stage following early-life RNAi of *mys-1*. Scale bar: 100 µm. Results are representative of three independent experiments. Animal number >20 (for L1) or 10 (for L2, L4, and Ad) per group per experiment. Note: The representative control image (L1) is the same as that shown in Fig. 5C, as both experiments utilized identical control conditions. (**D**) Venn diagram of the IRE1-UPR^{ER} pathway genes that were downregulated on both H4K16ac peaks (at TSS regions) and transcription levels following early-life *mys-1* RNAi treatment. (**E**) The IGV tracks of H4K16ac CUT&Tag analysis (left, two independent replicates) and RNA-seq signals (right, four independent replicates) of the representative genes from (**D**). Data were average ± SEM. Statistical significance was assessed by a two-tailed unpaired *t*-test. (**F**) Representative images of the *hsp-4p::*GFP animals in the L1 stage following early-life RNAi dually targeting *mys-1* and HDAC genes. Scale bar: 100 µm. Results are representative of two independent experiments. Animal number >20 per group per experiment. Source data are available online for this figure.

 © The Author(s)

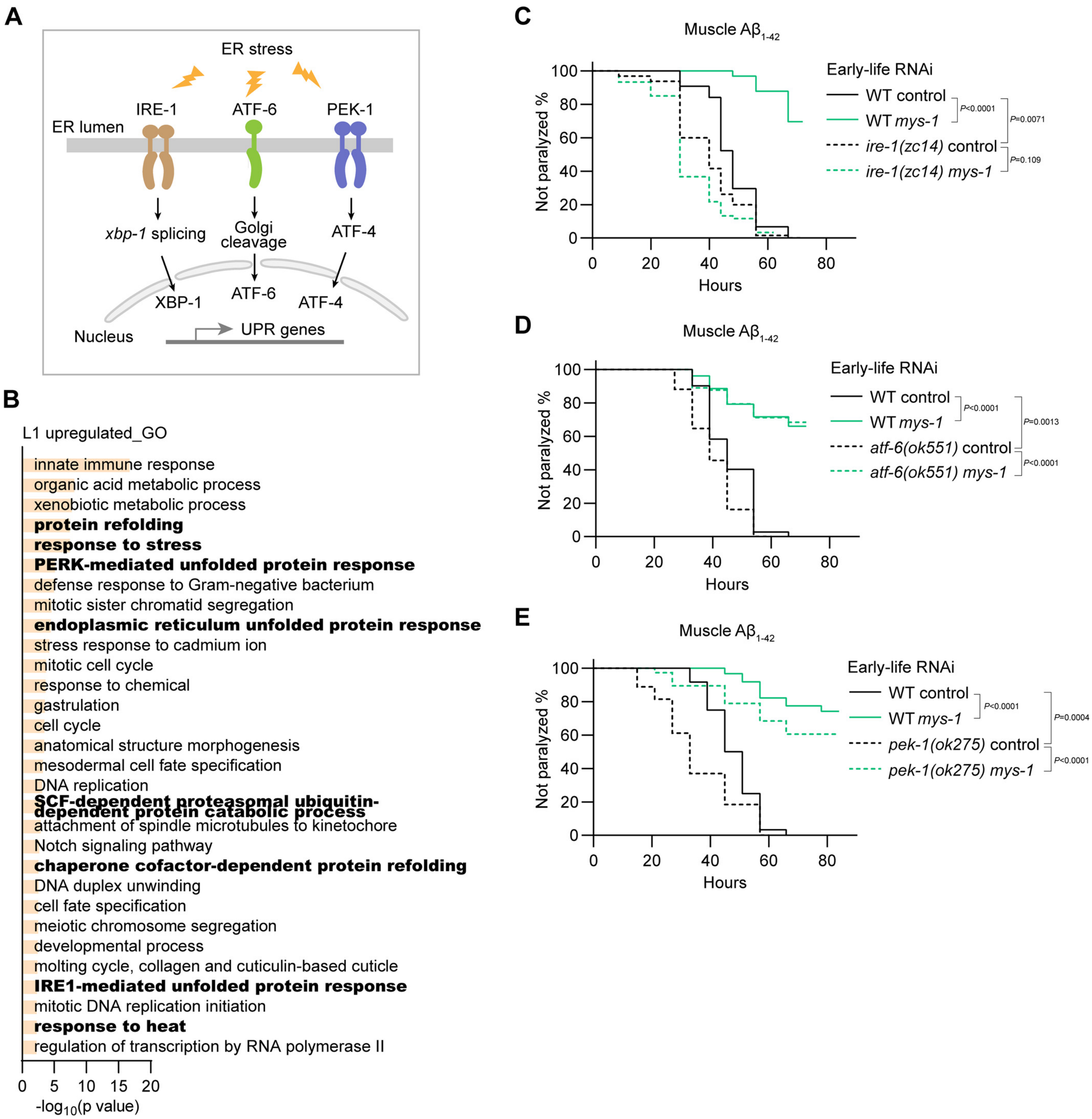

Figure EV7. Early-life NuA4 programs lifelong proteostasis exclusively through the IRE-1–XBP-1 pathway.

Related to Fig. 4. (**A**) Schematic diagram of main ER stress pathways. (**B**) GO enrichment analysis of upregulated genes in L1 animals subjected to early-life *mys-1* RNAi. Terms related to UPR^{ER} are shown in bold. (**C–E**) Paralysis analysis of muscle $A\beta_{1-42}$ animals in WT and *ire-1(zc14)* (**C**), *atf-6(ok551)* (**D**), and *pek-1(ok275)* (**E**) genetic background, following early-life *mys-1* RNAi treatment. Results are representative of three independent experiments. Statistical significance was assessed by the log-rank test. Source data are available online for this figure.

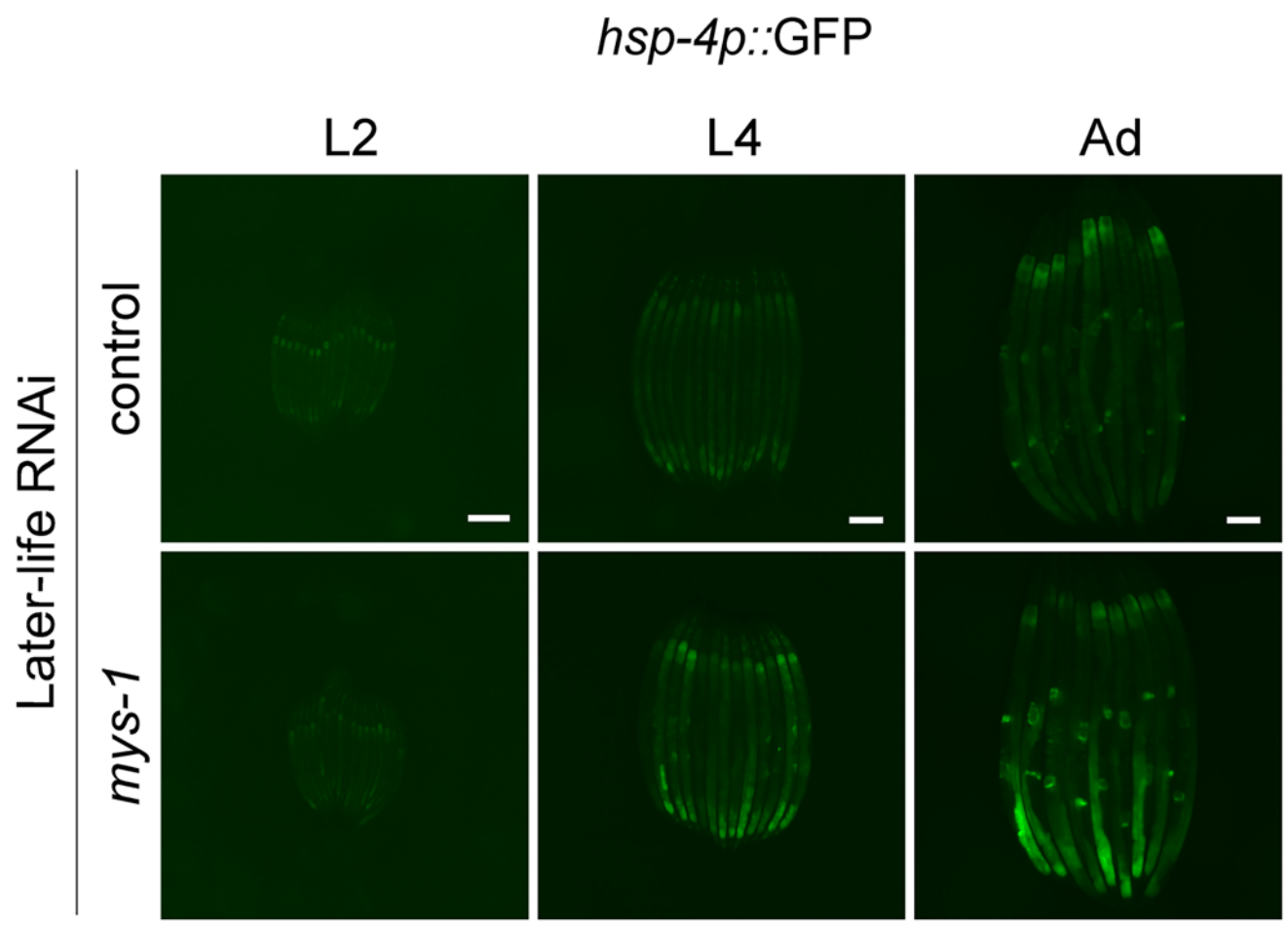

Figure EV8. Later-life *mys-1* RNAi induces an unchanged early larval UPR^{ER} despite an increase in later stages.

Related to Fig. 5. Representative images of *hsp-4p::*GFP animals at the indicated stages following later-life *mys-1* RNAi treatment. Scale bar: 100 μm. Results are representative of three independent experiments. Animal number >10 per group per experiment. Source data are available online for this figure.

 © The Author(s)

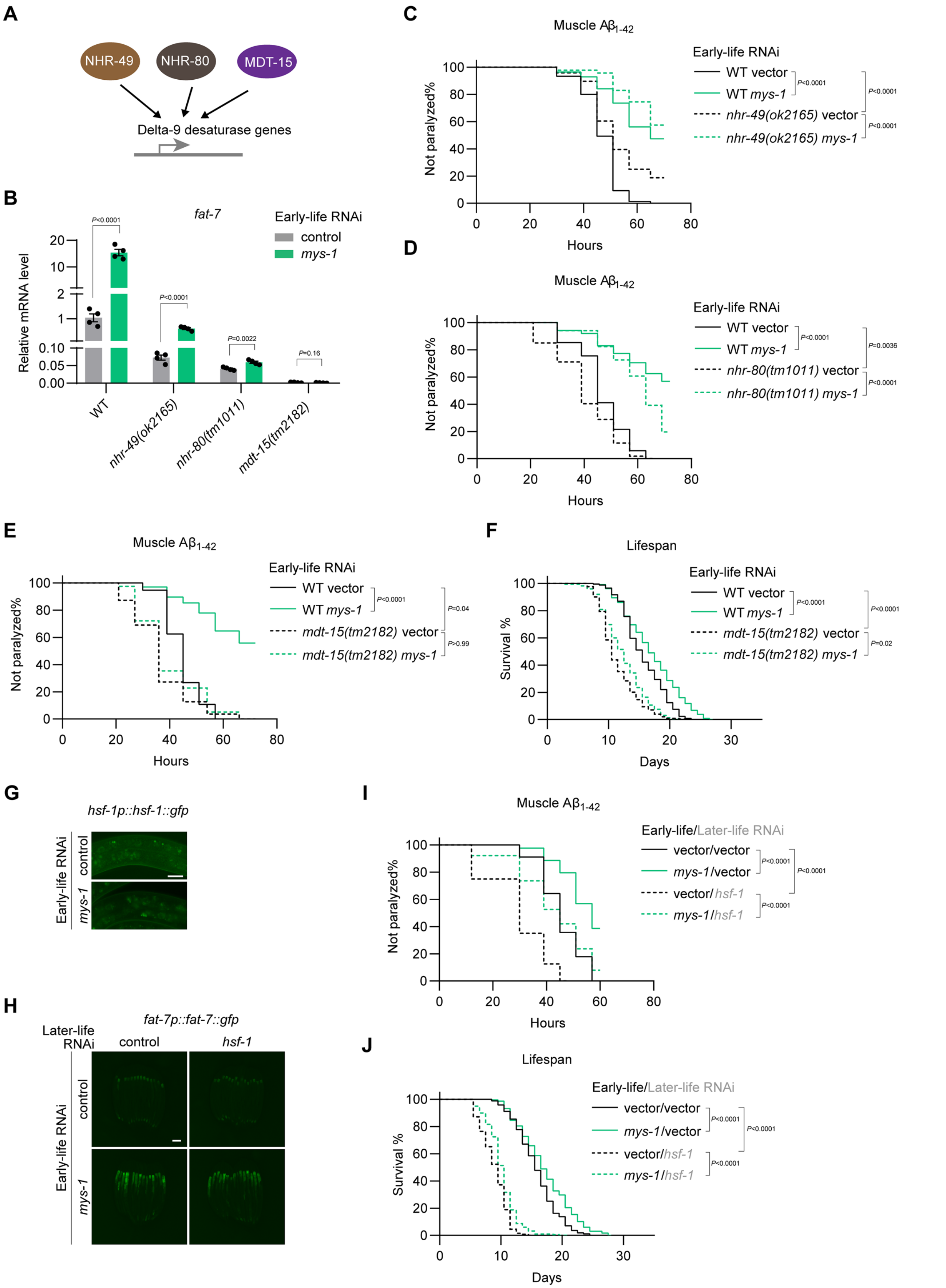
A
NHR-49
NHR-80
MDT-15
Delta-9 desaturase genes
B
fat-7
Early-life RNAi
control
mys-1
Relative mRNA level
WT
nhr-49(ok2165)
nhr-80(tm1011)
mdt-15(tm2182)
P<0.0001
P<0.0001
P=0.0022
P=0.16
C
Muscle Aβ1-42
Early-life RNAi
WT vector
WT mys-1
nhr-49(ok2165) vector
nhr-49(ok2165) mys-1
Not paralyzed%
Hours
D
Muscle Aβ1-42
Early-life RNAi
WT vector
WT mys-1
nhr-80(tm1011) vector
nhr-80(tm1011) mys-1
Not paralyzed%
Hours
E
Muscle Aβ1-42
Early-life RNAi
WT vector
WT mys-1
mdt-15(tm2182) vector
mdt-15(tm2182) mys-1
Not paralyzed%
Hours
F
Lifespan
Early-life RNAi
WT vector
WT mys-1
mdt-15(tm2182) vector
mdt-15(tm2182) mys-1
Survival %
Days
G
hsf-1p::hsf-1::gfp
Early-life RNAi
control
mys-1
H
fat-7p::fat-7::gfp
Later-life RNAi
control
hsf-1
Early-life RNAi
control
mys-1
I
Muscle Aβ1-42
Early-life/Later-life RNAi
vector/vector
mys-1/vector
vector/hsf-1
mys-1/hsf-1
Not paralyzed%
Hours
J
Lifespan
Early-life/Later-life RNAi
vector/vector
mys-1/vector
vector/hsf-1
mys-1/hsf-1
Survival %
Days

Figure EV9. Early-life NuA4 governs lifelong proteostasis and longevity predominantly through the XBP-1-SBP-1/MDT-15-FAT-7 signaling axis.

Related to Fig. 6. (**A**) Schematic diagram of transcriptional regulators of delta-9 desaturase genes. (**B**) Relative mRNA levels of *fat-7* in adult animals with the indicated genotype under early-life *mys-1* RNAi. Results are average ± SEM of three independent replicates. Statistical significance was assessed by a two-tailed unpaired *t*-test with FDR correction. (**C–E**) Paralysis analysis of muscle $A\beta_{1-42}$ animals in WT and *nhr-49(ok2165)* (**C**), *nhr-80(tm1011)* (**D**), and *mdt-15(tm2182)* (**E**) background, following early-life *mys-1* RNAi treatment. Results are representative of three independent experiments. Statistical significance was assessed by the log-rank test. (**F**) Lifespan analysis of WT and *mdt-15(tm2182)* animals under early-life *mys-1* RNAi. Results are a composite of three independent experiments. Statistical significance was assessed by the log-rank test. (**G**) Representative images of *hsf-1p::hsf-1::gfp* animals under early-life *mys-1* RNAi treatment. Scale bar: 20 µm. Results are representative of three independent experiments. Animal number >20 per group per experiment. (**H**) FAT-7 expression of the animals under early-life *mys-1* RNAi treatment and later-life inhibition of *hsf-1*. Scale bar: 100 µm. Results are representative of three independent experiments. Animal number >10 per group per experiment. (**I**, **J**) Paralysis analysis and Lifespan analysis of the animals under early-life *mys-1* RNAi treatment and later-life inhibition of *hsf-1*. Results are representative (**I**) or composite (**J**) of three independent experiments. Statistical significance was assessed by the log-rank test. Source data are available online for this figure.

© The Author(s)

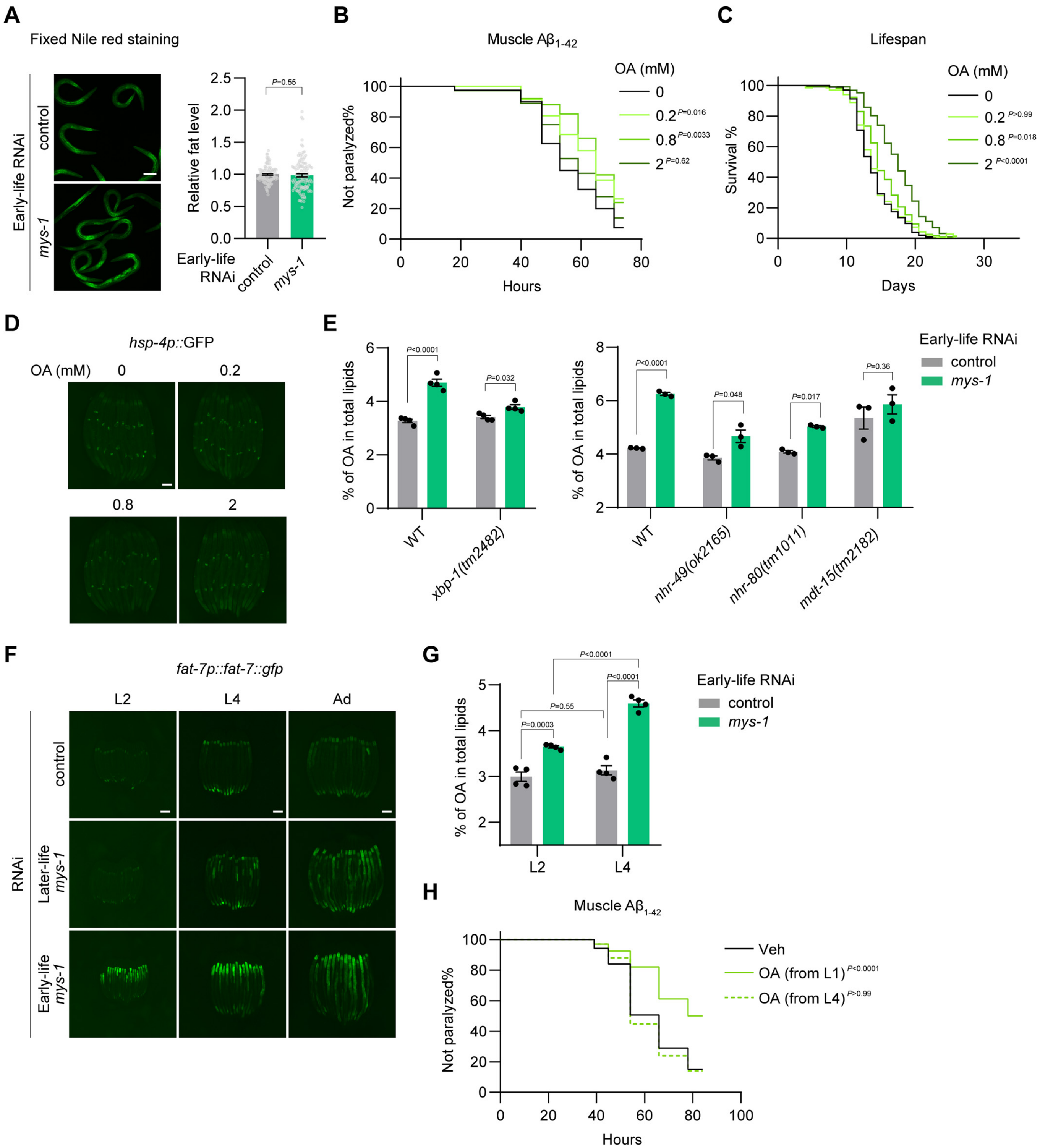
A
Fixed Nile red staining
Early-life RNAi
control
mys-1
Relative fat level
P=0.55
Early-life RNAi
control
mys-1
B
Muscle Aβ1-42
OA (mM)
0
0.2 P=0.016
0.8 P=0.0033
2 P=0.62
Not paralyzed%
Hours
C
Lifespan
OA (mM)
0
0.2 P>0.99
0.8 P=0.018
2 P<0.0001
Survival %
Days
D
hsp-4p::GFP
OA (mM) 0 0.2
0.8 2
E
% of OA in total lipids
P<0.0001
P=0.032
WT
xbp-1(tm2482)
P<0.0001
P=0.048
P=0.017
P=0.36
WT
nhr-49(ok2165)
nhr-80(tm1011)
mdt-15(tm2182)
Early-life RNAi
control
mys-1
F
fat-7p::fat-7::gfp
L2 L4 Ad
RNAi
control
Later-life mys-1
Early-life mys-1
G
% of OA in total lipids
P<0.0001
P<0.0001
P=0.55
P=0.0003
L2 L4
Early-life RNAi
control
mys-1
H
Muscle Aβ1-42
Veh
OA (from L1) P<0.0001
OA (from L4) P>0.99
Not paralyzed%
Hours

Figure EV10. Developmentally accumulated oleic acid establishes adult proteostatic resilience.

Related to Fig. 7. (**A**) Relative fat levels of animals subjected to early-life *mys-1* RNAi analyzed by Nile Red staining. Scale bar: 100 µm. Results are average ± SEM of three independent experiments. Animal number = 113 (control), 120 (*mys-1*). Statistical significance was assessed by a two-tailed unpaired *t*-test. (**B**, **C**) Paralysis analysis of muscle Aβ_{1-42} animals (**B**) and lifespan analysis of WT animals (**C**) with treatment of the indicated dose of OA. Results are representative (**B**) or composite (**C**) of three independent experiments. Statistical significance was assessed by the log-rank test. (**D**) Representative images of *hsp-4p*::GFP animals with treatment of the indicated dose of OA. Scale bar: 100 µm. Results are representative of three independent experiments. Animal number >10 per group per experiment. (**E**) Relative OA levels to the total fatty acids of animals with the indicated genetic background following early-life *mys-1* RNAi treatment. Results are average ± SEM of three or four independent replicates. Statistical significance was assessed by two-way ANOVA with multiple comparisons. (**F**) Representative images of FAT-7::GFP expression at the indicated stages following later-life and early-life *mys-1* RNAi. Scale bar: 100 µm. Results are representative of three independent experiments. Animal number >10 per group per experiment. (**G**) Relative OA levels to the total fatty acids of animals at the indicated stages following early-life *mys-1* RNAi treatment. Results are average ± SEM of four independent replicates. Statistical significance was assessed by two-way ANOVA with multiple comparisons. (**H**) Paralysis analysis of muscle Aβ_{1-42} animals treated with oleic acid (0.8 mM) from the L1 or L4 stage. Results are representative of three independent experiments. Statistical significance was assessed by the log-rank test. Source data are available online for this figure.

 © The Author(s)
