# Supplementary material for: Developmental priming of adult proteostasis and longevity by NuA4 complex activity in early life
Source: EMBO J. 2026 Jul 16;45(16):5689–726. doi: 10.1038/s44318-026-00851-8 (PMC13482575; doi:10.1038/s44318-026-00851-8)
Supplement: Supplementary file 2 [file 44318_2026_851_MOESM1_ESM.pdf]

THE EMBO JOURNAL

Developmental priming of adult proteostasis and longevity by NuA4 complex activity in early life

Yihan Wang, Xiong Xiong, Runshuai Zhang, Long Xiao, Xinyu Ruan, Tianyi Ni, Zicheng Liu, Jie Chen, Shenlu Qin, Zhuo Du, Yanxiao Zhang, and Lianfeng Wu

Corresponding author(s): Lianfeng Wu (wulianfeng@westlake.edu.cn)

Review Timeline:

Submission Date:	17th Sep 25
Editorial Decision:	17th Dec 25
Revision Received:	17th Mar 26
Editorial Decision:	7th May 26
Revision Received:	19th May 26
Accepted:	10th Jun 26

Editor: Cornelius Schneider

Transaction Report:

(Note: With the exception of the correction of typographical or spelling errors that could be a source of ambiguity, letters and reports are not edited. Depending on transfer agreements, referee reports obtained elsewhere may or may not be included in this compilation. Referee reports are anonymous unless the Referee chooses to sign their reports.)

1st Editorial Decision **17th Dec 2025**

Dear Dr. Wu,

Thank you for submitting your manuscript for consideration by the EMBO Journal. It has now been seen by two referees whose comments are shown below.

Thank you also for providing the detailed and through revision plan. Given the referees' positive recommendations and your willingness to perform major revisions, I would like to invite you to submit a revised version of the manuscript, addressing the comments of all three reviewers as summarized in your revision plan. I should add that it is EMBO Journal policy to allow only a single round of revision, and acceptance of your manuscript will therefore depend on the completeness of your responses in this revised version.

When preparing your letter of response to the referees' comments, please bear in mind that this will form part of the Review Process File, and will therefore be available online to the community. For more details on our Transparent Editorial Process, please review our Editorial Policies: https://link.springer.com/partners/embo-press/editorial-policies

We generally allow three months as standard revision time. As a matter of policy, competing manuscripts published during this period will not negatively impact on our assessment of the conceptual advance presented by your study. However, we request that you contact the editor as soon as possible upon publication of any related work, to discuss how to proceed. Should you foresee a problem in meeting this three-month deadline, please let us know in advance and we may be able to grant an extension.

Thank you for the opportunity to consider your work for publication. I look forward to your revision.

Yours sincerely,

Cornelius Schneider, PhD
Editor
The EMBO Journal
c.schneider@embojournal.org

Instructions for preparing your revised manuscript:

Read our guidance for manuscript revisions and related editorial policies: https://link.springer.com/journal/44318/submission-guidelines#cms-Revised-submissions

Please make sure you upload a letter of response to the referees' comments together with the revised manuscript.

Please also check that the title and abstract of the manuscript are brief, yet explicit, even to non-specialists.

When assembling figures, please refer to our figure preparation guideline in order to ensure proper formatting and readability in print as well as on screen:
https://media.springernature.com/original/springer-cms/rest/v1/content/27825798/data/v1

At EMBO Press we ask authors to provide source data for the main manuscript figures. You will receive a separate email with instructions for providing source data with your revised manuscript, including how to upload and organize the files.

IMPORTANT: When you send the revision we will require
- a point-by-point response to the referees' comments, with a detailed description of the changes made (as a word file).
- a word file of the manuscript text.
- individual production quality figure files (one file per figure)
- a complete author checklist
- Expanded View files (replacing Supplementary Information)
- a Reagents and Tools Table as part of the Methods section

Please remember: Digital image enhancement is acceptable practice, as long as it accurately represents the original data and conforms to community standards. If a figure has been subjected to significant electronic manipulation, this must be noted in the figure legend or in the 'Materials and Methods' section. The editors reserve the right to request original versions of figures and the original images that were used to assemble the figure.

We realize that it is difficult to revise to a specific deadline. In the interest of protecting the conceptual advance provided by the work, we recommend a revision within 3 months (17th Mar 2026). Please discuss the revision progress ahead of this time with the editor if you require more time to complete the revisions. Use the link below to submit your revision:

https://emboj.msubmit.net/cgi-bin/main.plex

Referee #2:

Health and longevity are tightly governed by a complex interplay of genetic and environmental factors; however, the underlying mechanisms are not fully clear. In this manuscript from the Wu group, they present a surprising and intriguing finding that proteostasis and lifespan of adult worms are predetermined by the activity of a MYST-family acetyltransferase (MYS-1) in early developmental stage. Through a combination of classical genetical manipulation strategies and deep-sequencing data analysis, they illustrated that deficiency of MYS-1 from embryo to L1 stage extended lifespan and conferred resistance to proteotoxicity in later-life via a chromatin-ER-lipid axis (or NuA4-H4K16ac-XBP-1-SBP-1-FAT-7 axis). This study clearly demonstrated that longevity and health are not only preprogrammed but also temporally regulated by the activity of specific genetic factors, making this manuscript interesting to many potential readers of EMBO J. I am thus happy to support its publication at EMBO J. Nevertheless, I have several comments that I hope the authors will find helpful to the paper if successfully addressed.

1) Is there any tissue specificity for MYS-1 to regulate late-life proteostasis and longevity?

2) Do the authors know the mechanisms by which XBP-1 regulates the subcellular location or activity of SBP-1?

3) What are the consequences of increasing the activity or expression of MYS-1 during early-life? Conversely, are there any detrimental effects from reducing its activity at this stage?

4) Why is the embryo to L1 stage so critical for MYS-1 to induce adult proteotoxicity resistance and lifespan-extension?

5) Do the authors know how oleic acid, produced by FAT-7, protect worms from proteotoxicity induced by Aβ1-42 in muscles?

Referee #3:

This manuscript identifies a developmental "chromatin-ER-lipid" axis in C. elegans: transient early-life reduction of NuA4 (mys-1) activity lowers H4K16ac, induces an XBP-1-dependent UPRER, remodels ER, activates SBP-1→FAT-5/7, elevates oleic acid (OA), and yields durable proteostasis benefits and lifespan extension. The story is compelling, and the writing is clear. To firmly establish causality and mechanism, several links need strengthening.

Major Points
Current evidence relies on paralysis and fluorescence intensity. Please quantify aggregated vs soluble species to rule out expression/reporter artifacts-native/denaturing Western blots for Aβ/α-syn (or polyQ), including total and detergent-insoluble fractions. Filter-trap or dot-blot assays for high-MW aggregates. If using fluorescent reporters, measure mRNA (qPCR) and copy number to exclude expression differences.

To show H4K16 status drives UPRER and downstream phenotypes, engineer H4K16R (non-acetylatable) and H4K16Q (acetyl-mimic) knock-ins at a representative H4 locus; or assay xbp-1 splicing, hsp-4, fat-7, proteostasis. Complement with NuA4 catalytic-dead mys-1 and rescue lines.

Quantify spliced xbp-1 (xbp-1s) by RT-PCR/qPCR or use a xbp-1 splicing reporter. If feasible, an inducible xbp-1s (e.g., HS-xbp-1s) pulsed during development to test sufficiency for long-term proteostasis/lifespan benefits.

Why are fat-5/7 the primary outputs of SBP-1? Are co-factors required? Epistasis analysis with mdt-15, nhr-49, nhr-80 (RNAi or mutants) for fat-7, OA levels, proteostasis, and lifespan.

Given known HSF-1-lipid-proteostasis crosstalk, assess HSF-1 activity (HSF-1 target expression; hsf-1 RNAi or hsf-1(sy441) epistasis) to include/exclude its role.

Report dose-dependent response for OA on aggregation, UPRER markers, and lifespan. Discuss and statistically compare OA vs other FAs in Fig. 6K to clarify specificity (e.g., MUFA vs PUFA vs SFA).

Clarify whether protection involves storage vs utilization of MUFA accumulation. How MUFAs enhance proteostasis? If new data are not available, expand discussion with testable mechanisms and minimal assays:

Minor Points

Provide a supplementary table listing all primary hits (25 protective + 9 sensitizing) with scores, criteria, and secondary validation status.

Show whether early mys-1 perturbation also induces UPRmt (e.g., hsp-6p::GFP, hsp-60p::GFP) or cytosolic HSR (hsp-16.2p::GFP). This will help demonstrate ER specificity.

Early mys-1 RNAi may alter marks beyond H4K16ac. Please discuss this possibility and, if feasible, include targeted Westerns/CUT&Tag (e.g., H3K27ac, H3K9ac) during the critical window to acknowledge alternative or cooperating chromatin changes.

Point-by-point response to reviewers, EMBOJ-2025-122496

Referee #2 (Report for Author)

Reviewer #2 comment 1:

Health and longevity are tightly governed by a complex interplay of genetic and environmental factors; however, the underlying mechanisms are not fully clear. In this manuscript from the Wu group, they present a surprising and intriguing finding that proteostasis and lifespan of adult worms are predetermined by the activity of a MYST-family acetyltransferase (MYS-1) in early developmental stage. Through a combination of classical genetical manipulation strategies and deep-sequencing data analysis, they illustrated that deficiency of MYS-1 from embryo to L1 stage extended lifespan and conferred resistance to proteotoxicity in later-life via a chromatin-ER-lipid axis (or NuA4-H4K16ac-XBP-1-SBP-1-FAT-7 axis). This study clearly demonstrated that longevity and health are not only preprogrammed but also temporally regulated by the activity of specific genetic factors, making this manuscript interesting to many potential readers of EMBO J. I am thus happy to support its publication at EMBO J. Nevertheless, I have several comments that I hope the authors will find helpful to the paper if successfully addressed.

RESPONSE: We appreciate the reviewer's positive assessment of our work and thank them for the constructive comments. In this revised manuscript, we have addressed each point with additional experiments and clarifications, as detailed below.

Reviewer #2 comment 2:

1) Is there any tissue specificity for MYS-1 to regulate late-life proteostasis and longevity?

RESPONSE: We thank the reviewer for this insightful question, which prompted us to investigate the potential tissue-specificity of MYS-1's function. We approached this by examining MYS-1's expression pattern and performing tissue-specific protein depletion during the critical developmental window:

1) Expression Pattern Analysis: Using a functional GFP::MYS-1 knock-in line, we mapped MYS-1 expression during embryogenesis (PMID: 34312566). MYS-1 protein was broadly detected across multiple embryonic cell lineages, with elevated levels in cells fated to become gut and a subset of neurons (Main Text Fig. EV4A,B).

2) Tissue-Specific Protein Degradation: To functionally test tissue requirements, we induced MYS-1 degradation specifically during the embryo-to-L1 stage in either the intestine (*ges-1p*-driven TIR1) or neurons (*rgef-1p*-driven TIR1). Unlike the robust proteostasis improvement observed upon global (*eft-3p*-driven) MYS-1 depletion during this same window (Main Text

Figs. 1I and EV3B), tissue-restricted degradation in the intestine or neurons failed to recapitulate these adult phenotypes (Main Text Fig. EV4C-E).

Collectively, these results indicate that while MYS-1 expression is enriched in certain tissues (gut and neurons), its function in programming adult proteostasis and longevity likely requires coordinated action across multiple tissues to generate a systemic signal. These findings are now included in the revised manuscript (Lines 160-171).

Reviewer #2 comment 3:

2) Do the authors know the mechanisms by which XBP-1 regulates the subcellular location or activity of SBP-1?

RESPONSE: We thank the reviewer for this mechanistic question. Drawing on the established paradigm for SREBP (the mammalian orthologue of SBP-1), which requires COPII-dependent trafficking from ER to Golgi and its subsequent proteolytic cleavage for activation, we hypothesized that XBP-1-driven ER expansion might facilitate this process. Supporting this, we observed XBP-1-dependent ER expansion and whorl formation under early-life *mys-1* perturbation (Main Text Fig. 4F), promoting us to test whether this ER remodeling enhances SBP-1 maturation (Main Text Fig. 6F). Disrupting key steps in this pathway attenuated SBP-1 activation and its downstream effects. Knockdown of *sec-22*, a COPII-associated SNARE required for stress-induced ER remodeling, significantly reduced both *fat-7* upregulation and proteostasis improvement under early-life *mys-1* RNAi (Main Text Fig. 6G,H). Similarly, RNAi against *Y56A3A.2*, the *C. elegans* ortholog of the SREBP-cleaving protease, suppressed these phenotypes (Main Text Fig. 6G,H). These results support a model in which XBP-1-driven ER expansion enhances COPII-dependent trafficking and proteolytic maturation of SBP-1, thereby linking ER homeostasis to lipid metabolic regulation. This mechanism is now described in the revised manuscript (Lines 343-359).

Reviewer #2 comment 4:

3) What are the consequences of increasing the activity or expression of MYS-1 during early-life?

RESPONSE: We addressed this question through two complementary approaches. First, we generated a strain overexpressing *C. elegans mys-1* cDNA under the control of the *mex-5* promoter, which drives gene expression in the germline and early embryos. However, this overexpression did not detectably increase global H4K16ac levels in embryos compared to wild-type controls (Response Figure 1A). Second, we employed a newly reported CRISPR activation system (PMID: 35636511) via ingested sgRNAs targeting the endogenous *mys-1*

promoter, achieving approximately 1.5-fold increase in embryonic *mys-1* mRNA levels (Response Figure 1B). This transcriptional upregulation also failed to elevate H4K16ac levels (Response Figure 1C). The lack of H4K16ac increase in both scenarios suggests that MYS-1 expression within the NuA4 complex is not a simple limiting factor for setting physiological H4K16ac levels during this critical window. Its activity may be constrained by complex stoichiometry, co-factor availability, or counter-regulatory mechanisms. Since we did not observe the expected H4K16ac elevation, we did not pursue downstream functional assays in these strains.

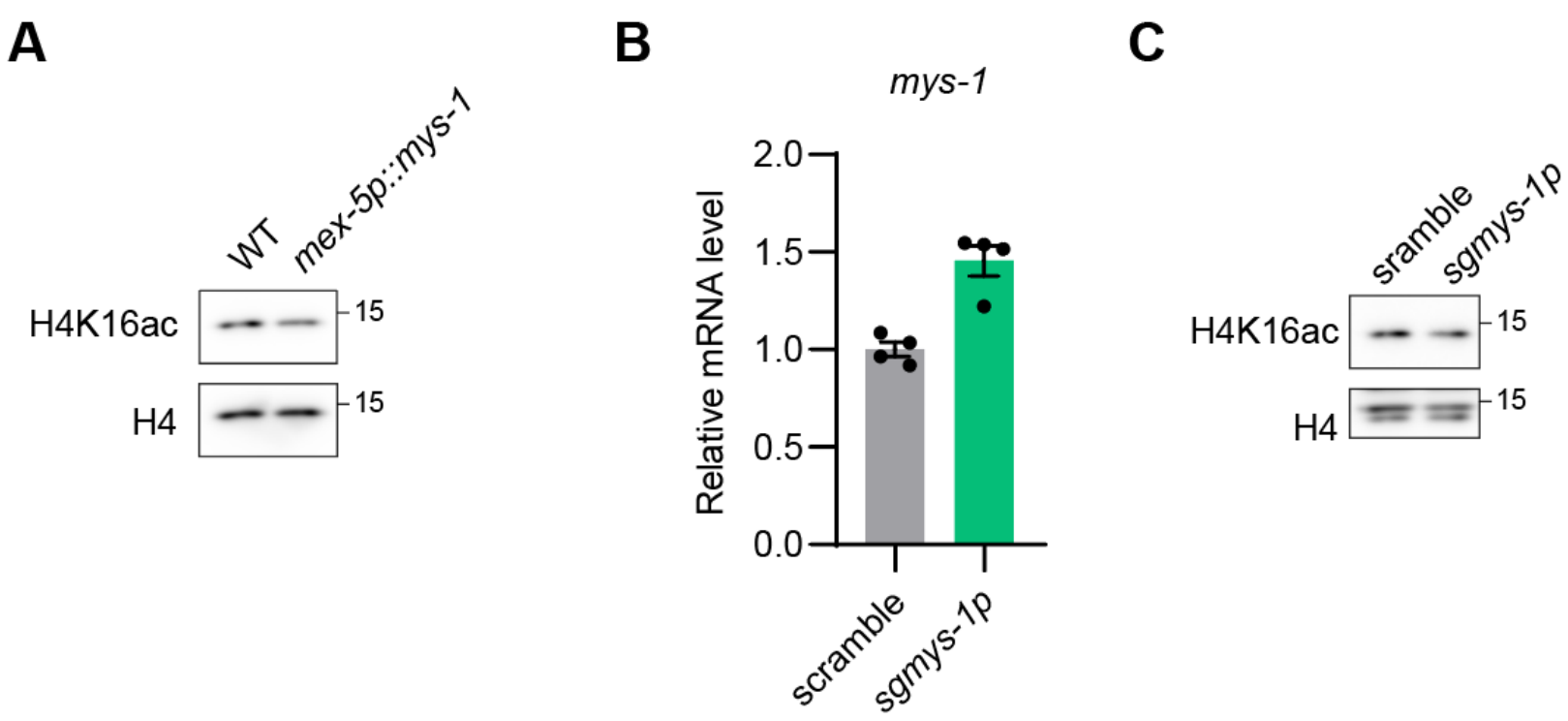

Response Figure 1. Forced overexpression of MYS-1 does not significantly change H4K16ac level. (A) Western blot analysis of H4K16ac level in WT and transgenic animals in E6 stage. (B) qPCR analysis of gene expression in E6 stage with CRISPR-based gene overexpression. (C) Western blot analysis of H4K16ac level in E6 stage using CRISPR-based gene overexpression strains.

Reviewer #2 comment 5:

Conversely, are there any detrimental effects from reducing its activity at this stage?

RESPONSE: Yes. Early-life *mys-1* perturbation caused developmental delay and reduced fecundity (Main Text Fig. EV2D,E). These results are incorporated in the revised manuscript (Lines 133-135).

Reviewer #2 comment 6:

4) Why is the embryo to L1 stage so critical for MYS-1 to induce adult proteotoxicity resistance and lifespan-extension?

RESPONSE: This is a central question. Our data delineate a signaling cascade (NuA4/H4K16ac → XBP-1 → SBP-1/MDT-15 → FAT-7) triggered by early-life MYS-1 perturbation. The functional output of this cascade is temporally restricted: *xbp-1* inhibition specifically during the L1 stage abolishes the long-term proteostasis benefits, while XBP-1 activation by MYS-1 depletion during this same window is sufficient to confer protection (Main

Text Fig. 5). This establishes the embryo-to-L1 stage as a critical period for XBP-1-dependent programming. This temporal requirement extends to the pathway's metabolic output. Oleic acid (OA) accumulation, a downstream consequence of the XBP-1–SBP-1/MDT-15 axis (Main Text Fig. EV10E), was only beneficial when OA supplementation begun at the L1 stage; supplementation from L4 onward failed to improve adult proteostasis (Main Text Fig. EV10H). Collectively, these results suggest the embryo-to-L1 stage represents a unique “window of opportunity” during which the NuA4/XBP-1 signal can be translated into durable metabolic changes (elevated OA), establishing a resilient cellular state that persists into adulthood. These results are now incorporated and discussed in the revised manuscript (Lines 404-415).

Reviewer #2 comment 7:

5) Do the authors know how oleic acid, produced by FAT-7, protect worms from proteotoxicity induced by Aβ1-42 in muscles?

RESPONSE: We thank the reviewer for this important question. Please see our detailed responses to **Reviewer #3 comment 12**, where we present new data investigating OA’s metabolic fate and functional role in proteostasis enhancement.

Referee #3 (Report for Author)

Reviewer #3 comment 1:

This manuscript identifies a developmental "chromatin-ER-lipid" axis in C. elegans: transient early-life reduction of NuA4 (mys-1) activity lowers H4K16ac, induces an XBP-1-dependent UPRER, remodels ER, activates SBP-1→FAT-5/7, elevates oleic acid (OA), and yields durable proteostasis benefits and lifespan extension. The story is compelling, and the writing is clear. To firmly establish causality and mechanism, several links need strengthening.

RESPONSE: We thank the reviewer for their thoughtful assessment and constructive feedback. We have systematically addressed each suggestion through new experiments and revised text, strengthening the manuscript substantially.

Reviewer #3 comment 2:

Major Points

Current evidence relies on paralysis and fluorescence intensity. Please quantify aggregated vs soluble species to rule out expression/reporter artifacts-native/denaturing Western blots for Aβ/α-syn (or polyQ), including total and detergent-insoluble fractions. Filter-trap or dot-blot assays for high-MW aggregates.

RESPONSE: We performed comprehensive biochemical analyses on both Aβ and α-synuclein models. For the muscle Aβ strain, native gel Western blot and filter-trap assays revealed that early-life *mys-1* RNAi markedly reduced high-molecular-weight and SDS-insoluble Aβ aggregates (Main Text Fig. EV5A). Similarly, in the muscle α-syn strain, native Western blot showed a shift towards lower-molecular-weight species and reduced high-molecular-weight α-syn aggregates following *mys-1* RNAi (Main Text Fig. EV5B). Crucially, the total protein levels measured by denaturing Western blot were unchanged in both models (Main Text Fig. EV5A,B), confirming that enhanced proteostasis network capacity, rather than reduced expression, underlies the protective effects. These results are now included in the revised manuscript (Lines 181-189).

Reviewer #3 comment 3:

If using fluorescent reporters, measure mRNA (qPCR) and copy number to exclude expression differences.

RESPONSE: We quantified the mRNA levels of Aβ and α-syn transgenes by qPCR and found no reduction following early-life *mys-1* RNAi (Main Text Fig. EV5C,D). Together with the biochemical data above, these results substantially rule out expression or reporter artifacts. These new data have been added to the revised manuscript (Lines 181-189).

Reviewer #3 comment 4:

To show H4K16 status drives UPRER and downstream phenotypes, engineer H4K16R (non-acetylatable) and H4K16Q (acetyl-mimic) knock-ins at a representative H4 locus; or assay xbp-1 splicing, hsp-4, fat-7, proteostasis.

RESPONSE: We thank the reviewer for this important point regarding causality. We agree that site-specific genetics is a powerful approach. It is worth noting, however, that implementing this in the context of *C. elegans* histone H4 presents a unique set of challenges. The presence of 16 identical H4 genes creates substantial functional redundancy (Response Figure 2A), and a constitutive knock-in would disrupt this essential modification throughout the entire lifespan, potentially confounding the analysis of our specific early-life programming phenotype. Despite these hurdles, we made a dedicated effort to test the hypothesis.

First, we successfully engineered H4K16R and H4K16Q knock-in alleles at the *his-64* locus, a representative H4 gene with high embryonic expression (Response Figure 2B). As expected, given the functional redundancy within the histone family, these endogenous knock-in strains did not significantly alter global H4K16ac levels or downstream signaling (Response Figures 2C and 2D).

To more directly test the role of H4K16ac, we employed a more refined strategy. We used *his-64* RNAi to knock down endogenous H4 levels, creating a sensitized background. In this background, we expressed RNAi-resistant transgenes (*his-64*)* specifically in the germline/early embryo to rescue with either wild-type H4 (*mex-5p::his-64*)* or the H4K16R mutant *(mex-5p::his-64*(K16R)*). As intended, the *his-64*(K16R)* transgene in the sensitized *his-64* RNAi background reduced the relative level of total H4K16ac (Response Figure 2E). Crucially, however, this global reduction did not recapitulate the key molecular signatures of *mys-1* perturbation: it failed to induce *xbp-1s*, *hsp-4* or *fat-7* (Response Figure 2F).

These results suggest a more nuanced regulatory model. While our data firmly establish that MYS-1 activity is required to set the H4K16ac level, the new genetic data indicate that the phenotypic consequences are not a simple function of bulk, global H4K16ac concentration. A primary interpretation is that MYS-1/NuA4 deposits H4K16ac at a critical set of genomic loci. Loss of *mys-1* depletes the mark specifically at these regulatory sites, triggering the signaling cascade. This aligns with our epigenomic findings: MYS-1/NuA4 deposits H4K16ac at promoters of key UPR^{ER}-related genes (Main Text Figs. 3D,E and EV6C,D) show that early-life *mys-1* depletion reduces H4K16ac specifically at these sites, which we propose sets the stage for the subsequent compensatory rebound in UPR^{ER} signaling (Main Text Fig. 3F,G). In contrast, the *his-64*(K16R) transgene may disrupt acetylation uniformly, failing to recreate the precise functional landscape. We also note that we cannot formally rule out contributions from other H4 acetylation sites, H4K8ac, H4K16ac, and H4 tetra-acetylation, which show correlated genomic distributions in embryos (PMID: 21177964). Nevertheless, the specificity of the H4K16ac reduction in our system, contrasted with unchanged H3 acetylation marks, strongly supports H4K16ac as a critical and representative component of the H4 tail acetylation program altered by MYS-1.

In summary, while directly proving causality via a single-site H4 mutant is highly challenging, our combined data, the specific loss of H4K16ac upon *mys-1* RNAi, the functional rescue by restoring acetylation (via *sin-3* RNAi), and now the dissociation of global acetylation from pathway output, collectively support a model where MYS-1-dependent H4 tail acetylation, with H4K16ac as a key reporter, at specific genomic coordinates gates the developmental reprogramming. We have incorporated this refined interpretation and the new experimental data into the revised manuscript, and we frame the challenge of locus-specific histone editing as an important future direction in the Discussion (Lines 473-489).

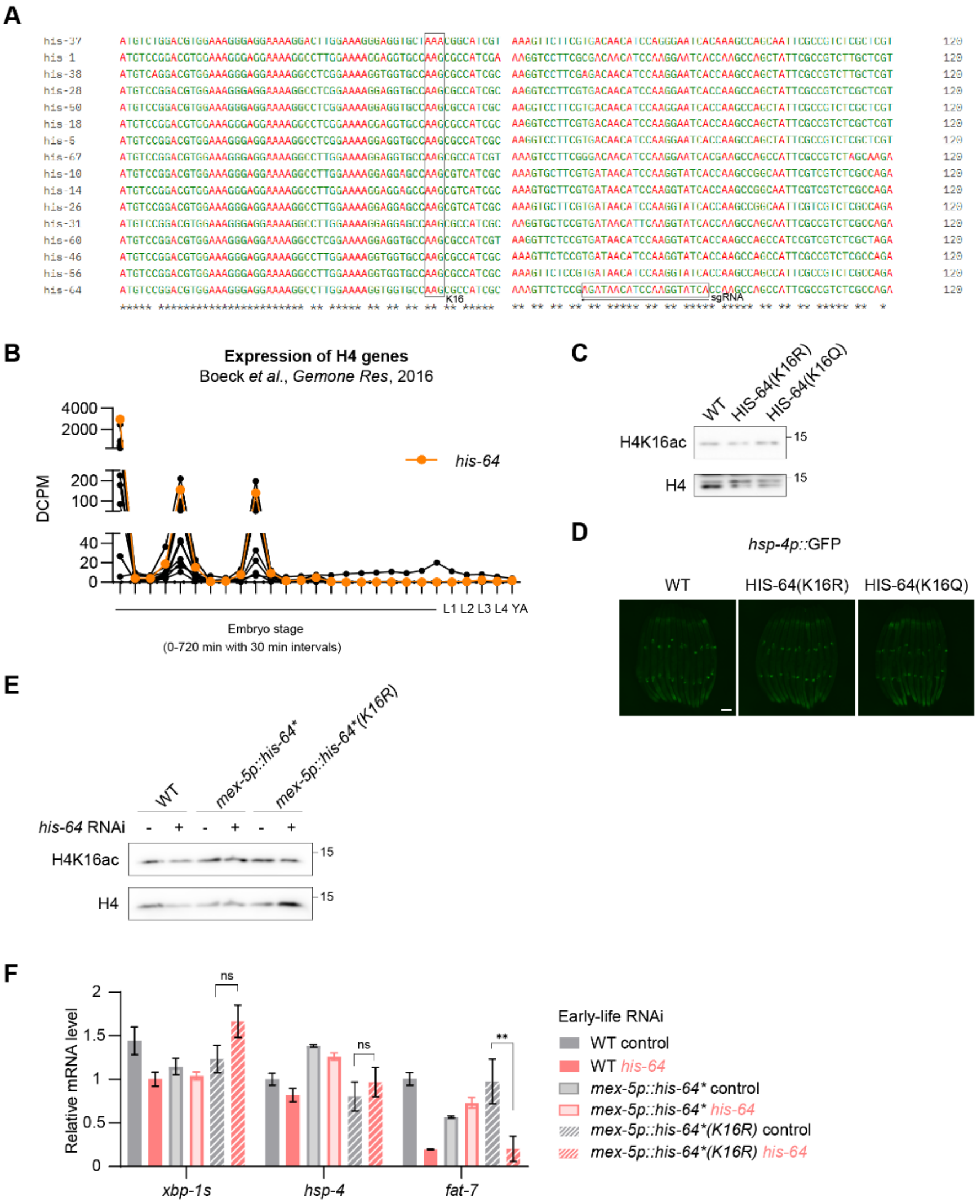

Response Figure 2. Attempts to engineer and characterize H4K16 site-specific mutants. (A) Similarity between coding sequences of all 16 H4-coding genes in the *C. elegans* genome. (B) The expression level of all 16 H4-coding genes during life course. (C) Western blot analysis of H4K16ac level in the HIS-64 mutants in E6 stage. (D) Representative images of *hsp-4p*::GFP animals with indicated genotype. Scale bar: 100 µm. (E) Western blot analysis of H4K16ac level in H4 RNAi-resistant transgenes (*his-64**). (F) Relative mRNA level of *xbp-1s*, *hsp-4* and *fat-7* in H4 RNAi-resistant transgenes (*his-64**).

Reviewer #3 comment 5:

Complement with NuA4 catalytic-dead mys-1 and rescue Lines.

RESPONSE: We made concerted efforts to generate a catalytic-dead *mys-1* allele by targeting three conserved residues (Q318E, G321E, E344Q) required for acetyltransferase activity in its orthologs (PMID: 17223684, PMID: 10966108, PMID: 28445719). Despite two independent

microinjection attempts, we obtained no viable knock-in animals. Given that the existing deletion allele *mys-1(n4075)* is homozygous lethal, this suggests that complete loss of catalytic function may also be lethal.

As an alternative approach, we generated transgenic lines expressing wild-type or catalytically-dead *C. briggsae mys-1* (Q321E, G324E, E347Q) under the *mex-5* promoter. The *C. briggsae* transgene is resistant to RNAi targeting the *C. elegans mys-1* sequence. This strategy yielded informative new results. In the *mys-1* RNAi background, germline expression of wild-type Cbr-MYS-1 led to a partial but significant recovery of H4K16ac levels, suggesting an incomplete but functional complementation. In contrast, expression of the catalytic-dead mutant failed to restore H4K16ac (Response Figure 3A). Crucially, this functional difference at the histone level translated directly to the physiological outputs. The same transgenic lines were tested in our phenotypic assays. The wild-type Cbr-MYS-1 transgene partially rescued the induction of *xbp-1s*, *hsp-4*, and *fat-7* triggered by *C. elegans mys-1* RNAi. It also conferred a partial but significant rescue of the improved proteostasis. Conversely, the catalytic-dead transgene failed to rescue molecular or phenotypic readouts (Response Figures 3B and 3C).

These new data provide direct evidence that the acetyltransferase activity of MYS-1 is required for its function in establishing the developmental H4K16ac level and for initiating the downstream UPR^{ER}-lipid signaling cascade that programs lifelong proteostasis. Combined with our data from the histone deacetylase experiment, the genetic interaction between MYS-1 (writer) and SIN-3 (eraser) demonstrates that the phenotypic consequences of *mys-1* depletion depend on the catalytic output of MYS-1 on its histone substrate. We have integrated the interpretation of these results into the revised Discussion (Lines 484-489).

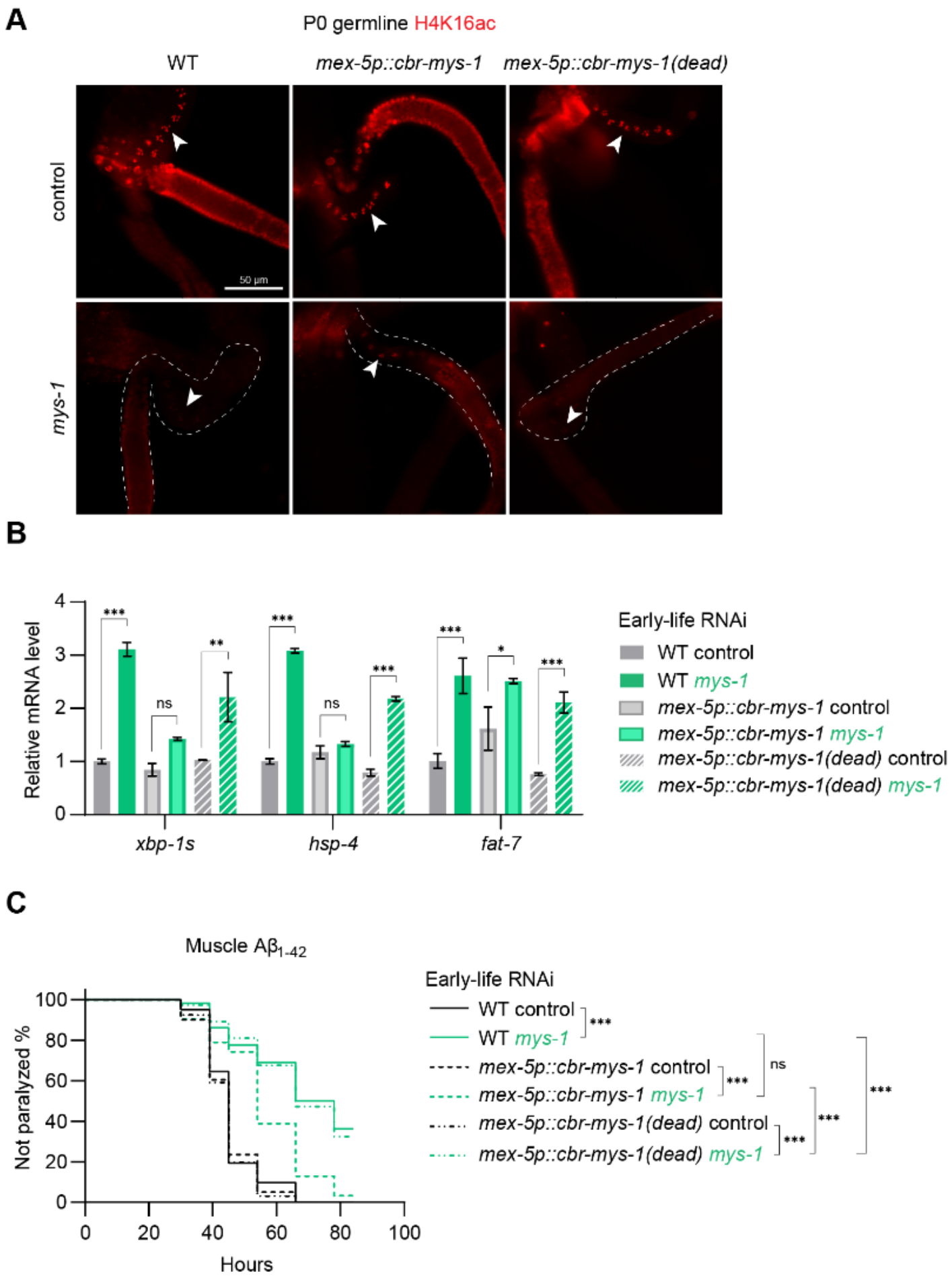

Response Figure 3. Further evidence confirms the specific function of early-life MYS-1 relies on acetyltransferase activity. (A) Immunostaining of the germline H4K16ac. White arrows indicated the H4K16ac signal in oocytes. Scale bar: 50 μm. (B) Relative mRNA level of *xbp-1s*, *hsp-4* and *fat-7* under early-life *mys-1* RNAi in WT and transgene-rescued animals. (C) Paralysis analysis of muscle A$\beta_{1\text{-}42}$ animals under early-life *mys-1* RNAi in WT and transgene-rescued background.

Reviewer #3 comment 6:

Quantify spliced xbp-1 (xbp-1s) by RT-PCR/qPCR or use a xbp-1 splicing reporter.

RESPONSE: We performed both experiments to quantify *xbp-1*splicing. Quantitative PCR analysis confirmed a significant increase in spliced *xbp-1s* in L1 animals following early-life *mys-1* RNAi, with total *xbp-1*mRNA levels unchanged (Main Text Fig. 4A). Using an *xbp-1* splicing reporter, in which GFP is in-frame with the spliced *xbp-1s* ORF, we observed increased nuclear GFP signal in a subset of L1 animals (Main Text Fig. 4B,C). Together, these data demonstrate that early-life *mys-1* depletion enhances *xbp-1* splicing and increases the levels of active XBP-1s protein. These results have been added to the revised manuscript (Lines 275-282).

Reviewer #3 comment 7:

If feasible, an inducible xbp-1s (e.g., HS-xbp-1s) pulsed during development to test sufficiency for long-term proteostasis/lifespan benefits.

RESPONSE: This is a thoughtful question. To address this question, we generated a heat-shock-inducible *xbp-1s* strain (*hsp-16.2p::xbp-1s::gfp*). A brief L1 heat shock robustly induced *xbp-1s* expression, with strong nuclear GFP signal that gradually subsided, confirming its successful pulsed activation (Response Figure 4A). This acute, high-level activation contrasts with the modest, mosaic activation observed with *mys-1* RNAi; instead, it exacerbated Aβ-induced paralysis in adulthood (Response Figure 4B).

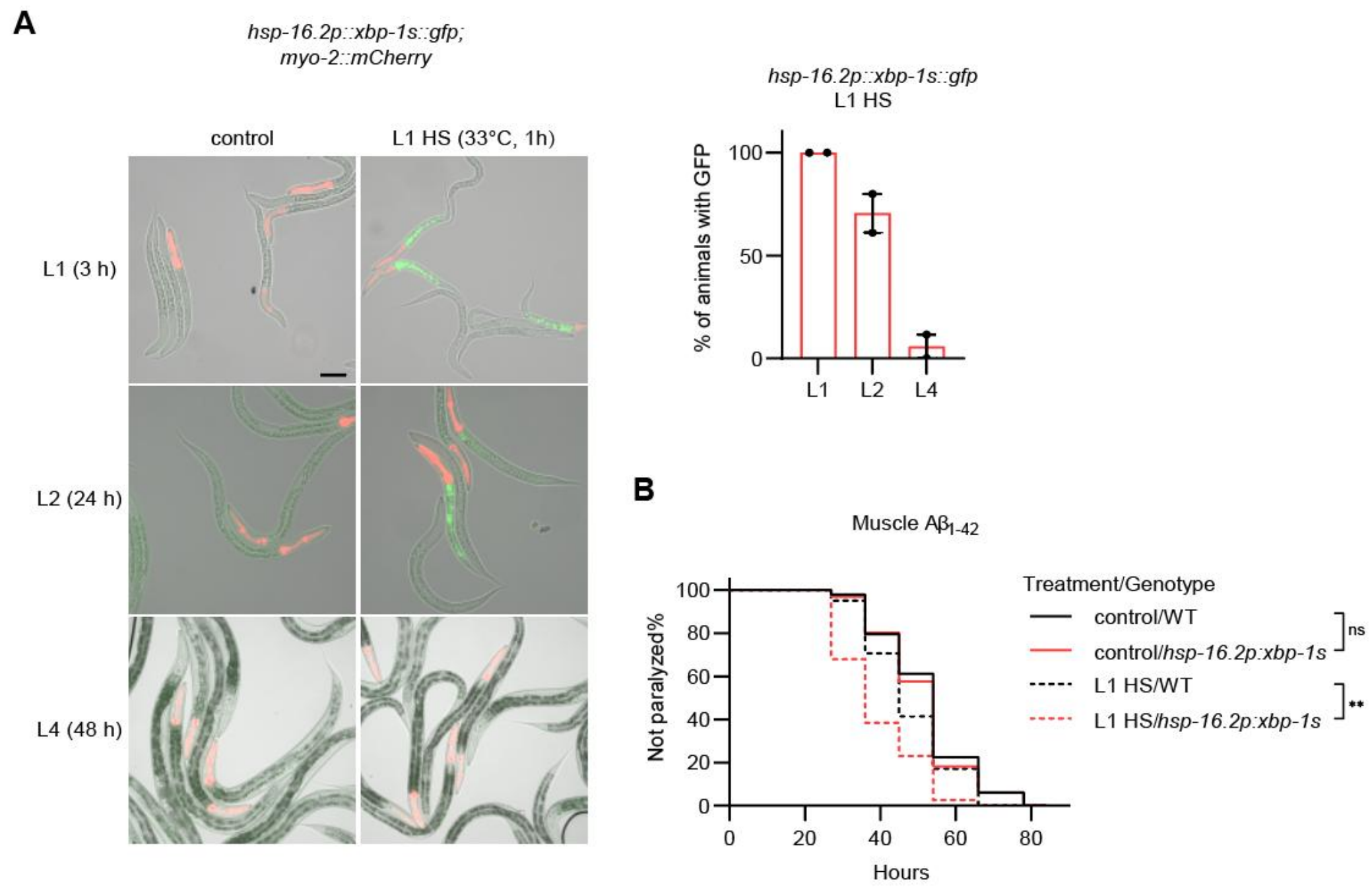

Response Figure 4. Heat-shock-induced *xbp-1s* overexpression in the L1 stage impairs late-life proteostasis resilience. (A) Representative images and quantifications of *hsp-16.2p::xbp-1s::gfp* animals Scale bar: 50 µm. (B) Paralysis analysis of muscle $A\beta_{1-42}$ animals with the indicated genotype and treatment.

This striking difference suggests that long-term functional outcomes depend not simply on XBP-1 activation but on its specific context, including magnitude, duration, and spatial pattern. The *xbp-1* splicing induced by *mys-1* RNAi was quantitatively modest (~1.5-2-fold increase) and spatially mosaic (~20% of animals showing reporter signal), representing a mild, integrated stress signal. A strong acute pulse may impose a transient burden that overshadows adaptive signaling. Future studies with more precisely controlled induction systems will be valuable. We have expanded the Discussion to consider these points (Lines 525-530).

Reviewer #3 comment 8:

Why are fat-5/7 the primary outputs of SBP-1? Are co-factors required? Epistasis analysis with mdt-15, nhr-49, nhr-80 (RNAi or mutants) for fat-7, OA levels, proteostasis, and lifespan.

RESPONSE: We performed the suggested genetic epistasis analyses using mutants of *nhr-49*, *nhr-80* and *mdt-15* and found that *nhr-49(ok2165)* and *nhr-80(tm1011)* mutants did not block *fat-7* induction or OA elevation (Main Text Figs. EV9B and EV10E) under early-life *mys-1* RNAi. In contrast, the *mdt-15(tm2182)* mutation, which disrupts a known co-activator of SBP-1 (PMID: 16799563), effectively suppressed both *fat-7* upregulation and OA increase (Main Text Figs. EV9B and EV10E), demonstrating specific requirement for the SBP-1/MDT-15 axis.

Functionally, *nhr-49* and *nhr-80* mutations did not block the proteostasis improvement conferred by early-life *mys-1* RNAi (Main Text Fig. EV9C,D), whereas *mdt-15* mutation effectively abolished it (Main Text Fig. EV9E). Consistently, *mdt-15(tm2182)* attenuated the lifespan extension conferred by early-life *mys-1* RNAi, particularly in the extension of maximum lifespan (Main Text Fig. EV9F). These new data confirm the SBP-1/MDT-15 axis specifically links chromatin-ER-lipid signaling to improved long-term health. These results have been incorporated into the revised manuscript (Lines 334-339, 361-364).

Reviewer #3 comment 9:

Given known HSF-1-lipid-proteostasis crosstalk, assess HSF-1 activity (HSF-1 target expression; hsf-1 RNAi or hsf-1(sy441) epistasis) to include/exclude its role.

RESPONSE: We systematically assessed HSF-1 involvement. Early-life *mys-1* RNAi did not alter HSF-1::GFP protein levels (Main Text Fig. EV9G) nor activate the canonical HSF-1 target *hsp-16.2p::GFP* (Main Text Fig. EV6C). Functionally, *hsf-1* RNAi did not suppress *fat-7* induction (Main Text Fig. EV9H), proteostasis improvement (Main Text Fig. EV9I), or lifespan extension conferred by *mys-1* RNAi (Main Text Fig. EV9J). Together, these results exclude HSF-1 as a necessary mediator (Lines 371-376).

Reviewer #3 comment 10:

Report dose-dependent response for OA on aggregation, UPRER markers, and lifespan.

RESPONSE: We supplemented diets with increasing OA concentrations (0, 0.2, 0.8, 2 mM). In muscle Aβ animals, OA improved proteostasis, with 0.8 mM OA providing optimal protection against paralysis (Main Text Fig. EV10B). OA extended lifespan in a dose-dependent fashion (Main Text Fig. EV10C) and did not affect UPR^{ER} activity at any concentration (Main Text Fig. EV10D), confirming it acts downstream of XBP-1. These data support 0.8 mM as an

effective concentration for health benefits observed in our study and are now included in the revised manuscript (Lines 397-402).

Reviewer #3 comment 11:

Discuss and statistically compare OA vs other FAs in Fig. 6K to clarify specificity (e.g., MUFA vs PUFA vs SFA).

RESPONSE: In the revised manuscript (now Main Text Fig. 7C), we have added statistical comparisons between OA and other fatty acids tested, confirming OA's significantly stronger protective effect. We have expanded the Discussion to cite literature on the distinct roles of unsaturated FAs (C18:1n9, C16:1, C18:1n7, C18:2) and consider why OA may be the critical mediator in this context (Lines 545-554).

Reviewer #3 comment 12:

Clarify whether protection involves storage vs utilization of MUFA accumulation. How MUFAs enhance proteostasis? If new data are not available, expand discussion with testable mechanisms and minimal assays:

RESPONSE: We thank the reviewer for this key question, also raised by **Reviewer #2 comment 7**. To investigate how OA enhances proteostasis, we first assessed its metabolic fate. Lipidomic analysis following early-life *mys-1* RNAi confirmed that OA incorporation into multiple lipid classes, including triglycerides (TGs) and phospholipids (Main Text Fig. 7F), indicating integration into both energy stores and membrane structures.

To determine if OA mobilization is functionally important, we examined lipolysis-related gene expression. RNA-seq of adult animals revealed significant upregulation of lipolysis genes (Main Text Fig. 7G). Supporting a functional role, RNAi against key lipases, adipose triglyceride lipase (*atgl-1*) and hormone-sensitive lipase (*hosl-1*), significantly attenuated the proteostasis improvement conferred by early-life *mys-1* RNAi (Main Text Fig. 7H,I).

These data support a model where developmental OA accumulation establishes a lipid landscape that supports elevated lipolytic flux in adulthood, with active lipid turnover, rather than simple OA storage, promoting proteostasis. These findings are now presented (Lines 417-429) and integrated into the Discussion (Lines 536-543).

Reviewer #3 comment 13:

Minor Points

Provide a supplementary table listing all primary hits (25 protective + 9 sensitizing) with scores, criteria, and secondary validation status.

RESPONSE: We apologize for the lack of clarity regarding our early-life RNAi screening methodology and criteria. In the revised manuscript, we have now included an explanatory description of our strategy (Lines 747-748) and provided the requested information in Table EV1.

Reviewer #3 comment 14:

Show whether early mys-1 perturbation also induces UPRmt (e.g., hsp-6p::GFP, hsp-60p::GFP) or cytosolic HSR (hsp-16.2p::GFP). This will help demonstrate ER specificity.

RESPONSE: We examined UPR^{mt} (*hsp-6p::*GFP) and cytosolic HSR (*hsp-16.2p::*GFP) reporters. Early-life *mys-1* RNAi induced only mild, transient UPR^{mt} activation at the L1 stage, with no persistent signal, and no detectable HSR activation (Main Text Fig. EV6C). In contrast, the UPR^{ER} reporter exhibited strong, sustained activation from early development through adulthood (Main Text Fig. EV6C), demonstrating specific and enduring UPR^{ER} engagement (Lines 228-235).

Reviewer #3 comment 15:

Early mys-1 RNAi may alter marks beyond H4K16ac. Please discuss this possibility and, if feasible, include targeted Westerns/CUT&Tag (e.g., H3K27ac, H3K9ac) during the critical window to acknowledge alternative or cooperating chromatin changes.

RESPONSE: We performed Western blot analysis of H3K9ac and H3K27ac in E0 and E6 embryos following early-life *mys-1* RNAi. Both marks remain unchanged (Main Text Fig. EV6A), in contrast to the significant H4K16ac reduction (Main Text Fig. 3B). This indicates specificity for H4K16ac rather than global loss of histone acetylation. We have incorporated these data and discuss that while H4K16ac is strongly implicated, technical challenges with H4 gene redundancy prevent complete exclusion of contributions from other co-regulated H4 acetylation sites (Lines 206-207 and 473-489).

Dear Dr. Wu,

Thank you for submitting a revised version of your manuscript. Your study has now been seen by all original referees, who find that their previous concerns have been addressed and now recommend publication of the manuscript. There remain only a few mainly editorial points that have to be addressed before I can extend formal acceptance of the manuscript:

- FUNDING INFO: missing in eJP: Zhejiang Provincial Natural Science Foundation of China under Grant (2022XHSJJ005), and by the Westlake Education Foundation; missing in ms: National Natural Science Foundation of China (32071151)

- Please rename the Conflict of Interest section into "Disclosure and Competing Interests Statement", in accordance with our updated Guide to Authors (https://link.springer.com/partners/embo-press/editorial-policies#Competing%20interest%20disclosures)

- As we are switching from a free-text author contribution statement towards a more formal statement based on Contributor Role Taxonomy (CRediT) terms, please remove the present Author Contribution section and instead specify each author's contribution(s) directly in the Author Information page of our submission system during upload of the final manuscript. See https://casrai.org/credit/ for more information.

- FIGURE CALLOUTS: missing callout for Fig. 7E

- DATASET EV LEGENDS: source file names, titles, legends and manuscript callouts all need to be updated to Dataset EV1-EV6 instead of Supplementary Table 1 and 6 and Supplemental Table 2-5 (Table EV1-EV6), legends should be removed from ms and uploaded as a separate tab/sheet in each Excel file

- Please provide the Reagent and Tools Table. For more information, please check https://media.springernature.com/original/springer-cms/rest/v1/content/27825802/data/v1

- SOURCE DATA: SD files provided with complete checklist, but Source data files need to be saved in a scheme one figure/folder and then uploaded as .zip files. E.g. all the Source data files for figure 1 need to be saved in a single folder and this needs to be zipped and then uploaded as "SD figure 1.zip" file. For EV and/or appendix figures, ZIP together all source data.

- Please provide suggestions for a short 'blurb' text prefacing and summing up the conceptual aspect of the study in two sentences (max. 250 characters), followed by 3-5 one-sentence 'bullet points' with brief factual statements of key results of the paper; they will form the basis of an editor-written 'Synopsis' accompanying the online version of the article. Please also provide an altered synopsis image, making sure that the aspect ratio conforms to our website's format - it should be exactly 550 pixels wide and 300 pixels high.

There is a potential cell reuse between Figure 5C first cell and Figure EV3C first cell which is not listed in the figure legend. Please state reuse explicitly in the figure legends.

- Figure Legends (main + EV):
1. Please note that the exact p values are not provided in the legends of figures 2C-E; 3B, I; 4A, C, D; 5D, F; 6C, D, E, G, H, I, J; 7C, D, F, H, I; EV2 B, C, D, E, F; EV5 D, EV6 E, EV7 C-E; EV9 B, C, D, E, F, I, J; EV10 E, G

2. Please indicate the statistical test used for data analysis in the legend of figure 3B

3. Please note that information related to n is missing in the legends of figures EV4 B, EV6 E.

4. Please note that the error bars are not defined in the legends of figures EV4 B, EV6 E.

5. Please note that the arrow heads are not defined in the legend of figure EV4 C.

- "Supplemental information" section should be removed

- Sections need to be named and the order should be corrected: Title page - Abstract - Keywords - Introduction - Results - Discussion - Methods - Data Availability - Acknowledgements - Disclosure and Competing Interests Statement - References - Figure Legends - Table(s) - Expanded View Figure Legends.

Please let me know if you have any questions regarding any of these points. You can use the link below to upload the revised files.
Thank you again for giving us the chance to consider your manuscript for The EMBO Journal. I look forward to receiving the final

version.
With best regards,
Cornelius Schneider

Cornelius Schneider, PhD
Editor | The EMBO Journal
c.schneider@embojournal.org

Please refer to our figure preparation guideline in order to ensure proper formatting and readability in print as well as on screen:

https://link.springer.com/journal/44318/submission-guidelines#cms-Figure-and-data-presentation

Use the link below to submit your revision:

https://emboj.msubmit.net/cgi-bin/main.plex

Referee #2:

The authors have addressed all my concerns. Happy to support its publication in EMBO J. Congratulations!

Referee #3:

I have no further questions and recommend acceptance of the manuscript.

Point-by-point response to the Editorial Team, EMBOJ-2025-122496R

- FUNDING INFO: missing in eJP: Zhejiang Provincial Natural Science Foundation of China under Grant (2022XHSJJ005), and by the Westlake Education Foundation; missing in ms: National Natural Science Foundation of China (32071151)

RESPONSE: The funding information has been updated in both the submission system and the manuscript.

- Please rename the Conflict of Interest section into "Disclosure and Competing Interests Statement", in accordance with our updated Guide to Authors (https://link.springer.com/partners/embo-press/editorial-policies#Competing%20interest%20disclosures)

RESPONSE: Changed accordingly.

- As we are switching from a free-text author contribution statement towards a more formal statement based on Contributor Role Taxonomy (CRediT) terms, please remove the present Author Contribution section and instead specify each author's contribution(s) directly in the Author Information page of our submission system during upload of the final manuscript. See https://casrai.org/credit/ for more information.

RESPONSE: The "Author Contribution" section has been removed from the manuscript. Author contributions have been specified using CRediT terms and entered directly into the submission system.

- FIGURE CALLOUTS: missing callout for Fig. 7E

RESPONSE: We apologize for this oversight and have added the callout for Fig. 7E to the main text where appropriate.

- DATASET EV LEGENDS: source file names, titles, legends and manuscript callouts all need to be updated to Dataset EV1-EV6 instead of Supplementary Table 1 and 6 and Supplemental Table 2-5 (Table EV1-EV6), legends should be removed from ms and uploaded as a separate tab/sheet in each Excel file

RESPONSE: updated as suggested.

- Please provide the Reagent and Tools Table. For more information, please check https://media.springernature.com/original/springer-cms/rest/v1/content/27825802/data/v1

RESPONSE: A “Reagent and Tools Table” file has been provided.

- SOURCE DATA: SD files provided with complete checklist, but Source data files need to be saved in a scheme one figure/folder and then uploaded as .zip files. E.g. all the Source data files for figure 1 need to be saved in a single folder and this needs to be zipped and then uploaded as "SD figure 1.zip" file. For EV and/or appendix figures, ZIP together all source data.

RESPONSE: Source data and checklist have been organized accordingly (including all main and EV figures).

- Please provide suggestions for a short 'blurb' text prefacing and summing up the conceptual aspect of the study in two sentences (max. 250 characters), followed by 3-5 one-sentence 'bullet points' with brief factual statements of key results of the paper; they will form the basis of an editor-written 'Synopsis' accompanying the online version of the article. Please also provide an altered synopsis image, making sure that the aspect ratio conforms to our website's format - it should be exactly 550 pixels wide and 300 pixels high.

RESPONSE: We have prepared draft texts for the ‘blurb’ and the bullet-point list of key results, as provided below for your consideration.

Blurb:

How early-life events program lifelong health is largely unclear. This study shows that reducing NuA4 complex activity during development durably enhances proteostasis and lifespan by triggering a UPR^{ER}–lipid priming axis in *C. elegans*.

Bullet Points:

- Early-life, but not later-life, knockdown of NuA4 subunits protects against Aβ and α-synuclein proteotoxicity and extends lifespan in *C. elegans*.
- Early-life reduction of NuA4-mediated H4K16ac elicits compensatory activation of the unfolded protein response (UPR) transcription factor XBP-1.
- XBP-1 activation remodels ER morphology and drives SBP-1/MDT-15–dependent FAT-7 upregulation, leading to selective oleic acid (OA) accumulation during development.
- Developmentally accumulated OA confers lasting proteostasis resilience in adulthood.

Synopsis image:

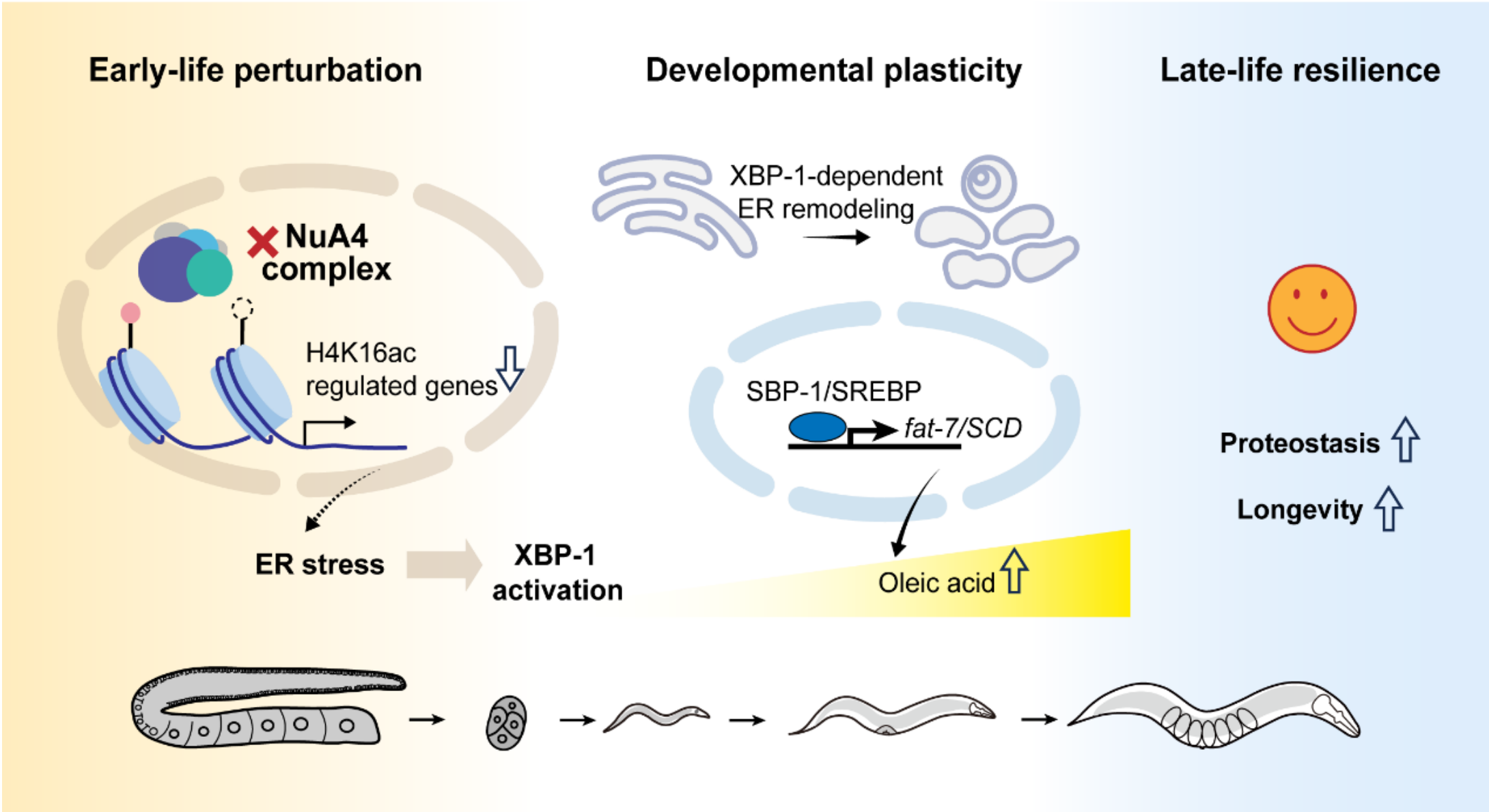

- There is a potential cell reuse between Figure 5C first cell and Figure EV3C first cell which is not listed in the figure legend. Please state reuse explicitly in the figure legends.

RESPONSE: Thank you for noting this. We have verified the figures and identified that the image reuse is between Figure 5C and Figure EV6C (not EV3C as mentioned, which we appreciate you highlighting for our review). We have explicitly stated this reuse in the legends of both figures. As noted, these panels represent the identical control condition used in two independent experimental setups. The declaration has been added to ensure full transparency.

- Figure Legends (main + EV):

1. Please note that the exact p values are not provided in the legends of figures 2C-E; 3B, I; 4A, C, D; 5D, F; 6C, D, E, G, H, I, J; 7C, D, F, H, I; EV2 B, C, D, E, F; EV5 D, EV6 E, EV7 C-E; EV9 B, C, D, E, F, I, J; EV10 E, G

2. Please indicate the statistical test used for data analysis in the legend of figure 3B

3. Please note that information related to n is missing in the legends of figures EV4 B, EV6 E.

4. Please note that the error bars are not defined in the legends of figures EV4 B, EV6 E.

5. Please note that the arrow heads are not defined in the legend of figure EV4 C.

RESPONSE: We have comprehensively revised the presentation of statistical information and experimental details across all figure legends to enhance clarity and data transparency. Specifically:

- In place of statistical significance markers (ns, *, **) and in response to the request for exact p-values, we have directly annotated all graphs with the exact p-values. We hope this provides readers with immediate, quantitative access to statistical results directly on

the figures.

- To ensure each data representation is fully self-contained, we have restructured the figure legends to integrate all critical information (including the statistical test, n number, error bar definitions, arrowheads annotations) directly following the description of each relevant panel. This replaces the previous format where such details were consolidated at the end of the legend. The missing information of the mentioned figures (3B, EV4 B, EV4 C, EV6 E) is now clearly provided in their respective panel legend.

- "Supplemental information" section should be removed

RESPONSE: The section has been removed.

- Sections need to be named and the order should be corrected: Title page - Abstract - Keywords - Introduction - Results - Discussion - Methods - Data Availability - Acknowledgements - Disclosure and Competing Interests Statement - References - Figure Legends - Table(s) - Expanded View Figure Legends.

RESPONSE: The manuscript sections have been reordered and renamed as suggested.

Dear Dr. Wu,

I am pleased to inform you that your manuscript has been accepted for publication in the EMBO Journal.

Your manuscript will be processed for publication by EMBO Press. It will be copy edited and you will receive page proofs prior to publication. Please note that you will be contacted by Springer Nature Author Services to complete licensing and payment information.

You may qualify for financial assistance for your publication charges - either via a Springer Nature fully open access agreement or an EMBO initiative. Check your eligibility: https://link.springer.com/journal/44318/how-to-publish-with-us

Should you be planning a Press Release on your article, please notify the production team via this form in order to coordinate publication and release dates: https://forms.cloud.microsoft/e/eH4A8JVP6g

If you have any questions, please do not hesitate to contact the Editorial Office. Thank you for your contribution to The EMBO Journal.

Yours sincerely,

Cornelius Schneider, PhD
Editor
The EMBO Journal
c.schneider@embojournal.org

*****
Please note that it is The EMBO Journal policy for the transcript of the editorial process (containing referee reports and your response letters) to be published as an online supplement to each paper. If you should prefer removal of any referee-only figures included in the point-by-point response(s), e.g. because they may still be used for future publication or because they have been reproduced from published work by others, please do let us know immediately via response email.
More information is available here: https://link.springer.com/partners/embo-press/editorial-policies#Peer%20review

** Click here to be directed to your login page: https://emboj.msubmit.net

EMBO Press Author Checklist

Corresponding Author Name: Lianfeng Wu	
Journal Submitted to: The EMBO Journal	
Manuscript Number: EMBOJ-2025-122496R	

USEFUL LINKS FOR COMPLETING THIS FORM
The EMBO Journal - Author Guidelines
EMBO Reports - Author Guidelines
Molecular Systems Biology - Author Guidelines
EMBO Molecular Medicine - Author Guidelines

Reporting Checklist for Life Science Articles (updated January
This checklist is adapted from Materials Design Analysis Reporting (MDAR) Checklist for Authors. MDAR establishes a minimum set of requirements in transparent reporting in the life sciences (see Statement of Task: 10.31222/osf.io/9sm4x). Please follow the journal's guidelines in preparing your
Please note that a copy of this checklist will be published alongside your article.

Abridged guidelines for figures
1. Data
The data shown in figures should satisfy the following conditions:
- the data were obtained and processed according to the field's best practice and are presented to reflect the results of the experiments in an accurate and unbiased manner.
- ideally, figure panels should include only measurements that are directly comparable to each other and obtained with the same assay.
- plots include clearly labeled error bars for independent experiments and sample sizes. Unless justified, error bars should not be shown for technical
- if n<5, the individual data points from each experiment should be plotted. Any statistical test employed should be justified.
- Source Data should be included to report the data underlying figures according to the guidelines set out in the authorship guidelines on Data

2. Captions
Each figure caption should contain the following information, for each panel where they are relevant:
- a specification of the experimental system investigated (eg cell line, species name).
- the assay(s) and method(s) used to carry out the reported observations and measurements.
- an explicit mention of the biological and chemical entity(ies) that are being measured.
- an explicit mention of the biological and chemical entity(ies) that are altered/varied/perturbed in a controlled manner.
- the exact sample size (n) for each experimental group/condition, given as a number, not a range;
- a description of the sample collection allowing the reader to understand whether the samples represent technical or biological replicates (including how many animals, litters, cultures, etc.).
- a statement of how many times the experiment shown was independently replicated in the laboratory.
- definitions of statistical methods and measures:
  - common tests, such as t-test (please specify whether paired vs. unpaired), simple χ2 tests, Wilcoxon and Mann-Whitney tests, can be unambiguously identified by name only, but more complex techniques should be described in the methods section;
  - are tests one-sided or two-sided?
  - are there adjustments for multiple comparisons?
  - exact statistical test results, e.g., P values = x but not P values < x;
  - definition of 'center values' as median or average;
  - definition of error bars as s.d. or s.e.m.

Please complete ALL of the questions below.
Select "Not Applicable" only when the requested information is not relevant for your study.

Materials

Newly Created Materials	Information included in the manuscript?	In which section is the information available? (Reagents and Tools Table, Materials and Methods, Figures, Data Availability Section)
New materials and reagents need to be available; do any restrictions apply?	Not Applicable	

Antibodies	Information included in the manuscript?	In which section is the information available? (Reagents and Tools Table, Materials and Methods, Figures, Data Availability Section)
For **antibodies** provide the following information:<br>- Commercial antibodies: RRID (if possible) or supplier name, catalogue number and or/clone number<br>- Non-commercial: RRID or citation	Yes	Reagents and tools table

DNA and RNA sequences	Information included in the manuscript?	In which section is the information available? (Reagents and Tools Table, Materials and Methods, Figures, Data Availability Section)
**Short novel DNA or RNA including primers, probes**: provide the sequences.	Yes	Reagents and tools table

Cell materials	Information included in the manuscript?	In which section is the information available? (Reagents and Tools Table, Materials and Methods, Figures, Data Availability Section)
**Cell lines:** Provide species information, strain. Provide accession number in repository **OR** supplier name, catalog number, clone number, and/**OR** RRID.	Not Applicable	
**Primary cultures:** Provide species, strain, sex of origin, genetic modification status.	Not Applicable	
Report if the cell lines were recently **authenticated** (e.g., by STR profiling) and tested for mycoplasma contamination.	Not Applicable	

Experimental animals	Information included in the manuscript?	In which section is the information available? (Reagents and Tools Table, Materials and Methods, Figures, Data Availability Section)
**Laboratory animals or Model organisms:** Provide species, strain, sex, age, genetic modification status. Provide accession number in repository **OR** supplier name, catalog number, clone number, **OR** RRID.	Yes	Reagents and tools table
**Animal observed in or captured from the field:** Provide species, sex, and age where possible.	Not Applicable	
Please detail **housing and husbandry conditions**.	Not Applicable	

Plants and microbes	Information included in the manuscript?	In which section is the information available? (Reagents and Tools Table, Materials and Methods, Figures, Data Availability Section)
**Plants:** provide species and strain, ecotype and cultivar where relevant, unique accession number if available, and source (including location for collected wild specimens).	Not Applicable	
**Microbes:** provide species and strain, unique accession number if available, and source.	Yes	Reagents and tools table

Human research participants	Information included in the manuscript?	In which section is the information available? (Reagents and Tools Table, Materials and Methods, Figures, Data Availability Section)
If collected and within the bounds of privacy constraints report on age, sex and gender or ethnicity for all study participants.	Not Applicable	

Core facilities	Information included in the manuscript?	In which section is the information available? (Reagents and Tools Table, Materials and Methods, Figures, Data Availability Section)
If your work benefited from core facilities, was their service mentioned in the acknowledgments section?	Yes	Acknowledgements

Design

Study protocol	Information included in the manuscript?	In which section is the information available? (Reagents and Tools Table, Materials and Methods, Figures, Data Availability Section)
If study protocol has been **pre-registered, provide DOI in the manuscript**. For clinical trials, provide the trial registration number **OR** cite DOI.	Not Applicable	
Report the **clinical trial registration number** (at ClinicalTrials.gov or equivalent), where applicable.	Not Applicable	

Laboratory protocol	Information included in the manuscript?	In which section is the information available? (Reagents and Tools Table, Materials and Methods, Figures, Data Availability Section)
Provide DOI OR other citation details if **external detailed step-by-step protocols** are available.	Not Applicable	

Experimental study design and statistics	Information included in the manuscript?	In which section is the information available? (Reagents and Tools Table, Materials and Methods, Figures, Data Availability Section)
Include a statement about **sample size** estimate even if no statistical methods were used.	Yes	Methods
Were any steps taken to minimize the effects of subjective bias when allocating animals/samples to treatment (e.g. **randomization procedure**)? If yes, have they been described?	Yes	Methods
Include a statement about **blinding** even if no blinding was done.	Yes	Methods
Describe **inclusion/exclusion criteria** if samples or animals were excluded from the analysis. Were the criteria pre-established?<br><br>If sample or data points were omitted from analysis, report if this was due to attrition or intentional exclusion and provide justification.	Yes	Methods
For every figure, are **statistical tests** justified as appropriate? Do the data meet the assumptions of the tests (e.g., normal distribution)? Describe any methods used to assess it. Is there an estimate of variation within each group of data? Is the variance similar between the groups that are being statistically compared?	Yes	Figure legends

Sample definition and in-laboratory replication	Information included in the manuscript?	In which section is the information available? (Reagents and Tools Table, Materials and Methods, Figures, Data Availability Section)
In the figure legends: state number of times the experiment was **replicated** in laboratory.	Yes	Figure legends
In the figure legends: define whether data describe **technical or biological replicates**.	Yes	Figure legends

Ethics

Ethics	Information included in the manuscript?	In which section is the information available? (Reagents and Tools Table, Materials and Methods, Figures, Data Availability Section)
Studies involving **human participants**: State details of **authority granting ethics approval** (IRB or equivalent committee(s), provide reference number for approval.	Not Applicable	
Studies involving **human participants**: Include a statement confirming that **informed consent** was obtained from all subjects and that the experiments conformed to the principles set out in the WMA Declaration of Helsinki and the Department of Health and Human Services Belmont Report.	Not Applicable	
Studies involving **human participants:** For publication of **patient photos**, include a statement confirming that consent to publish was obtained.	Not Applicable	
Studies involving experimental **animals**: State details of **authority granting ethics approval** (IRB or equivalent committee(s), provide reference number for approval. Include a statement of compliance with ethical regulations.	Not Applicable	
Studies involving **specimen and field samples:** State if relevant **permits** obtained, provide details of authority approving study; if none were required, explain why.	Not Applicable	

Dual Use Research of Concern (DURC)	Information included in the manuscript?	In which section is the information available? (Reagents and Tools Table, Materials and Methods, Figures, Data Availability Section)
Could your study fall under dual use research restrictions? Please check biosecurity documents and list of **select agents and toxins** (CDC): https://www.selectagents.gov/sat/list.htm	Not Applicable	
If you used a select agent, is the security level of the lab appropriate and reported in the manuscript?	Not Applicable	
If a study is subject to dual use research of concern regulations, is the name of the **authority granting approval and reference number** for the regulatory approval provided in the manuscript?	Not Applicable	

Reporting

The MDAR framework recommends adoption of discipline-specific guidelines, established and endorsed through community initiatives. Journals have their own policy about requiring specific guidelines and recommendations to complement MDAR.

Adherence to community standards	Information included in the manuscript?	In which section is the information available? (Reagents and Tools Table, Materials and Methods, Figures, Data Availability Section)
State if relevant guidelines or checklists (e.g., **ICMJE, MIBBI, ARRIVE, PRISMA**) have been followed or provided.	Not Applicable	
For **tumor marker prognostic studies,** we recommend that you follow the **REMARK** reporting guidelines (see link list at top right). See author guidelines, under 'Reporting Guidelines'. Please confirm you have followed these guidelines.	Not Applicable	
For **phase II and III randomized controlled trials**, please refer to the **CONSORT** flow diagram (see link list at top right) and submit the CONSORT checklist (see link list at top right) with your submission. See author guidelines, under 'Reporting Guidelines'. Please confirm you have submitted this list.	Not Applicable	

Data Availability

Data availability	Information included in the manuscript?	In which section is the information available? (Reagents and Tools Table, Materials and Methods, Figures, Data Availability Section)
Have **primary datasets** been deposited according to the journal's guidelines (see 'Data Deposition' section) and the respective accession numbers provided in the Data Availability Section?	Yes	Data Availability
Were **human clinical and genomic datasets** deposited in a public access-controlled repository in accordance to ethical obligations to the patients and to the applicable consent agreement?	Not Applicable	
Are **computational models** that are central and integral to a study available without restrictions in a machine-readable form? Were the relevant accession numbers or links provided?	Not Applicable	
If publicly available data were reused, provide the respective **data citations in the reference list**.	Not Applicable	
